# Probing Ferryl Reactivity in a Nonheme Iron Oxygenase Using an Expanded Genetic Code

**DOI:** 10.1021/acscatal.4c02365

**Published:** 2024-07-20

**Authors:** Florence J. Hardy, Matthew G. Quesne, Emilie F. Gérard, Jingming Zhao, Mary Ortmayer, Christopher J. Taylor, Hafiz S. Ali, Jeffrey W. Slater, Colin W. Levy, Derren J. Heyes, J. Martin Bollinger, Sam P. de Visser, Anthony P. Green

**Affiliations:** † Department of Chemistry & Manchester Institute of Biotechnology, The University of Manchester, 131 Princess Street, Manchester M1 7DN, U.K.; ‡ Research Complex at Harwell, Rutherford Appleton Laboratory, Harwell Oxford, Didcot, Oxon OX11 0FA, U.K.; § School of Chemistry, Cardiff University, Main Building, Park Place, Cardiff CF10 3AT, U.K.; ∥ Department of Chemistry and Department of Biochemistry and Molecular Biology, The Pennsylvania State University, University Park, Pennsylvania 16802, United States; ⊥ Department of Chemical Engineering & Manchester Institute of Biotechnology, The University of Manchester, 131 Princess Street, Manchester M1 7DN, U.K.

**Keywords:** metal-oxo reactivity, C−H functionalization, genetic code expansion, noncanonical histidine analogue, 2OG-dependent hydroxylation

## Abstract

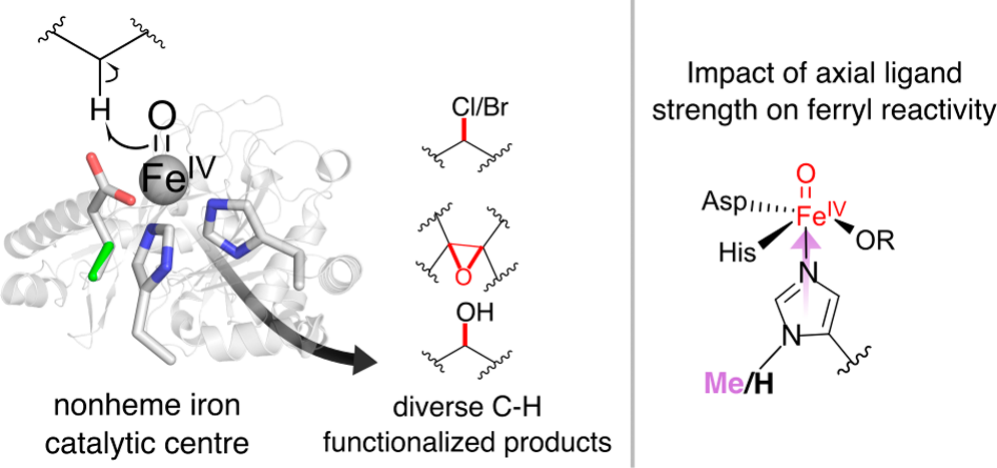

The ability to introduce noncanonical amino acids as axial ligands in heme enzymes has provided a powerful experimental tool for studying the structure and reactivity of their Fe^IV^=O (“ferryl”) intermediates. Here, we show that a similar approach can be used to perturb the conserved Fe coordination environment of 2-oxoglutarate (2OG) dependent oxygenases, a versatile class of enzymes that employ highly-reactive ferryl intermediates to mediate challenging C–H functionalizations. Replacement of one of the cis-disposed histidine ligands in the oxygenase VioC with a less electron donating *N*_δ_-methyl-histidine (MeHis) preserves both catalytic function and reaction selectivity. Significantly, the key ferryl intermediate responsible for C–H activation can be accumulated in both the wildtype and the modified protein. In contrast to heme enzymes, where metal-oxo reactivity is extremely sensitive to the nature of the proximal ligand, the rates of C–H activation and the observed large kinetic isotope effects are only minimally affected by axial ligand replacement in VioC. This study showcases a powerful tool for modulating the coordination sphere of nonheme iron enzymes that will enhance our understanding of the factors governing their divergent activities.

## Introduction

**Figure 1:**
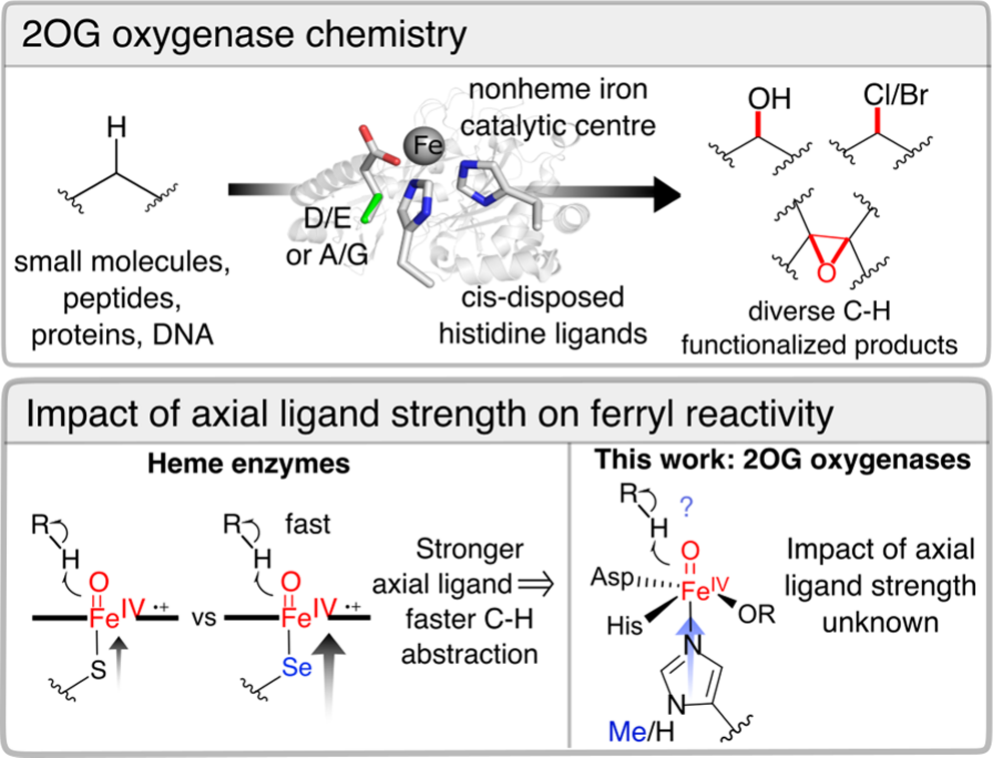
2OG oxygenases catalyze a wealth of oxidative chemistries. The effect of axial ligand substitutions is well-understood in heme enzymes, although the impact of axial ligand strength on nonheme iron 2OG oxygenases is unknown.

Iron^II^/2-oxoglutarate (2OG)-dependent dioxygenases are a versatile superfamily of enzymes that promote a wealth of selective C–H activation processes, including substrate hydroxylation, halogenation, C–C-forming cyclization, epoxidation, and desaturation.^1−11^ The catalytic capabilities of these enzymes have been expanded to include nonbiological processes, such as nitration and azidation.^12,13^ The catalytic Fe^II^ center is coordinated by a conserved His···Glu/Asp···His facial binding triad (Figure 1).^14−17^ In the case of halogenases and engineered azidation/nitration biocatalysts, the carboxylate is replaced by a noncoordinating residue (typically Ala/Gly), which leaves a vacant coordination site for anion binding.^13,18−20^ Reaction of the ferrous enzyme with molecular oxygen and 2OG generates the highly oxidizing ferryl (Fe^IV^=O) intermediate, which effects selective H atom abstractions (HA) from bound substrates.^21,22^ Depending on the specific enzyme in question, the resulting Fe^III^–OH/R^•^ intermediate can then undergo a variety of radical coupling processes, leading to distinct reaction outcomes.^6,20^

Given the broad array of valuable transformations catalyzed by 2OG oxygenases, there is considerable interest in understanding how their active site features govern the structure and reactivity of catalytic intermediates. To this end, a range of spectroscopic, kinetic, structural, and computational studies have advanced our understanding of the 2OG oxygenase catalytic cycles.^23−37^ Similarly, mutations of second and outer coordination sphere residues have advanced our understanding of 2OG oxygenase catalysis.^38^ However, performing structure–function relationships to probe the role of the conserved bis-histidine coordination environment has proven challenging, as mutation of either of the cis-disposed ligands abolishes catalytic function. If we were able to make functional ligand replacements, we could begin to address important questions such as the effect of ligand strength on Fe^IV^=O reactivity or positioning (“in-line” vs “off-line”^39,40^) across family members. Previously, our group and others have shown that the axial ligands of heme enzymes can be replaced by noncanonical analogues of cysteine and histidine, creating a new way to probe structure–activity relationships directly in enzyme active sites.^41−44,48^ Here, we show that a similar approach can be used to modulate the primary coordination sphere of nonheme iron enzymes, providing new opportunities to probe and augment the catalytic mechanisms of this enzyme superfamily.

We selected the 2OG-dependent hydroxylase VioC as a model enzyme for active site reengineering. VioC catalyzes a regio- and stereoselective hydroxylation of its amino acid substrate, l-arginine, to form 3(*S*)-hydroxy-l-arginine, a vital step in the biosynthesis of the nonribosomal peptide tuberactinomycin antibiotic, viomycin.^45,46^ The catalytic iron is coordinated by His168, Asp170, and His316, with His316 trans to the site at which oxygen is proposed to bind.^45,47^

## Results

**Figure 2:**
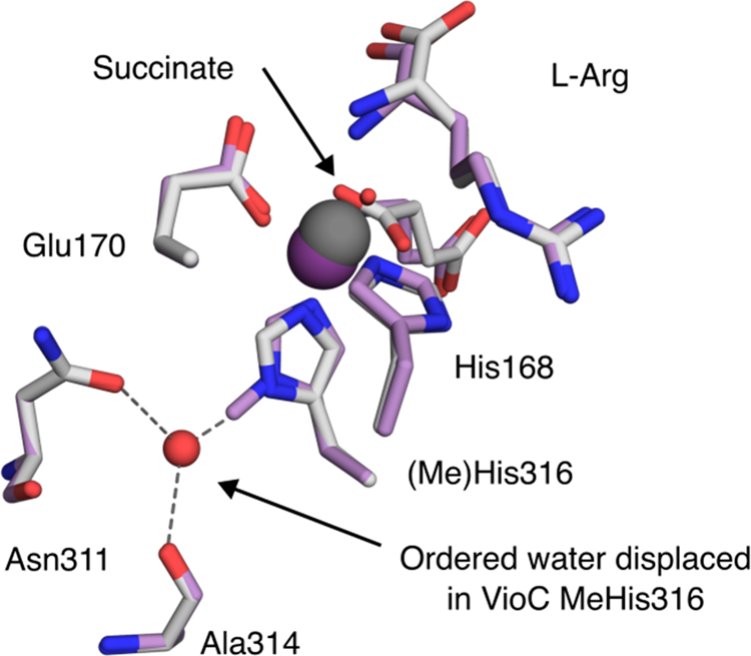
Structural characterization of VioC MeHis316. The active sites of VioC (PDB: 6ALQ) and VioC MeHis316 (PDB: 9EQF). Active site residues, succinate, and bound substrate l-Arg are shown as atom-colored sticks (gray and purple carbons for VioC and VioC MeHis316, respectively). Iron is shown as dark gray or dark purple spheres in VioC (occupancy 1) and VioC MeHis316 (occupancy 0.4), respectively. An ordered water molecule that coordinates the N_δ_ of His316 (shown as a red sphere) is only present in the structure of VioC.

To probe the effect of axial ligand electron donation, we replaced His316 by a noncanonical *N*_δ_-methyl-histidine (MeHis) ligand using an engineered pyrrolysyl-*t*RNA synthetase/pyrrolysyl-*t*RNA pair (PylRS_MeHis/tRNAPyl), which selectively encodes MeHis in response to the amber (UAG) stop codon.^49^ Stoichiometric replacement of histidine by MeHis was confirmed by mass spectrometry (Supporting Table S1). A 1.6 Å X-ray crystal structure of VioC MeHis316 (Supporting Table S2) superimposes well with a published wildtype structure (secondary structure superposed RMSD of 0.2 Å),^47^ with no significant changes in the location of the iron cofactor or the imidazole planes of the axial His/MeHis ligands (Figures 2 and S1). Introduction of the additional methyl substituent on the axial ligand displaces an ordered water, which, in wildtype VioC, forms hydrogen bonding interactions with the N_δ_-H of His316, the Asn311 side chain, and the backbone carbonyl of Ala314. The substrate l-arginine (occupancy 0.8) and succinate (occupancy 0.8) adopt similar conformations to those observed in the wildtype structure, with the l-Arg pro-3*S* hydrogen apparently well-positioned for selective removal by the ferryl complex.

**Figure 3:**
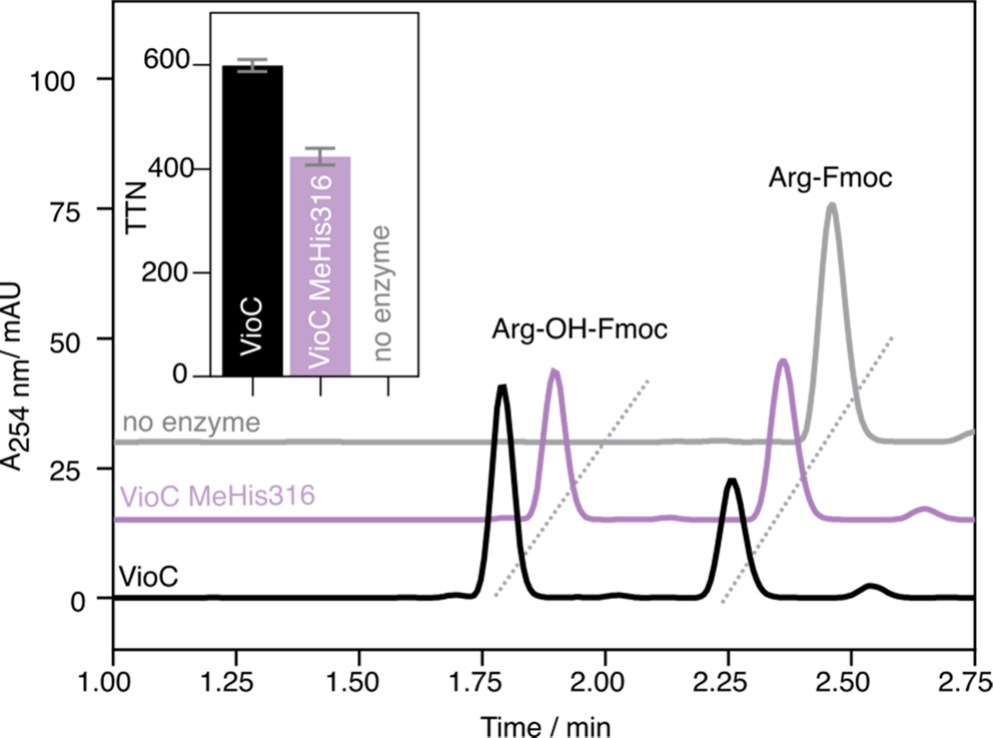
HPLC analysis of VioC-catalyzed hydroxylation. HPLC trace at 254 nm showing Fmoc-derivatized substrate Arg-Fmoc and product Arg-OH-Fmoc. Reaction conditions: 1 μM enzyme, 1 mM Arg, 5 mM 2OG, 1 mM (NH_4_)_2_Fe(SO_4_)_2_(H_2_O)_6_, 1 mM Asc in 100 μL 100 mM Tris pH 7.5, 18 h shaking (300 rpm) at 25 °C. Fmoc derivatization was achieved by the addition of 100 μL of 5 mM Fmoc-OSu in ACN. (inset) The total turnover numbers achieved by VioC (black) and VioC MeHis316 (purple) after 18 h shaking. Measurements are given as the average of four replicates with the standard deviation presented as an error bar.

We next explored the impact of ligand substitution on l-arginine hydroxylase activity. Assays were performed under standard conditions (1 mM Fe^II^, 1 mM l-Arg, 5 mM 2OG, and 1 mM sodium ascorbate, 25 °C) using either wildtype VioC (1 μM) or VioC MeHis316 (1 μM) as a biocatalyst. The conversion of l-arginine to 3(*S*)–OH-l-arginine was monitored by reverse-phase high-performance liquid chromatography (HPLC) following derivatization with *N*-(9-fluorenylmethoxycarbonyloxy)succinimide (Fmoc-OSu). The wildtype enzyme achieved ∼600 turnovers under these conditions, a value in line with previous reports.^50−52^ Remarkably, despite mutation of the primary iron coordination sphere, VioC MeHis316 was able to perform >400 turnovers, with no observed change in regioselectivity (Figure 3). Product retention times in biotransformations with VioC MeHis316 are indistinguishable from those observed with the wildtype enzyme, suggesting that the high degree of site selectivity achieved by wildtype VioC is retained upon axial ligand substitution, as anticipated, given the similar l-arginine conformations observed in the wildtype and VioC MeHis316 crystal structures.

With a functional modified enzyme in hand, we were well placed to investigate the effect of electron donation by the axial ligand on the VioC catalytic mechanism. To this end, we created cluster models of the ferryl intermediates of VioC and VioC MeHis316 that include first and second coordination sphere of the metal and substrate (Supporting Figure S2). Replacing His316 and its coordinating water with an axial MeHis ligand has little effect on ferryl structure, Fe–N bond length, electronic configuration, and spin state ordering (Supporting Tables S3 and S4). Introduction of the MeHis ligand increases the electron affinity of the ferryl intermediate slightly (ΔΔ*E* = 1.3 kcal mol^–1^, Supporting Table S5). In both systems, *in silico* reduction results in electron transfer into the virtual σ*_z_2 orbital along the Fe-His316 bond. These calculations suggest that MeHis is a less electron donating ligand than the native histidine, which is polarized by hydrogen bonding to an ordered water (Supporting Table S5). Analogous calculations performed on the wildtype enzyme in the absence of this ordered water molecule confirm that hydrogen bonding interactions with His316 increase its electron donating properties, as evidenced by an increase in ferryl electron affinity (Supporting Table S5). These trends observed with VioC and VioC MeHis316 are in agreement with our previous observations in heme proteins, where polarized His ligands have been shown to be more electron-donating than MeHis in both peroxidases and myoglobin.^41,44^

**Figure 4:**
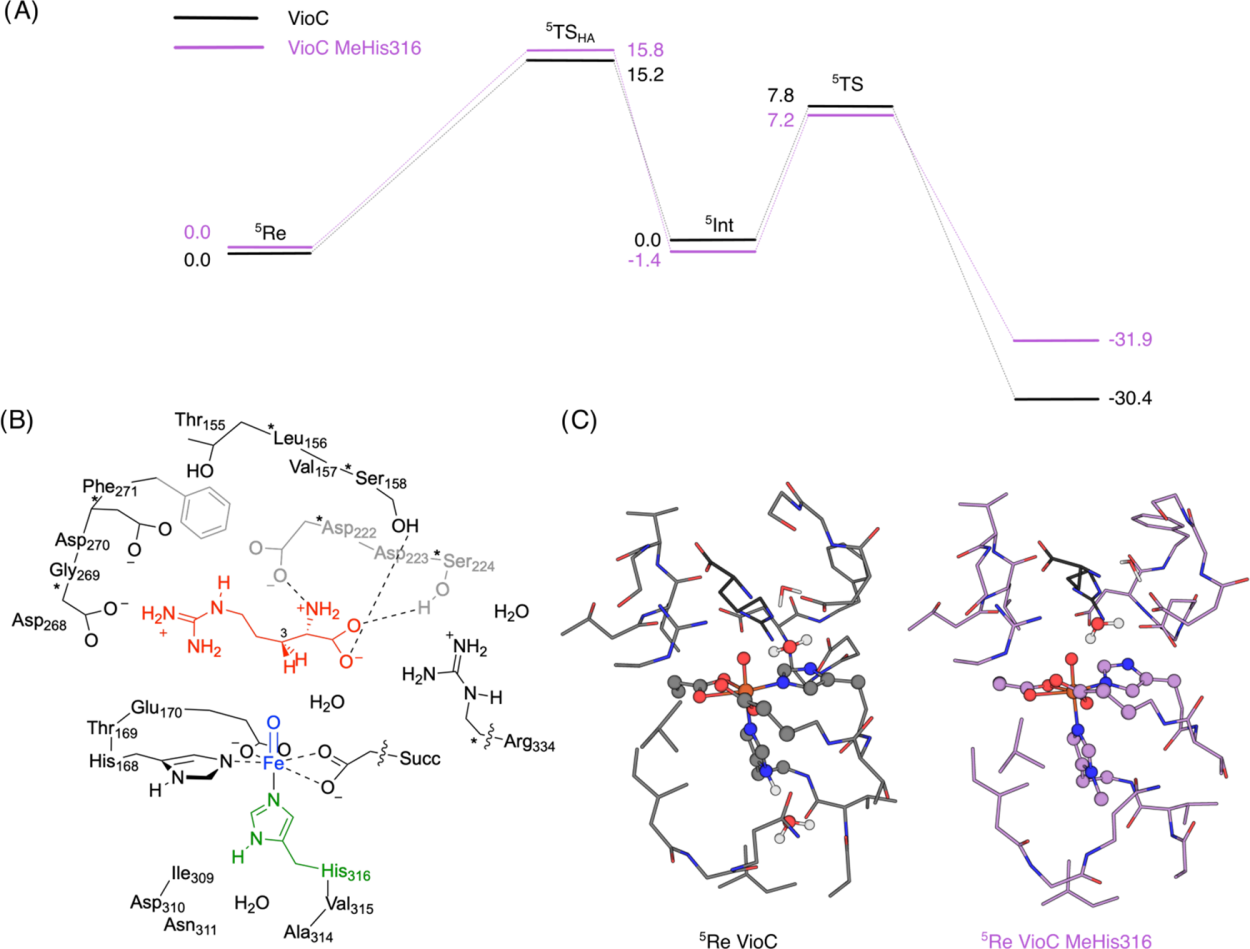
Computational analysis of VioC MeHis316. (A) Energy profile for hydrogen abstraction and rebound steps in the l-Arg hydroxylation reaction by VioC (black) and VioC MeHis316 (purple). UB3LYP/BS2 energies with zero-point energies included in kcal mol^–1^. (B) The QM cluster model B of the ferryl intermediate of wildtype VioC, generated to probe the energy landscapes for l-Arg hydroxylation. (C) The optimized geometries of model B in VioC (left) and VioC MeHis316 (right) in the ^5^Re (reactant) state.

To further explore the impact of ligand substitution on ferryl reactivity, we calculated the energy landscapes for C3-hydroxylation of l-arginine by VioC and VioC MeHis316. In this case we generated a larger cluster model, to include a greater number of residues interacting with the arginine substrate (Figure 4). Large cluster models of this type were previously shown to reproduce experimental product distributions and selectivities well.^53,54^ Recent computational studies on nonheme iron dioxygenases showed that experimentally determined free energies of activation and regioselectivities can only be reproduced with either large QM cluster models or QM/MM with a large QM region, hence the former approach was used here.^55^ Density functional theory (DFT) calculations were performed on the active-site models using unrestricted hybrid density functional UB3LYP in combination with a LANL2DZ basis set on iron (with core potential) and 6-31G* on the rest of the atoms (basis set BS1) (see Supporting Methods for further details).^56−60^ In agreement with previous calculations and experimental EPR/Mössbauer studies on the ferryl species of VioC and related nonheme iron dioxygenases the ground state is the quintet spin state.^61−67^ The hydrogen atom abstraction step is rate-determining with a free energy of activation of Δ*G* = 15.2 kcal mol^–1^ for VioC and Δ*G* = 15.8 kcal mol^–1^ for VioC MeHis316. The subsequent OH rebound leads to hydroxyarginine products with large exothermicity. These calculations show that the energy barrier for C–H cleavage is minimally affected by the His316MeHis modification (ΔΔ*E* = 0.6 kcal mol^–1^, Figure 4 and Supporting Table S6). Both HA transition-state structures have large imaginary frequencies and indeed, replacing the transferring hydrogen atom by deuterium gives a large predicted kinetic isotope effect (KIE), suggesting a large quantum chemical tunnelling contribution to the C–H abstraction step (Supporting Table S7 and Figure S5).

**Figure 5:**
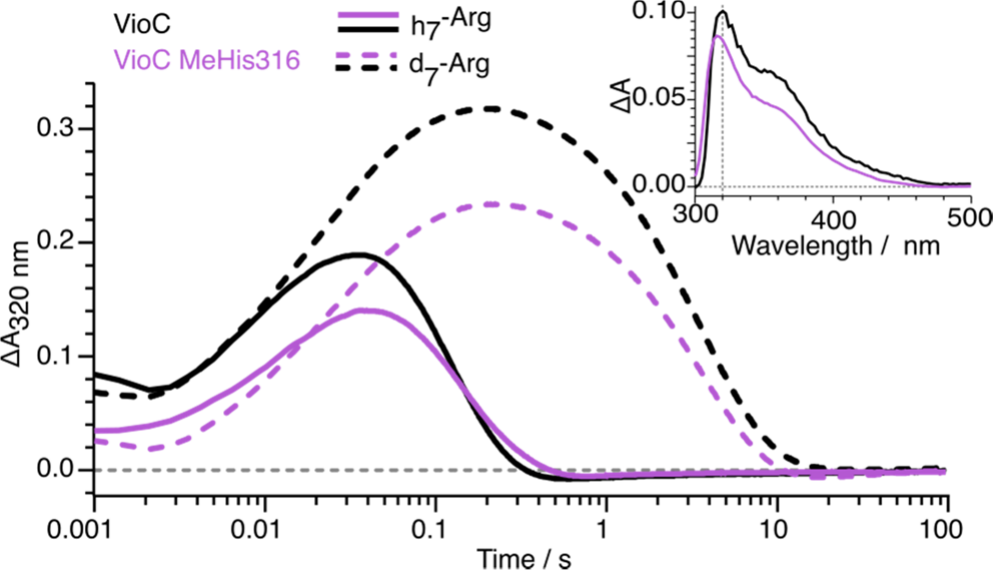
Stopped-flow UV–visible absorption analysis. Kinetics of formation and decay of the ferryl intermediates in the reactions of VioC (black) and VioC MeHis316 (purple) with l-Arg (solid lines) and per-*d*_7_-l-Arg (dashed lines) monitored by the absorbance changes at λ = 320 nm. Absorbance changes shown are the average of triplicate measurements. An anoxic solution of enzyme in complex with (per-*d*_7_-)-l-Arg and 2OG was mixed at 5 °C with an equal volume of 5 °C air-saturated buffer, to give a final concentration of 500 μM enzyme, 3 mM 2OG, 3 mM l-Arg or per-*d*_7_-l-Arg, and 500 μM (NH_4_)_2_Fe(SO_4_)_2_(H_2_O)_6_. (inset) The difference spectra of ferryl intermediates observed with VioC (black) and VioC MeHis316 (purple) 100 ms after rapid mixing a substrate complex (enzyme:Fe^II^:*d*_7_-l-Arg:2OG) with air-saturated buffer.

To probe the effect of ligand substitution on ferryl reactivity experimentally, we next used stopped-flow absorption measurements to define the kinetics of C–H cleavage in VioC and VioC MeHis316. Spectral changes were monitored following exposure of an anoxic reactant complex (enzyme:Fe^II^:2OG:Arg) to molecular oxygen within air-saturated buffer. Consistent with previous reports, VioC exhibits accumulation of a transient ferryl intermediate, as evidenced by the emergence of a broad absorption feature centered at 320 nm that reaches maximum intensity at ∼50 ms (Figure 5).^61^ Time-dependent absorbance changes were fitted to a sequential *a* → *b* → *c* model (See Supporting Information) to derive rate constants for the formation and decay of the ferryl intermediate in wildtype VioC (*k*_form,obs_ = 41.2 ± 0.7 s^–1^ and *k*_H_ = 11.9 ± 0.1 s^–1^, respectively; Tables 1 and S8). Experiments performed with per-*d*_7_-l-Arg lead to an extended lifetime of the ferryl intermediate (Figure 5 and Table 1), which was found to decay with a rate constant (*k*_D_) of 0.28 ± 0.002 s^–1^ to give a kinetic isotope effect (KIE = *k*_D_/*k*_H_) of 43.2 ± 0.7 for C–H/C–D abstraction (cf. a KIE of 43 reported previously, Supporting Table S9). Analogous experiments with VioC MeHis316 also reveal the formation of a ferryl intermediate with spectral features that are almost indistinguishable from those seen with the wildtype enzyme (Figure 5 inset). Remarkably, introduction of the less electron donating MeHis ligand has minimal effect on the rate constants of ferryl formation (*k*_form,obvs_ = 46.8 ± 1.0 s^–1^), the rate of C–H abstraction (*k*_H_ = 8.3 ± 0.3 s^–1^) and the observed KIE (28.9 ± 0.9). The modest reduction in KIE observed with VioC MeHis316 could plausibly arise from changes in the electronic structure of the ferryl intermediate and/or from minor adjustments in substrate positioning or active site dynamics. This result is in stark contrast to the situation encountered in heme enzymes, where there is a strong correlation between axial ligand strength and ferryl reactivity.^43,44^ Plausibly, the differing response to ligand substitution originates in the large contribution of quantum tunnelling to C–H abstraction by 2OG oxygenases, as evidenced by the high KIE.

**Table 1:** Stopped-flow Kinetic Analysis of VioC and VioC MeHis316^a^

	VioC		VioC MeHis316	
	*h*_7_-l-Arg	*d*_7_-l-Arg	*h*_7_-l-Arg	*d*_7_-l-Arg
*k*_H_ or *k*_D_, s^–1^	11.9 ± 0.1	0.28 ± 0.01	8.3 ± 0.1	0.29 ± 0.01
KIE	43 ± 1		29 ± 1	

a The rates of C–H abstraction given with protonated and deuterated l-Arg as the substrate are the average of triplicate measurements.

## Conclusions

In summary, we have demonstrated that an expanded genetic code enables perturbation of the primary coordination sphere of nonheme iron enzymes while maintaining catalytic function. This approach enables new probes of structure–function relationships directly in enzyme active sites that were previously not possible within the constraints of the genetic code. Against our expectations, modulation of axial ligand strength has minimal effect on the kinetics of C–H/D abstraction, with large kinetic isotope effects (30–40) observed in both wildtype VioC and VioC MeHis316, consistent with a substantial tunnelling contribution in both proteins. Moving forward, there are clear opportunities to expand our methodology to explore the impact of equatorial ligand substitutions on 2OG oxygenase catalysis. Moreover, we anticipate that our ability to desymmetrize the bis-histidine coordination sphere of nonheme iron enzymes will open new avenues to characterizing key metal-oxo intermediates (e.g., through selective isotopic labeling) or perhaps even unlock alternative modes of reactivity in this versatile superfamily of enzymes.

## Supporting Information Available

Additional experimental details, materials, and methods, additional references, fits of stopped-flow data to kinetic models, protein mass spectrometry, data collection and refinement statistics for structure determination of VioC MeHis316, and the Cartesian coordinates of the DFT models A and B (PDF)

## Abbreviations

HPLC: high performance liquid chromatography
DFT: density functional theory
