# Supplementary material for: Probing Ferryl Reactivity in a Nonheme Iron Oxygenase Using an Expanded Genetic Code
Source: ACS Catal. 2024 Jul 20;14(15):11584–90. doi: 10.1021/acscatal.4c02365 (PMC11301626; doi:10.1021/acscatal.4c02365)
Supplement: Supplementary file 1 — cs4c02365_si_001.pdf [file cs4c02365_si_001.pdf]

Supporting Information

Probing Ferryl Reactivity in a Nonheme Iron Oxygenase Using an Expanded Genetic Code

Florence J. Hardy,[1*] Matthew G. Quesne,[2,3] Emilie F. Gérard,[1] Jingming Zhao,[1] Mary Ortmayer,[1] Christopher J. Taylor,[1] Hafiz S. Ali,[1] Jeffrey W. Slater,[4] Colin W. Levy,[1] Derren J. Heyes,[1] J. Martin Bollinger Jr.,[4] Sam P. de Visser,[5] Anthony P. Green[1*]

[1]Department of Chemistry & Manchester Institute of Biotechnology, The University of Manchester, 131 Princess Street, Manchester, M1 7DN, (U.K.)

[2]Research Complex at Harwell, Rutherford Appleton Laboratory, Harwell Oxford, Didcot, Oxon, OX11 0FA, U.K.

[3]Cardiff University, School of Chemistry, Main Building, Park Place, Cardiff, CF10 3AT, (U.K.)

[4] Department of Chemistry and Department of Biochemistry and Molecular Biology, The Pennsylvania State University, University Park, Pennsylvania 16802 (U.S.A.)

[5]Department of Chemical Engineering & Manchester Institute of Biotechnology, The University of Manchester, 131 Princess Street, Manchester, M1 7DN, (U.K.)

* florence.hardy@manchester.ac.uk *anthony.green@manchester.ac.uk

Methods

Materials

All materials were obtained from Sigma-Aldrich unless otherwise stated. H-His(3-Me)-OH (MeHis) was purchased from Bachem. Oligonucleotides were synthesized by IDT. *Escherichia coli* (*E. coli*) 5α, *E. coli* BL21 (DE3), Q5 DNA polymerase, T4 DNA ligase and restriction enzymes were purchased from New England BioLabs. Per-d_7-L-Arginine was purchased from Cambridge Isotope Labs.

Construction of plasmids

The His316MeHis mutation was introduced into a pET-28c plasmid harbouring the gene encoding VioC (pET-28c_VioC) by replacing the His316 codon with a TAG stop codon using overlap assembly protocols (primers are given in Supplementary Table 10) and EcoRI and XhoI restriction sites to generate pET-28c_VioC_TAG316. A strep-II tag was introduced to replace the His_6-tag using complementary primers (Supplementary Table 10) into pET-28c_VioC and pET-28c_VioC_TAG316 using Gibson assembly, to give pET-28s_VioC and pET-28s_VioC_TAG316. Correct DNA sequences were confirmed by DNA sequencing using T7 and T7_term primers (Source BioScience).

Protein production and purification

For expression of VioC, pET-28c/s_VioC was transformed into *E. coli* BL21 (DE3) and the cells were plated onto an LB agar plate containing 50 µg ml^{-1} kanamycin. A single colony of freshly transformed cells was used to inoculate 10 mL of LB medium containing 50 µg ml^{-1} kanamycin and cultured at 37 °C with 180 rpm shaking for 18 h. 2 mL of the starter culture was used to inoculate 400 mL of bespoke medium (10 g L^{-1} tryptone, 5 g L^{-1} yeast extract, 2 mM $MgSO_4$, 0.5% (w/v) glucose, 8.95 g L-1 Na_2HPO_4, 3.4 g L^{-1} KH_2PO_4, 2.67 g L^{-1} NH_4Cl, 0.71 g L^{-1} Na_2SO_4) containing 50 µg ml^{-1} kanamycin. The cultures were incubated for ~2 h at 37 °C with 180 rpm shaking until the OD_{600} reached ~ 0.6, at which point IPTG was added to a final concentration of 200 µM. The cultures were incubated for a further ~20 h at 22 °C and the cells were subsequently harvested by centrifugation at 4000 g for 12 min.

For expression of VioC MeHis316, pET-28_VioC_TAG316 were co-transformed into *E. coli* BL21 (DE3) with pEVOL_MeHis[16] and the cells plated onto an LB agar place containing 50 µg ml^{-1} kanamycin and 34 µg ml^{-1} chloramphenicol. A single colony of freshly transformed cells was used to inoculate 10 mL of LB medium containing 50 µg ml^{-1} kanamycin and 34 µg ml^{-1}

chloramphenicol and cultured at 37 °C with 180 rpm shaking for 18 h. 2 mL of the starter culture was used to inoculate 400 mL of bespoke medium (as above) containing 6 mM MeHis, 50 µg ml^{-1} kanamycin and 34 µg ml^{-1} chloramphenicol. The cultures were incubated for ~2 h at 37 °C with 180 rpm shaking until the OD_{600} reached ~ 0.6, at which point IPTG and L-arabinose were added to final concentrations of 200 µM and 0.05% (w/v), respectively. The cultures were incubated for a further ~20 h at 22 °C and the cells were subsequently harvested by centrifugation at 4000 g for 12 min.

For purification of strep-tagged VioC and VioC MeHis316, the pelleted bacterial cells were suspended in buffer NP (see Supplementary Table 11) supplemented with lysozyme (1 mg ml^{-1}) and DNAase (0.01 U ml^{-1}) and subjected to sonication (10 mins, 50% amplitude, 1 s on/off pulse). Cell lysates were centrifuged at 10 000 g for 30 min. Supernatants were subjected to a Strep-Tactin Superflow Plus resin (Qiagen) and the purified protein was eluted using buffer NPD.

For purification of VioC MeHis316 for use in crystallography, the pelleted bacterial cells were suspended in lysis buffer (50 mM HEPES, 300 mM NaCl, 20 mM imidazole, pH 7.5) supplemented with lysozyme (1 mg ml^{-1}) and DNAase (0.01 U ml^{-1}) and subjected to sonication (10 mins, 50% amplitude, 1 s on/off pulse). Cell lysates were centrifuged at 10 000 g for 30 min. Supernatants were subjected to a nickel resin (Qiagen) and the purified protein was eluted using an elution buffer (50 mM HEPES, 300 mM NaCl, 250 mM imidazole, pH = 7.5).

After purification, proteins were desalted using 10DG desalting columns (Bio-Rad) into 100 mM Tris pH = 7.5 and stored for a maximum of 24 h at 4 °C before experimentation/crystallisation. Protein concentrations were determined by measuring the absorbance at 280 nm and assuming an extinction coefficient of 47 440 M^{-1} cm^{-1} for VioC and VioC MeHis316.

MS analysis

MS data for VioC and VioC MeHis were acquired on a 1200series LC (Agilent). The final protein concentrations were adjusted to 10 µM in 50 mM KPi pH 6. 5 µL of sample was injected into 1 ml min-1 5% acetonitrile (0.1% formic acid) and desalted inline. This was eluted over 1 minute by 95% acetonitrile. The resulting multiply charged spectrum was analysed by an

QTOF 6510 (Agilent) in ESI positive ion mode, and deconvoluted using Masshunter Software (Agilent). The instrument had previously been tuned and calibrated with reference solution. MS data is presented in Supplementary Table 1.

Crystallization, refinement and model building

His_6-tagged VioC MeHis316 was crystallized at 10 mg ml^{-1} in 20 mM HEPES buffer pH 7.5 containing 400 µM ammonium iron(II) sulphate hexahydrate, 2 mM L-arginine and 2 mM succinate. Crystallization conditions were identified using the JCSG-plus screen (Molecular Dimensions). Crystals suitable for diffraction experiments were obtained by sitting drop vapour diffusion in 400 nl drops containing equal volumes of protein and reservoir solution, after 72 h incubation at 4 °C. The reservoir solution comprised of 0.02 M magnesium chloride hexahydrate, 0.1 M HEPES, pH 7.5, containing 22 % (w/v) poly(acrylic acid sodium salt) 5100 as a precipitant. Prior to data collection crystals were cryoprotected with PEG 300 and plunge cooled in liquid nitrogen. All data were collected at Diamond Light Source (Harwell, UK). Data reduction was performed with Dials and the structure solved using a model derived from VioC structure 6ALQ. Iterative rounds of model building, and refinement were performed in COOT[1] and Phenix.refine[2] respectively. Validation with MOLPROBITY and PDBREDO[3] were incorporated into the iterative rebuild and refinement process. The resolution cut off was validated using a paired refinement in PDBREDO. Data collection and refinement statistics are shown in Supplementary Table 2.

Total turnover numbers

The total turnover numbers achieved by VioC and VioC MeHis316 were determined as follows. Reactions were performed in 1.5 mL microcentrifuge tubes (Eppendorf) containing a total volume of 100 µL in 100 mM Tris pH 7.5. A substrate mix was initiated by the addition of enzyme to give reactions with the final concentrations 1 µM enzyme, 5 mM 2-oxoglutarate (2OG), 1 mM L-arginine, 1 mM $(NH_4)_2Fe(SO_4)_2(H_2O)_6$, 1 mM sodium ascorbate. Reactions were incubated at 25 °C with shaking (300 rpm) for 18 h before quenching with 100 µL of 5 mM Fmoc *N*-hydroxysuccinimide ester in ACN. After a further 30 min shaking at 25 °C (300 rpm), the precipitated proteins were removed by centrifugation (14 000 g, 20 min) before high-pressure liquid chromatography (HPLC) analysis.

HPLC analysis

HPLC analysis of Fmoc-derived L-arginine and L-arginine-OH was performed on a 1290 Infinity II Agilent LC system with a Waters Acquity UPLC HSS column (2.1 x 100 mm, 1.8 µm). The column was equilibrated at 67% MPA (2 mM ammonium formate in water) and 33% MPB (90:10 v/v ACN:2 mM ammonium formate in ACN) with a flow rate of 0.2 mL min^{-1}. After injection of 2 µL of sample, it was held at 33% MPB for 6 min, followed by a one minute wash with 90% MPB. A one minute equilibration in 33% MPB was used to prepare the column for the next injection. Detection of L-arginine-Fmoc and L-arginine-Fmoc-OH was performed using absorption at 254 nm.

Stopped-flow kinetics

Stopped-flow absorbance experiments were performed inside a Belle Technology anaerobic chamber (oxygen levels < 5 ppm) using an SX20 rapid mixing stopped-flow spectrophotometer (Applied Photophysics Ltd, Leatherhead, UK) in single mixing configuration, equipped with a xenon arc lamp and a 1 cm path length in 100 mM Tris, pH 7.5 at 4 °C. Multiple wavelength data were using a photodiode array (PDA) detector and single wavelength data was obtained from a photomultiplier tube (PMT) single wavelength detector. All experiments were performed in 100 mM Tris pH 7.5 that had been degassed overnight before use. A 1 mL anaerobic substrate mix containing 18 mM 2OG, 18 mM L-arginine and 2.5 mM $(NH_4)_2Fe(SO_4)_2(H_2O)_6$ was prepared in 100 mM Tris pH 7.5, and subsequently re-adjusted to pH 7.5 using 28 µL 2M NaOH. The substrate mix was added slowly to VioC or VioC MeHis316 to give a final reaction mix containing 1 mM enzyme, 6 mM 2OG, 6 mM L-arginine, 1 mM $(NH_4)_2Fe(SO_4)_2(H_2O)_6$. After rapid mixing of the reactant complex with air-saturated 4 °C buffer, absorbance was monitored at 320 nm for kinetic parameter determination or across 200-800 nm for spectral characterisation of the intermediates for 100 s. Kinetic parameters were determined by fitting to a double exponential model (Equation 1) using OriginLab. The kinetic parameters are given in Supplementary Table S8 and the fits for each data set are shown in Supplementary Figure S6.

Equation 1

$$\Delta A_{320} = A_1 e^{-k_1 t} + A_2 \frac{k_1}{k_2 - k_1}\left(e^{-k_1 t} - e^{-k_2 t}\right)$$

DFT methods

Two QM cluster models were used to study the mechanism of VioC. The models A and B are shown in Supplementary Figures S2 and S3, respectively. In both models a crystal water that hydrogen bonds to the N-H of His316 was omitted in the VioC MeHis316 system.

Model A was used to probe the electron affinity of the ferryl intermediates in VioC and VioC MeHis316. The cluster model, in addition to the ferryl intermediate, contained the side chains of the residues (Me)His316, His168, Glu170, Arg334, Ser158, Asp286, Thr169, Val315, Asn311, Ile309, Gly166, succinate and arginine for a total of 240 atoms.

Model B was used to study the catalytic cycle of VioC and VioC MeHis316 and determine the energy landscapes for C3-hydroxylation of L-Arg. This larger cluster model includes additional residues in proximity to the substrate L-Arginine *c.f.* that shown in Supplementary Figure S2, giving a total of 353 atoms in this larger QM cluster. This model contains the ferryl intermediate bound to succinate, which was positioned *cis* to the peptide chain His168−Thr169−Glu170 and trans to the peptide chain His316−Val315−Ala314. In addition, several small peptide chains were included that position the substrate, namely, Thr155−Leu156−Val157−Ser158, Asp222−Asp223−Ser224, Asp268−Gly269−Asp270−Phe271, and Ile309−Asp310−Asn311. Arg334 side chain, Leu196 and two crystal water molecules were also included.

DFT calculations were conducted using Gaussian16 at the unrestricted B3LYP level of theory. Geometry optimizations and frequency calculations used the LANL2DZ basis set (with electron core potential) on the iron and 6-31G* on all other atoms. Local minima were characterized by a frequency calculation that confirmed them with real frequencies only. Free energies (G) were calculated at 298 K and 1 bar and include relative enthalpies with zero-point (ZPE), thermal and entropic corrections.

To improve the accuracy of the energetics, we ran single-point energy calculations using LACV3P+ on iron (with core potential) and 6-311+G* for the rest of the atoms (basis set BS2). These calculations also include dispersion contributions using the (GD3 method)[4] and solvent effects through the self-consistent reaction field approach. Chlorobenzene was selected as the solvent of choice and the conductor-like polarizable continuum model (CPCM) was used to apply a consistent perturbation to the SCF.[5] Free energies use thermal corrections and

entropies calculated at 298 K using the analytical frequency functionality as implemented in Gaussian09.[6]

DNA sequences

His$_6$-VioC

ATGGGCAGCAGCCATCATCATCATCATCACAGCAGCGGCCTGGTGCCGCGCGGCAGCCATATGGCT
AGCATGACTGGTGGACAGCAAATGGGTCGCGGATCCGAATTCATGACCGAAAGCCCGACCACCCAT
CATGGTGCAGCACCGCCTGATAGCGTTGCAACACCGGTTCGTCCGTGGTCAGAATTTCGTCTGACAC
CGGCAGAAGCAGCAGCAGCCGCAGCACTGGCAGCACGTTGTGCACAGCGTTATGATGAAACCGAT
GGTCCGGAATTTCTGCTGGATGCACCGGTTATTGCACATGAACTGCCTCGTCGTCTGCGTACCTTTAT
GGCACGTGCGCGTCTGGATGCATGGCCTCATGCACTGGTTGTTCGTGGTAATCCGGTTGATGATGCA
GCCCTGGGTAGCACTCCGGTTCATTGGCGTACCGCACGTACACCGGGTAGCCGTCCGCTGAGCTTCC
TGCTGATGCTGTATGCAGGTCTGCTGGGTGATGTTTTTGGTTGGGCAACCCAGCAGGATGGTCGTGT
TGTTACCGATGTTCTGCCGATTAAAGGTGGTGAACATACCCTGGTTAGCAGCAGCTCACGTCAAGAA
CTGGGTTGGCATACCGAAGATGCATTTAGCCCGTATCGTGCAGATTATGTTGGTCTGCTGAGCCTGC
GTAATCCGGATGGTGTTGCCACCACCCTGGCAGGCGTTCCGCTGGATGATCTGGATGAACGCACCCT
GGATGTTCTGTTTCAAGAACGTTTTCTGATTCGTCCGGATGATAGCCATCTGCAGGTTAATAACAGCA
CCGCACAGCAGGGTCGTGTGGAATTTGAAGGTATTGCACAGGCAGCAGATCGTCCGGAACCGGTTG
CCATTCTGACAGGTCATCGTGCCGCACCGCATCTGCGTGTTGATGGTGATTTTTCAGCCCCTGCCGAA
GGTGATGAAGAAGCCGCAGCGGCACTGGGCACCCTGCGTAAACTGATTGATGCAAGCCTGTATGAA
CTGGTTCTGGATCAGGGTGATGTGGCCTTTATTGATAATCGTCGTGCCGTTCATGGTCGTCGTGCATT
TCAGCCTCGCTATGATGGTCGCGATCGTTGGCTGAAACGTATTAACATTACCCGTGATCTGCATCGTA
GCCGTAAAGCATGGGCAGGCGATAGCCGTGTTCTGGGTCAGCGTTAA

His$_6$-VioC MeHis

ATGGGCAGCAGCCATCATCATCATCATCACAGCAGCGGCCTGGTGCCGCGCGGCAGCCATATGGCT
AGCATGACTGGTGGACAGCAAATGGGTCGCGGATCCGAATTCATGACCGAAAGCCCGACCACCCAT
CATGGTGCAGCACCGCCTGATAGCGTTGCAACACCGGTTCGTCCGTGGTCAGAATTTCGTCTGACAC
CGGCAGAAGCAGCAGCAGCCGCAGCACTGGCAGCACGTTGTGCACAGCGTTATGATGAAACCGAT
GGTCCGGAATTTCTGCTGGATGCACCGGTTATTGCACATGAACTGCCTCGTCGTCTGCGTACCTTTAT
GGCACGTGCGCGTCTGGATGCATGGCCTCATGCACTGGTTGTTCGTGGTAATCCGGTTGATGATGCA
GCCCTGGGTAGCACTCCGGTTCATTGGCGTACCGCACGTACACCGGGTAGCCGTCCGCTGAGCTTCC
TGCTGATGCTGTATGCAGGTCTGCTGGGTGATGTTTTTGGTTGGGCAACCCAGCAGGATGGTCGTGT
TGTTACCGATGTTCTGCCGATTAAAGGTGGTGAACATACCCTGGTTAGCAGCAGCTCACGTCAAGAA
CTGGGTTGGCATACCGAAGATGCATTTAGCCCGTATCGTGCAGATTATGTTGGTCTGCTGAGCCTGC
GTAATCCGGATGGTGTTGCCACCACCCTGGCAGGCGTTCCGCTGGATGATCTGGATGAACGCACCCT
GGATGTTCTGTTTCAAGAACGTTTTCTGATTCGTCCGGATGATAGCCATCTGCAGGTTAATAACAGCA
CCGCACAGCAGGGTCGTGTGGAATTTGAAGGTATTGCACAGGCAGCAGATCGTCCGGAACCGGTTG
CCATTCTGACAGGTCATCGTGCCGCACCGCATCTGCGTGTTGATGGTGATTTTTCAGCCCCTGCCGAA
GGTGATGAAGAAGCCGCAGCGGCACTGGGCACCCTGCGTAAACTGATTGATGCAAGCCTGTATGAA
CTGGTTCTGGATCAGGGTGATGTGGCCTTTATTGATAATCGTCGTGCCGTTTAGGGTCGTCGTGCAT
TTCAGCCTCGCTATGATGGTCGCGATCGTTGGCTGAAACGTATTAACATTACCCGTGATCTGCATCGT
AGCCGTAAAGCATGGGCAGGCGATAGCCGTGTTCTGGGTCAGCGTTAA

StrepII-VioC

ATGGGCAGCAGCTGGAGTCACCCACAGTTTGAGAAAGGCAGCCATATGGCTAGCATGACTGGTGGA
CAGCAAATGGGTCGCGGATCCGAATTCATGACCGAAAGCCCGACCACCCATCATGGTGCAGCACCG
CCTGATAGCGTTGCAACACCGGTTCGTCCGTGGTCAGAATTTCGTCTGACACCGGCAGAAGCAGCA

GCAGCCGCAGCACTGGCAGCACGTTGTGCACAGCGTTATGATGAAACCGATGGTCCGGAATTTCTG
CTGGATGCACCGGTTATTGCACATGAACTGCCTCGTCGTCTGCGTACCTTTATGGCACGTGCGCGTCT
GGATGCATGGCCTCATGCACTGGTTGTTCGTGGTAATCCGGTTGATGATGCAGCCCTGGGTAGCACT
CCGGTTCATTGGCGTACCGCACGTACACCGGGTAGCCGTCCGCTGAGCTTCCTGCTGATGCTGTATG
CAGGTCTGCTGGGTGATGTTTTTGGTTGGGCAACCCAGCAGGATGGTCGTGTTGTTACCGATGTTCT
GCCGATTAAAGGTGGTGAACATACCCTGGTTAGCAGCAGCTCACGTCAAGAACTGGGTTGGCATAC
CGAAGATGCATTTAGCCCGTATCGTGCAGATTATGTTGGTCTGCTGAGCCTGCGTAATCCGGATGGT
GTTGCCACCACCCTGGCAGGCGTTCCGCTGGATGATCTGGATGAACGCACCCTGGATGTTCTGTTTC
AAGAACGTTTTCTGATTCGTCCGGATGATAGCCATCTGCAGGTTAATAACAGCACCGCACAGCAGGG
TCGTGTGGAATTTGAAGGTATTGCACAGGCAGCAGATCGTCCGGAACCGGTTGCCATTCTGACAGG
TCATCGTGCCGCACCGCATCTGCGTGTTGATGGTGATTTTTCAGCCCCTGCCGAAGGTGATGAAGAA
GCCGCAGCGGCACTGGGCACCCTGCGTAAACTGATTGATGCAAGCCTGTATGAACTGGTTCTGGAT
CAGGGTGATGTGGCCTTTATTGATAATCGTCGTGCCGTTCATGGTCGTCGTGCATTTCAGCCTCGCTA
TGATGGTCGCGATCGTTGGCTGAAACGTATTAACATTACCCGTGATCTGCATCGTAGCCGTAAAGCA
TGGGCAGGCGATAGCCGTGTTCTGGGTCAGCGTTAA

StrepII-VioC MeHis

ATGGGCAGCAGCTGGAGTCACCCACAGTTTGAGAAAGGCAGCCATATGGCTAGCATGACTGGTGGA
CAGCAAATGGGTCGCGGATCCGAATTCATGACCGAAAGCCCGACCACCCATCATGGTGCAGCACCG
CCTGATAGCGTTGCAACACCGGTTCGTCCGTGGTCAGAATTTCGTCTGACACCGGCAGAAGCAGCA
GCAGCCGCAGCACTGGCAGCACGTTGTGCACAGCGTTATGATGAAACCGATGGTCCGGAATTTCTG
CTGGATGCACCGGTTATTGCACATGAACTGCCTCGTCGTCTGCGTACCTTTATGGCACGTGCGCGTCT
GGATGCATGGCCTCATGCACTGGTTGTTCGTGGTAATCCGGTTGATGATGCAGCCCTGGGTAGCACT
CCGGTTCATTGGCGTACCGCACGTACACCGGGTAGCCGTCCGCTGAGCTTCCTGCTGATGCTGTATG
CAGGTCTGCTGGGTGATGTTTTTGGTTGGGCAACCCAGCAGGATGGTCGTGTTGTTACCGATGTTCT
GCCGATTAAAGGTGGTGAACATACCCTGGTTAGCAGCAGCTCACGTCAAGAACTGGGTTGGCATAC
CGAAGATGCATTTAGCCCGTATCGTGCAGATTATGTTGGTCTGCTGAGCCTGCGTAATCCGGATGGT
GTTGCCACCACCCTGGCAGGCGTTCCGCTGGATGATCTGGATGAACGCACCCTGGATGTTCTGTTTC
AAGAACGTTTTCTGATTCGTCCGGATGATAGCCATCTGCAGGTTAATAACAGCACCGCACAGCAGGG
TCGTGTGGAATTTGAAGGTATTGCACAGGCAGCAGATCGTCCGGAACCGGTTGCCATTCTGACAGG
TCATCGTGCCGCACCGCATCTGCGTGTTGATGGTGATTTTTCAGCCCCTGCCGAAGGTGATGAAGAA
GCCGCAGCGGCACTGGGCACCCTGCGTAAACTGATTGATGCAAGCCTGTATGAACTGGTTCTGGAT
CAGGGTGATGTGGCCTTTATTGATAATCGTCGTGCCGTTTAGGGTCGTCGTGCATTTCAGCCTCGCT
ATGATGGTCGCGATCGTTGGCTGAAACGTATTAACATTACCCGTGATCTGCATCGTAGCCGTAAAGC
ATGGGCAGGCGATAGCCGTGTTCTGGGTCAGCGTTAA

Protein sequences

His$_6$-VioC
MGSSHHHHHHSSGLVPRGSHMASMTGGQQMGRGSEFMTESPTTHHGAAPPDSVATPVRPWSEFRL
TPAEAAAAAALAARCAQRYDETDGPEFLLDAPVIAHELPRRLRTFMARARLDAWPHALVVRGNPVDDA
ALGSTPVHWRTARTPGSRPLSFLLMLYAGLLGDVFGWATQQDGRVVTDVLPIKGGEHTLVSSSSRQELG
WHTEDAFSPYRADYVGLLSLRNPDGVATTLAGVPLDDLDERTLDVLFQERFLIRPDDSHLQVNNSTAQQ
GRVEFEGIAQAADRPEPVAILTGHRAAPHLRVDGDFSAPAEGDEEAAAALGTLRKLIDASLYELVLDQGD
VAFIDNRRAVHGRRAFQPRYDGRDRWLKRINITRDLHRSRKAWAGDSRVLGQR-

His$_6$-VioC MeHis316
MGSSHHHHHHSSGLVPRGSHMASMTGGQQMGRGSEFMTESPTTHHGAAPPDSVATPVRPWSEFRL
TPAEAAAAAALAARCAQRYDETDGPEFLLDAPVIAHELPRRLRTFMARARLDAWPHALVVRGNPVDDA
ALGSTPVHWRTARTPGSRPLSFLLMLYAGLLGDVFGWATQQDGRVVTDVLPIKGGEHTLVSSSSRQELG
WHTEDAFSPYRADYVGLLSLRNPDGVATTLAGVPLDDLDERTLDVLFQERFLIRPDDSHLQVNNSTAQQ
GRVEFEGIAQAADRPEPVAILTGHRAAPHLRVDGDFSAPAEGDEEAAAALGTLRKLIDASLYELVLDQGD
VAFIDNRRAV(MeHis)GRRAFQPRYDGRDRWLKRINITRDLHRSRKAWAGDSRVLGQR-

StrepII-VioC
MGSSWSHPQFEKGSHMASMTGGQQMGRGSEFMTESPTTHHGAAPPDSVATPVRPWSEFRLTPAEA
AAAAALAARCAQRYDETDGPEFLLDAPVIAHELPRRLRTFMARARLDAWPHALVVRGNPVDDAALGST
PVHWRTARTPGSRPLSFLLMLYAGLLGDVFGWATQQDGRVVTDVLPIKGGEHTLVSSSSRQELGWHTE
DAFSPYRADYVGLLSLRNPDGVATTLAGVPLDDLDERTLDVLFQERFLIRPDDSHLQVNNSTAQQGRVEF
EGIAQAADRPEPVAILTGHRAAPHLRVDGDFSAPAEGDEEAAAALGTLRKLIDASLYELVLDQGDVAFID
NRRAVHGRRAFQPRYDGRDRWLKRINITRDLHRSRKAWAGDSRVLGQR-

StrepII-VioC MeHis316
MGSSWSHPQFEKGSHMASMTGGQQMGRGSEFMTESPTTHHGAAPPDSVATPVRPWSEFRLTPAEA
AAAAALAARCAQRYDETDGPEFLLDAPVIAHELPRRLRTFMARARLDAWPHALVVRGNPVDDAALGST
PVHWRTARTPGSRPLSFLLMLYAGLLGDVFGWATQQDGRVVTDVLPIKGGEHTLVSSSSRQELGWHTE
DAFSPYRADYVGLLSLRNPDGVATTLAGVPLDDLDERTLDVLFQERFLIRPDDSHLQVNNSTAQQGRVEF
EGIAQAADRPEPVAILTGHRAAPHLRVDGDFSAPAEGDEEAAAALGTLRKLIDASLYELVLDQGDVAFID
NRRAV(MeHis)GRRAFQPRYDGRDRWLKRINITRDLHRSRKAWAGDSRVLGQR-

Supplementary Tables

Supplementary Table S1. Protein mass spectrometry

Protein	Calculated (Da)	Observed (Da)
StrepII-VioC	42636.7	42636.0
StrepII-VioC MeHis316	42650.7	42649.0

Supplementary Table S2. Data collection and refinement statistics for structure determination of His_6-VioC MeHis316

	**VioC MeHis316**
PDB ID	9EQF
Wavelength (Å)	0.976
Resolution range	38.48 - 1.6 (1.64 - 1.6)
Space group	C 1 2 1
Unit cell dimensions<br>a, b, c, (Å)<br>α, β, γ (°)	 81.1 67.1 63.0<br>90 109.2 90
Total reflections	278892 (14180)
Unique reflections	41962 (2681)
Multiplicity	6.6 (5.3)
Completeness (%)	99.61 (96.09)
Mean I/sigma(I)	15.47 (1.86)
Wilson B-factor (Å^2)	17.87
R-merge	0.05875 (0.4329)
R-meas	0.06364 (0.4804)
R-pim	0.02425 (0.2041)
$CC_{1/2}$	0.999 (0.922)
CC*	1 (0.98)
Reflections used in refinement	41951 (2678)
Reflections used for R-free	2082 (110)
R-work	0.1573 (0.2354)
R-free	0.1865 (0.2619)
Protein residues	336
RMS(bonds)	0.003
RMS(angles)	0.74
Ramachandran favoured (%)	97.56
Ramachandran allowed (%)	1.83
Ramachandran outliers (%)	0.61
Rotamer outliers (%)	1.4
Clashscore	3.95
Average B-factor (Å^2)	26.3

Supplementary Table S3. Absolute energies, zero-point energies and free energies (in a.u.), and relative energies (in kcal mol^{-1}) of the optimized geometries for the Fe(IV) and Fe(III) complexes of as obtained in Gaussian-16 from model A. All calculations were done in the gas phase.

	E [a.u.]	ZPE [a.u.]	G [a.u.]	Relative to Fe(IV) [kcal mol^{-1}]
WT Fe(IV)	-5633.509411	2.058883	-5631.651381	0.00
WT Fe(III)	-5633.642732	2.055663	-5631.786492	-85.68
MeHis Fe(IV)	-5596.379568	2.060985	-5594.518547	0.00
MeHis Fe(III)	-5596.514536	2.058215	-5594.661565	-86.43

Supplementary Table S4. Group Mulliken spin-densities for both wildtype (WT) VioC and VioC MeHis316 model optimized geometries for the Fe(IV) and Fe(III) complexes from model A, calculated at UB3LYP/6-31G* level of theory.

	Fe	O	Succinate	Arg sub	Glu_{170}	$(Me)His_{316}$	His_{168}
WT Fe(IV)	3.039	0.643	0.167	0.005	0.101	-0.004	0.045
WT Fe(III)	3.845	0.832	0.113	0.011	0.088	0.037	0.068
MeHis Fe(IV)	3.036	0.643	0.167	0.005	0.103	-0.004	0.046
MeHis Fe(III)	3.873	0.840	0.115	0.006	0.075	0.027	0.061

Supplementary Table S5. Absolute energies (in a.u.) and relative energies (in kcal mol^{-1}) of single point geometries for Fe(IV) and Fe(III) models to assess the adiabatic electron affinity of VioC and VioC MeHis316 in model A. Energies were calculated for models of VioC, VioC MeHis316, and the wildtype without the ordered water molecule H-bonding to His316 N_{δ} were calculated in Gaussian-16. All calculations were done in the gas phase.

	E [a.u.]	Relative to Fe(IV) [kcal mol^{-1}]
WT Fe(IV)	-5633.509411	0.00
WT Fe(III)	-5633.598648	-56.00
MeHis Fe(IV)	-5596.379568	0.00
MeHis Fe(III)	-5596.470848	-57.28
WT Fe(IV) – no H_2O	-5557.056319	0.00
WT Fe(III) – no H_2O	-5557.151635	-59.81

Supplementary Table S6. Group Mulliken spin-densities for both wildtype (WT) VioC and VioC MeHis316 optimized geometries, calculated at UB3LYP/BS1.

	Fe	O	Succ	Arg(OH)	GLU_{170}	$(Me)HIS_{316}$	HIS_{168}	H_2O_{CRY}	Prot
$^5Re_{WT}$	3.19	0.65	0.13	0.00	0.10	-0.11	0.04	0.00	0.00
$^5Re_{MeHis}$	3.19	0.69	0.18	0.00	0.05	-0.11	0.01	-0.01	-0.01
$^5TS_{1,WT}$	4.12	-0.09	0.22	-0.36	0.07	0.03	0.01	0.00	0.00
$^5TS_{1,MeHis}$	4.12	-0.07	0.22	-0.36	0.06	0.02	0.02	0.00	-0.01
$^5I_{WT}$	4.33	0.23	0.26	-0.96	0.12	0.03	0.00	0.00	0.00
$^5I_{MeHis}$	4.34	0.26	0.25	-0.99	0.11	0.01	0.01	0.00	0.00
$^5TS_{2,WT}$	4.26	0.25	0.22	-0.85	0.09	0.03	0.01	0.00	-0.01
$^5TS_{2,MeHis}$	4.28	0.26	0.22	-0.86	0.09	0.01	0.02	0.00	-0.01
$^5P_{WT}$	3.83	-	0.09	0.00	0.03	0.02	0.02	0.00	0.00
$^5P_{MeHis}$	4.34	-	0.25	-0.73	0.11	0.01	0.01	0.00	0.00

Supplementary Table S7. Calculated Kinetic Isotope Effects (KIEs) for C-H abstraction by the ferryl intermediates in VioC and VioCMeHis316 and the imaginary frequencies for the transition states of C-H/D abstraction. Eyring primary (**KIE^a**) kinetic isotope effects for HAT as well as the KIEs in perdeuterated substate (**KIE^b**) were calculated for both VioC and VioC MeHis316 models. Values in brackets are the KIEs including a Wigner correction using the imaginary frequencies of the transition state barriers to C-H and C-D abstraction.[7]

	**VioC**	**VioC MeHis316**
KIE^a	6.67 (8.89)	6.48 (8.53)
KIE^b	7.87 (10.49)	7.80 (10.27)
Frequency $TS_{(C-H)}$ (cm^{-1})	i1182.6	i1154.0
Frequency $TS_{(C-D)}$ (cm^{-1})	i909.5	i892.9

Supplementary Table S8. Kinetic parameters for ferryl formation and decay from stopped-flow UV-vis measurement. Values and standard errors are presented from triplicate measurements.

	VioC		VioC MeHis316	
	h_7-L-Arg	d_7-L-Arg	h_7-L-Arg	d_7-L-Arg
$k_{form,obvs}$	41.2 ± 0.7	29.6 ± 1.0	46.8 ± 1.0	24.9 ± 0.5
k_H or k_D	11.9 ± 0.1	0.28 ± 0.01	8.3 ± 0.1	0.29 ± 0.01
ΔA_1	0.070 ± 0.002	0.075 ± 0.002	0.030 ± 0.001	0.035 ± 0.001
ΔA_2	0.30 ± 0.01	0.34 ± 0.01	0.20 ± 0.01	0.26 ± 0.01

Supplementary Table S9. Observed kinetic isotope effects. Kinetic isotope effects are given for the formation ($k_{form,obvs}$) and decay (k) of the ferryl intermediate with errors propagated from triplicate measurements of $k_{1,obvs}$(H), $k_{1,obvs}$(D), k_2(H), and k_2(D).

	VioC	VioC MeHis316
$k_{form,obvs}$(H)/ $k_{form,obvs}$(D)	1.4 ± 0.1	1.9 ± 0.1
k_H/k_D	43 ± 1	29 ± 1

Supplementary Table S10. Primers used in this study

Primer Name	Sequence
VioC_fwd	CGCGGATCCGAATTC
VioC_rev	GTGGTGGTGCTCG
H316TAG_fwd	AATCGTCGTGCCGTTTAGGGTCGTCGTGCATTT
H316_rev	AACGGCACGACGA
VioC_fwd2	GGCAGCCATATGGCT
VioC_rev2	GCTGCTGCCCATGG
Strep_A	ACCATGGGCAGCAGCTGGAGTCACCCACAGTTTGAGAAAGGCAGC CATATGGCT
Strep_B	AGCCATATGGCTGCCTTTCTCAAACTGTGGGTGACTCCAGCTGCT GCCCATGGT

Supplementary Table S11. Buffers used in this study

Buffer	Components	pH
NP	50 mM NaH_2PO_4, 300 mM NaCl, adjust pH with NaOH	8
NPD	50 mM NaH_2PO_4, 300 mM NaCl, 2.5 mM desthiobiotin, adjust pH with NaOH	8
Lysis buffer	50 mM HEPES, 300 mM NaCl, 20 mM imidazole, adjust pH with HCl	7.5
Elution buffer	50 mM HEPES, 300 mM NaCl, 250 mM imidazole, adjust pH with HCl	7.5
Reaction buffer	100 mM Tris	7.5

Supplementary Figures

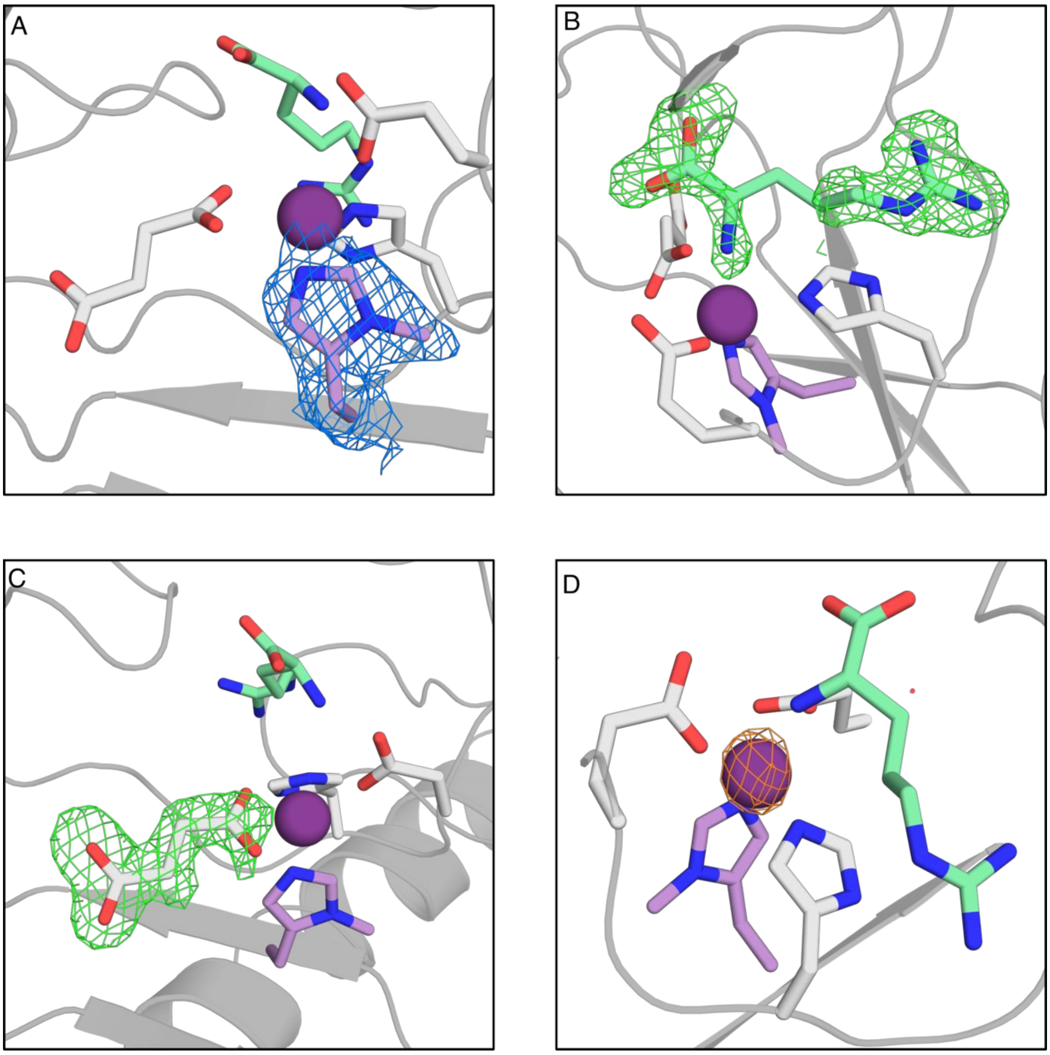

Supplementary Figure S1. Structural characterization of VioC MeHis316. Active site residues, MeHis316 and the substrate L-Arg and are shown as atom-coloured sticks, with purple, grey, and green carbons, respectively. The iron cofactor is shown as a purple sphere. A) A $2F_o$-F_c map is shown around the MeHis, contoured at 1 σ. B) An F_o-F_c omit map is shown around the substrate L-Arg, contoured at 2.5 σ. C) An F_o-F_c omit map is shown around succinate, contoured at 2.5 σ. D) An anomalous map is shown around the Fe, contoured at 3σ.

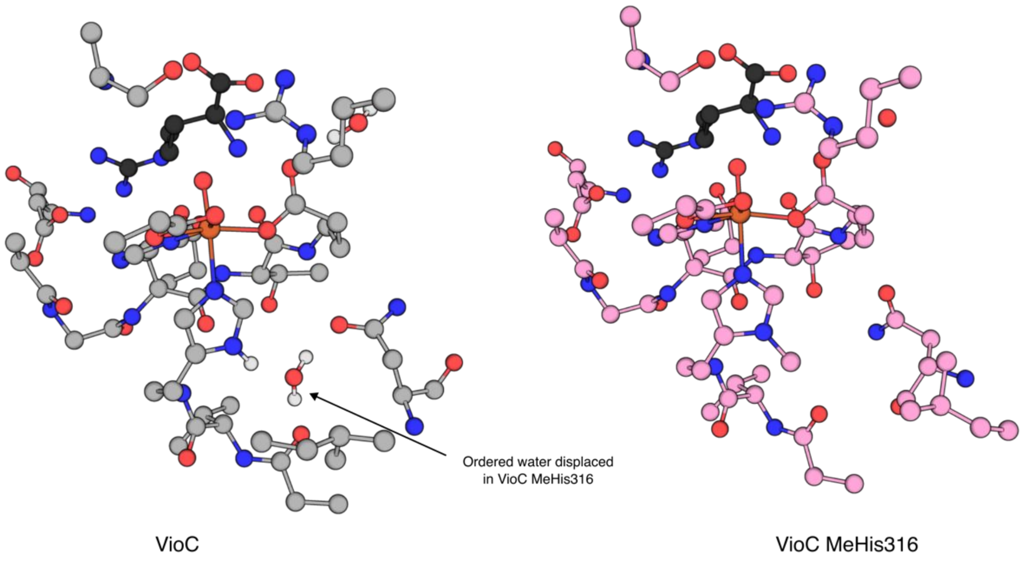

Supplementary Figure S2. QM cluster model A. A QM cluster model was generated to probe the electron affinity of the ferryl intermediates in VioC and VioC MeHis316. The cluster model, in addition to the ferryl intermediate, contained the side chains of the residues (Me)His316, His168, Glu170, Arg334, Ser158, Asp286, Thr169, Val315, Asn311, Ile309, Gly166, succinate and arginine for a total of 240 atoms.

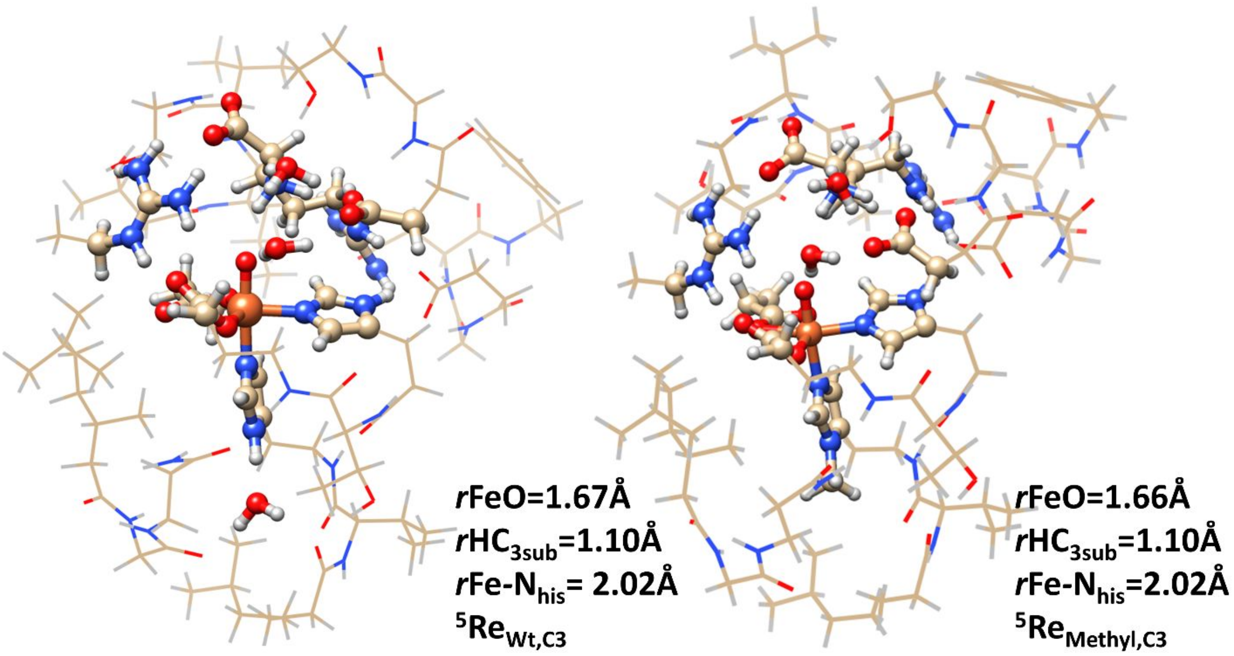

Supplementary Figure S3. QM cluster model B. A QM cluster model was generated to probe the energy landscapes for L-Arg hydroxylation. Optimised geometries of DFT models of the ferryl states in VioC and VioC MeHis316 are shown. This larger cluster model includes additional residues in proximity to the substrate L-Arginine *c.f.* that shown in Supplementary Figure S2, giving a total of 353 atoms in this larger QM cluster. This model contains the ferryl intermediate bound to succinate, which was positioned *cis* to the peptide chain His168−Thr169−Glu170 and trans to the peptide chain His316−Val315−Ala314. In addition, several small peptide chains were included that position the substrate, namely,

Thr155−Leu156−Val157−Ser158, Asp222−Asp223−Ser224, Asp268−Gly269−Asp270−Phe271, and Ile309−Asp310−Asn311. Arg334 side chain, Leu196 and two crystal water molecules were also included.

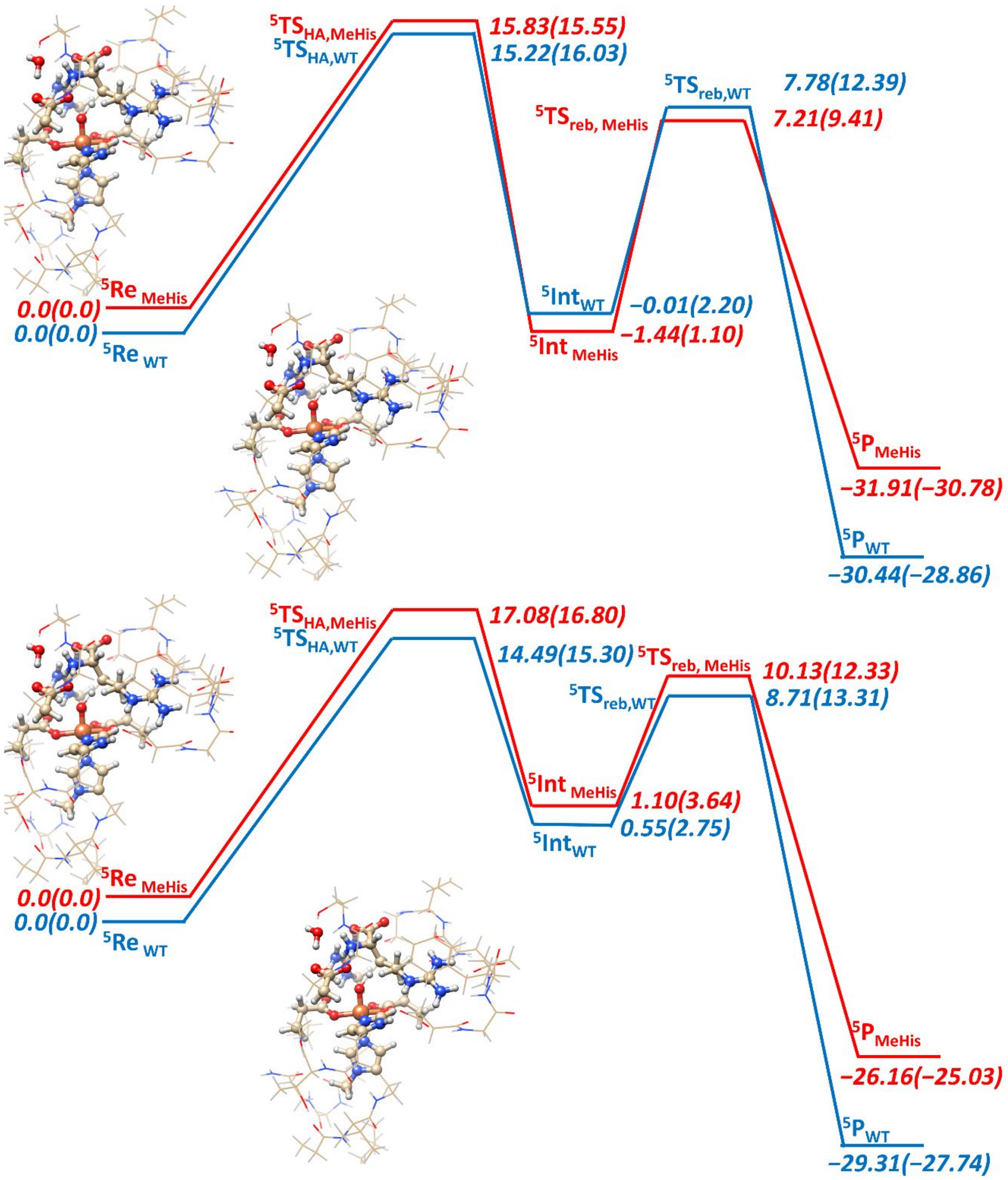

Supplementary Figure S4. Calculated reaction landscapes for WT VioC and VioC MeHis316 cluster models. (upper) Energies given as both zero-point (ΔE+ZPE) energies and free energies in parenthesis. All energies given in kcal mol^{-1} as calculated at UB3LYP/BS2 level of theory. (upper) energies calculated in gas phase, (lower) solvent corrected energies.

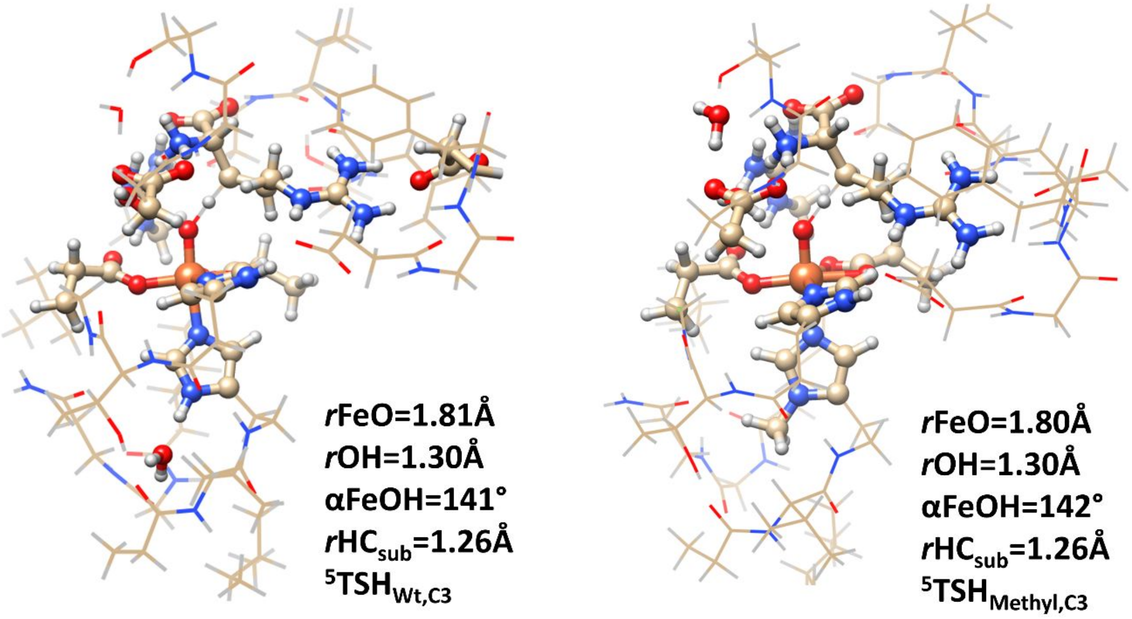

Supplementary Figure S5. Structure and important bonding information for the hydrogen atom abstraction transition state structures of (left) VioC and (right) VioC MeHis316.

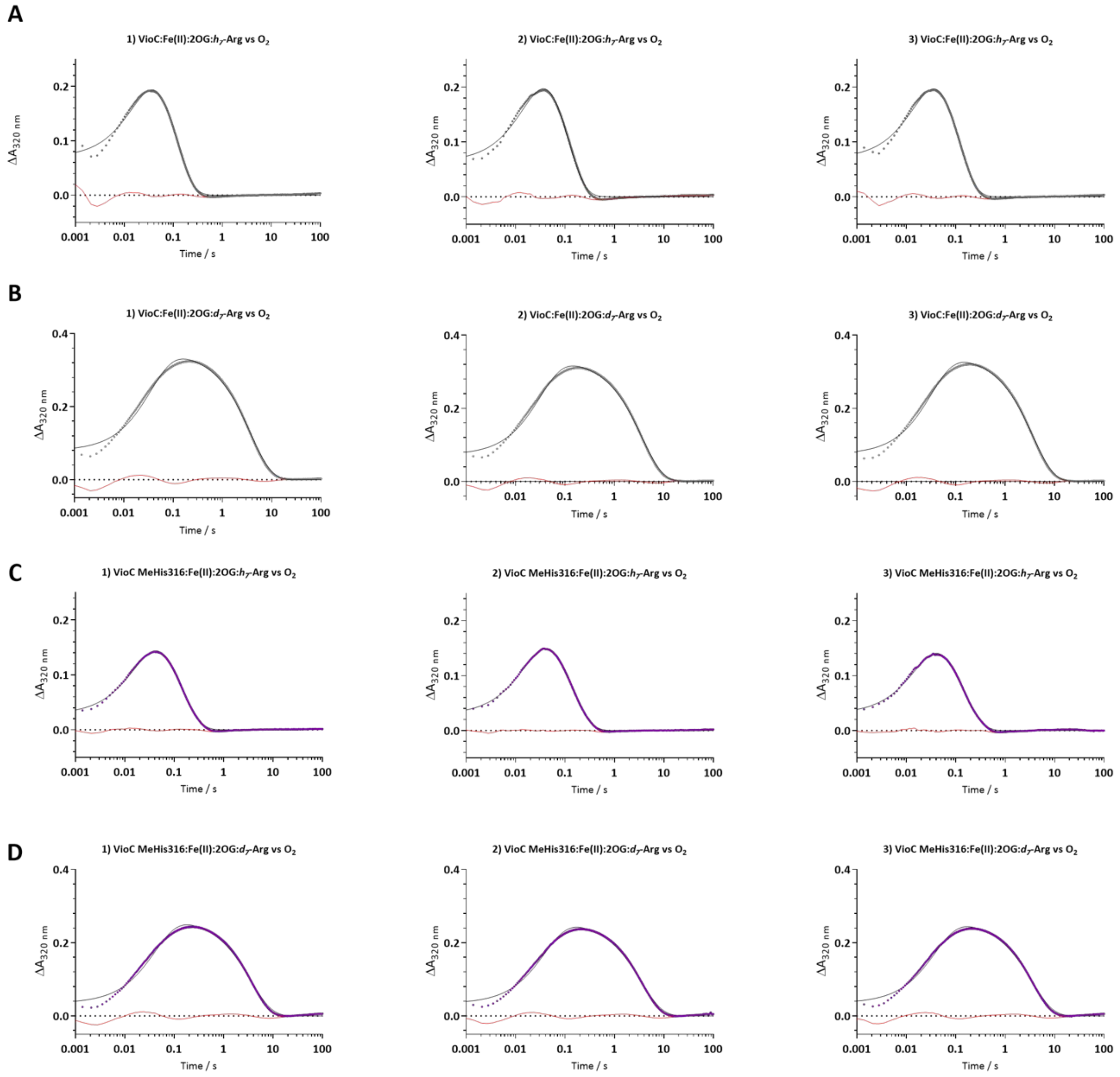

Supplementary Figure S6. Fits of stopped-flow data to kinetic models. Time dependent absorbance changes at 320 nm are shown after rapid mixing an anoxic reactant complex VioC/VioC MeHis316:h_7-Arg/d_7-Arg:2OG:Fe(II) with aerobic buffer. Data points are shown as dots and the fitted models overlaid as black lines. The residuals from fitting the model to the data are shown for each measurement as a red line. Final concentration after mixing at 5 °C with air-saturated buffer are 500 µM enzyme, 3 mM 2OG, 3 mM h_7-Arg or per-d_7-Arg, and 500 µM $(NH_4)_2Fe(SO_4)_2(H_2O)_6$. A1-3) The formation and decay of the ferryl intermediate of VioC in reaction with h_7-Arg. B1-3) The formation and decay of the ferryl intermediate of VioC in reaction with d_7-Arg. C1-3) The formation and decay of the ferryl intermediate of VioC MeHis316 in reaction with h_7-Arg. D1-3) The formation and decay of the ferryl intermediate of VioC MeHis316 in reaction with d_7-Arg.

Supplementary References

(1) Emsley, P.; Cowtan, K. Coot: model-building tools for molecular graphics. *Acta Crystallographica Section D* **2004**, *60* (12), 2126-2132.
(2) Liebschner, D.; Afonine, P. V.; Baker, M. L.; Bunkoczi, G.; Chen, V. B.; Croll, T. I.; Hintze, B.; Hung, L.-W.; Jain, S.; McCoy, A. J.; et al. Macromolecular structure determination using X-rays, neutrons and electrons: recent developments in Phenix. *Acta Crystallographica Section D* **2019**, *75* (10), 861-877.
(3) Joosten, R. P.; Long, F.; Murshudov, G. N.; Perrakis, A. The PDB_REDO server for macromolecular structure model optimization. *IUCrJ* **2014**, *1* (4), 213-220.
(4) Grimme, S.; Antony, J.; Ehrlich, S.; Krieg, H. A consistent and accurate ab initio parametrization of density functional dispersion correction (DFT-D) for the 94 elements H-Pu. *The Journal of chemical physics* **2010**, *132*, 154104-154104.
(5) Tomasi, J.; Mennucci, B.; Cammi, R. Quantum Mechanical Continuum Solvation Models. *Chemical Reviews* **2005**, *105* (8), 2999-3094. DOI: 10.1021/cr9904009.
(6) Frisch, M. J.; Trucks, G. W.; Schlegel, H. B.; Scuseria, G. E.; Robb, M. A.; Cheeseman, J. R.; G. Scalmani, V. B.; Mennucci, B.; Petersson, G. A.; Nakatsuji, H.; et al. Gaussian 09, Revis. B.01, Gaussian, Inc., Wallingford CT, 200.
(7) Bell, R. P. *The Tunnel Effect in Chemistry*; Springer New York, NY, 1980. DOI: https://doi.org/10.1007/978-1-4899-2891-7.

Cartesian coordinates for model A

VioC MeHis316 Fe(IV) complex

6	5.698220000	13.679212000	21.793767000
6	4.452427000	12.908373000	21.379226000
8	4.287879000	11.689472000	21.606623000
1	6.105235000	14.156297000	20.888423000
1	5.379125000	14.505548000	22.442311000
7	3.051301000	11.230946000	18.830421000
6	3.859856000	10.888553000	17.669985000
6	3.513112000	9.542389000	16.993987000
8	2.843249000	8.650888000	17.532539000
6	5.370440000	10.925217000	18.036844000
6	5.830760000	9.779940000	18.886504000
6	6.248521000	8.523615000	18.519528000
7	5.907351000	9.797027000	20.271063000
6	6.359548000	8.596399000	20.689809000
7	6.566653000	7.800619000	19.647622000
1	3.686987000	11.678791000	16.935450000
1	5.573565000	11.886688000	18.523406000
1	5.933878000	10.926139000	17.101862000
1	3.402266000	10.934499000	19.737070000
1	6.317111000	8.112222000	17.523840000
1	6.553105000	8.337817000	21.718019000
7	4.058398000	9.438517000	15.758360000
6	3.975208000	8.257751000	14.918157000
6	5.266480000	7.403421000	14.973014000
8	6.360872000	7.854107000	15.356649000
6	3.540745000	8.711563000	13.481009000
6	3.995410000	7.866442000	12.297639000
8	2.103833000	8.751269000	13.430392000
1	3.173026000	7.628313000	15.314059000
1	3.937198000	9.723989000	13.340246000
1	3.601083000	8.320324000	11.383488000
1	5.087353000	7.852371000	12.230113000
1	3.635623000	6.836109000	12.353090000
1	1.801358000	9.448972000	14.034843000
1	4.593306000	10.247600000	15.370873000
7	5.061238000	6.130161000	14.589532000
6	6.056925000	5.074752000	14.660525000
6	5.577656000	3.928497000	15.577803000
6	6.732159000	3.203092000	16.295754000
6	7.519231000	4.211859000	17.135169000
8	6.854568000	4.677519000	18.146077000
8	8.653884000	4.570189000	16.778513000
1	6.974463000	5.525354000	15.040433000
1	4.976480000	3.215942000	15.002849000
1	4.917071000	4.361113000	16.336096000
1	7.414236000	2.725415000	15.586104000
1	6.322603000	2.424880000	16.949896000
1	4.126721000	5.838098000	14.291863000
6	2.859469000	13.989607000	14.592501000
6	3.417735000	12.691982000	13.983357000
6	4.943394000	12.611965000	14.149714000
8	5.467680000	11.573189000	14.689687000
8	5.600593000	13.609025000	13.767538000
7	5.309288000	17.399742000	15.642584000
6	5.715740000	18.183082000	16.811767000
6	6.425004000	19.440051000	16.326025000
8	7.289798000	20.036594000	16.931066000
6	6.516935000	17.452894000	17.915467000
6	5.788713000	16.280924000	18.638596000
8	4.657477000	16.470827000	19.125219000
8	6.440237000	15.169937000	18.697759000
1	4.782352000	18.543264000	17.275353000
1	6.787195000	18.195397000	18.677069000
1	7.460467000	17.087215000	17.493265000
1	6.094732000	16.865730000	15.273019000
6	1.147658000	-0.595784000	18.682522000
6	0.821400000	-0.632537000	17.182124000
6	0.226608000	-2.000345000	16.789062000
6	2.065975000	-0.274725000	16.352968000
6	-0.372323000	-2.064392000	15.379155000
1	1.905666000	-1.348391000	18.936346000
1	0.050370000	0.132881000	16.991676000
1	-0.554924000	-2.263559000	17.514981000
1	1.007350000	-2.768305000	16.891772000
1	2.857297000	-1.021137000	16.502318000
1	1.857735000	-0.223468000	15.278635000
1	2.471457000	0.698772000	16.656962000
1	-1.187729000	-1.336591000	15.265857000
1	0.372825000	-1.862038000	14.601632000
1	-0.794741000	-3.054833000	15.175989000
7	0.125115000	1.251171000	13.501739000
6	0.435726000	2.622906000	13.939431000
6	-0.822982000	3.232005000	14.551999000
8	-1.503168000	4.087770000	14.018435000
6	0.966170000	3.448247000	12.759592000
6	1.603464000	4.766781000	13.196962000
7	0.839392000	5.871690000	13.094664000
8	2.775750000	4.778697000	13.600067000
6	-3.230231000	5.060051000	17.793849000
6	-1.827161000	5.644988000	17.639123000
8	-0.832067000	4.952212000	17.424180000
7	-1.775332000	7.008156000	17.763524000
6	-0.520518000	7.696731000	18.015241000
6	-0.130200000	7.518297000	19.503011000
8	-0.952613000	7.160627000	20.346259000
6	-0.587294000	9.193483000	17.606321000
6	-0.713215000	9.339184000	16.083296000
6	-1.698712000	9.964906000	18.337053000
1	0.242134000	7.209383000	17.401681000
1	0.374371000	9.626727000	17.903316000
1	-0.716750000	10.397490000	15.797761000
1	0.125605000	8.848372000	15.574134000
1	-2.582627000	7.427115000	18.208040000
1	-1.639530000	8.884351000	15.714410000
1	-2.697179000	9.658806000	17.997581000
1	-1.652179000	9.821713000	19.422376000
1	-1.608644000	11.037292000	18.133292000
7	1.171090000	7.778386000	19.805578000
6	1.621804000	7.735400000	21.188382000
6	1.992127000	6.314271000	21.688828000
6	3.196605000	5.736059000	21.011090000
6	4.530585000	5.971292000	21.229726000
7	3.154724000	4.905535000	19.894174000
6	4.425469000	4.700746000	19.486626000
7	5.290836000	5.341741000	20.264179000
1	0.818857000	8.117911000	21.826081000
1	1.115311000	5.670243000	21.568592000
1	2.191558000	6.379086000	22.765537000
1	4.990268000	6.552989000	22.011546000
1	4.694702000	4.106361000	18.630747000
1	1.800688000	8.133170000	19.083344000
6	12.474672000	-1.481513000	18.936856000

6	12.020366000	-0.030849000	19.130837000
6	10.499380000	0.098978000	19.293675000
6	10.030250000	1.541504000	19.524834000
7	10.430321000	2.391548000	18.406165000
6	11.117196000	3.545735000	18.499574000
7	11.088898000	4.257605000	19.634417000
7	11.886995000	3.925974000	17.467441000
1	12.193592000	-2.105553000	19.794067000
1	12.353213000	0.573574000	18.278436000
1	12.514286000	0.394525000	20.015818000
1	10.158472000	-0.509755000	20.142124000
1	9.995513000	-0.309695000	18.404737000
1	10.490750000	1.947535000	20.430066000
1	8.943382000	1.582961000	19.667100000
1	10.338593000	2.013625000	17.466927000
1	11.653557000	5.122206000	19.688423000
1	10.212751000	4.252892000	20.152619000
1	11.771445000	3.361190000	16.630121000
1	11.956683000	4.934229000	17.249491000
26	7.269039000	5.844653000	19.690319000
6	8.171914000	5.215481000	24.506385000
6	8.604497000	4.457825000	23.250698000
8	8.182154000	4.356841000	20.874456000
6	8.072519000	5.039883000	21.956285000
8	7.542517000	6.181715000	21.905477000
1	7.082427000	5.209121000	24.614670000
1	8.298013000	3.405721000	23.288446000
1	9.700308000	4.444991000	23.164306000
1	8.497016000	6.258926000	24.466906000
7	9.109904000	7.370735000	16.426926000
6	10.222059000	8.390877000	16.456803000
6	11.489334000	7.557409000	16.850929000
8	11.487685000	6.393192000	16.366994000
6	9.908200000	9.536690000	17.407744000
6	8.791566000	10.481817000	16.943617000
6	9.236160000	11.528418000	15.906203000
7	8.190255000	12.490078000	15.589218000
6	7.871172000	13.522557000	16.395823000
7	8.554524000	13.810133000	17.529071000
7	6.885549000	14.340369000	16.045863000
8	12.335687000	8.095917000	17.596478000
1	8.846322000	7.102381000	17.394812000
1	10.344147000	8.738876000	15.424456000
1	10.840248000	10.087192000	17.565596000
1	9.659917000	9.114644000	18.388581000
1	9.537852000	6.506814000	16.050183000
1	8.397540000	11.005031000	17.822477000
1	7.942365000	9.925495000	16.528743000
1	9.529476000	11.048296000	14.966666000
1	10.119007000	12.073853000	16.261464000
1	7.414164000	12.181627000	14.986163000
1	8.048853000	14.477990000	18.138719000
1	9.063991000	13.070810000	17.989179000
1	6.384743000	14.163949000	15.154839000
1	6.469923000	14.856708000	16.825933000
8	10.095700000	2.577864000	15.526407000
1	9.590283000	3.380767000	15.804729000
1	10.264043000	2.674251000	14.577663000
8	8.703862000	6.392606000	19.143566000
7	12.373094000	9.772562000	20.190007000
6	12.697781000	8.777252000	21.225969000
6	11.972653000	7.457134000	20.921315000
8	12.598605000	6.660746000	19.912593000
1	12.613507000	9.408418000	19.267035000
1	12.258175000	9.147781000	22.164916000
1	10.927984000	7.676774000	20.657038000
1	11.974518000	6.835053000	21.824174000
1	12.602331000	7.155834000	19.055896000
1	1.540400000	0.383711000	18.981705000
1	0.258684000	-0.800551000	19.290537000
1	8.605476000	4.752153000	25.398664000
1	6.270647000	4.696293000	13.652962000
1	2.488110000	8.397261000	21.284758000
6	-3.411625000	3.716227000	17.090434000
1	-3.276166000	3.823386000	16.009372000
1	-2.679349000	2.991634000	17.459034000
1	-4.414646000	3.317636000	17.276082000
1	-3.970375000	5.786222000	17.435099000
1	-3.404163000	4.950860000	18.874301000
1	-0.154808000	5.774338000	12.936701000
1	1.208816000	6.787487000	13.350190000
1	1.746410000	2.864381000	12.265715000
1	0.155009000	3.624896000	12.045491000
1	-1.148004000	2.768216000	15.504047000
1	-0.655151000	1.258754000	12.844733000
1	-0.152592000	0.668843000	14.289980000
1	2.933414000	11.814719000	14.430272000
1	3.200455000	12.673721000	12.906572000
1	3.382813000	14.848540000	14.163426000
1	1.788605000	14.092463000	14.382760000
1	2.977641000	14.003211000	15.680569000
1	4.592768000	16.727097000	15.906100000
1	6.062046000	19.789428000	15.333505000
1	5.545402000	10.541732000	20.884667000
7	3.541653000	13.643421000	20.697084000
6	2.269024000	13.087688000	20.237707000
6	2.404560000	12.451376000	18.843711000
8	2.021144000	13.019587000	17.832937000
1	3.829574000	14.555983000	20.321753000
1	1.931701000	12.354840000	20.976398000
1	1.540487000	13.896223000	20.164048000
6	6.749064000	12.825308000	22.502285000
1	6.331431000	12.318841000	23.378223000
1	7.164395000	12.058598000	21.838391000
1	7.580814000	13.455124000	22.833589000
1	12.019456000	-1.924001000	18.042084000
1	13.562346000	-1.544078000	18.821906000
6	14.202398000	8.556599000	21.453048000
1	14.386821000	7.856089000	22.277939000
1	14.673261000	8.146477000	20.554198000
1	14.695715000	9.504178000	21.702111000
1	12.946246000	10.603654000	20.332598000
1	8.248918000	7.623917000	15.911633000
1	1.209785000	2.549719000	14.713146000
6	1.970936000	4.310745000	19.275557000
1	2.300912000	3.624917000	18.493089000
1	1.402212000	3.747261000	20.020241000
1	1.324830000	5.067398000	18.828966000

VioC MeHis316 Fe(III) complex

6	5.710115000	13.656083000	21.800615000
6	4.452454000	12.908365000	21.379243000
8	4.244886000	11.706774000	21.639019000
1	6.109089000	14.168178000	20.911386000
1	5.404785000	14.455535000	22.489751000
7	3.077549000	11.231943000	18.807257000

6	3.906025000	10.868341000	17.664992000
6	3.519847000	9.550632000	16.958545000
8	2.803611000	8.676268000	17.462794000
6	5.403099000	10.830183000	18.082916000
6	5.802526000	9.664607000	18.934634000
6	6.257584000	8.421343000	18.567806000
7	5.802555000	9.648637000	20.323319000
6	6.252987000	8.432170000	20.724251000
7	6.532029000	7.666919000	19.683117000
1	3.792534000	11.674553000	16.936210000
1	5.628457000	11.781904000	18.579665000
1	5.995385000	10.812714000	17.166125000
1	3.399951000	10.919509000	19.718794000
1	6.400517000	8.040326000	17.568384000
1	6.398586000	8.150705000	21.755703000
7	4.078700000	9.450277000	15.727114000
6	4.013384000	8.265845000	14.888897000
6	5.328119000	7.443060000	14.940818000
8	6.409043000	7.924938000	15.306957000
6	3.560304000	8.708590000	13.458363000
6	3.997156000	7.854740000	12.275013000
8	2.120287000	8.751667000	13.425902000
1	3.226597000	7.623804000	15.295303000
1	3.953625000	9.721061000	13.304247000
1	3.581543000	8.295176000	11.363030000
1	5.087668000	7.848231000	12.188798000
1	3.648850000	6.821942000	12.350815000
1	1.834411000	9.401993000	14.089083000
1	4.644641000	10.243908000	15.365684000
7	5.148825000	6.158588000	14.568347000
6	6.170987000	5.128109000	14.656777000
6	5.697773000	3.963335000	15.546736000
6	6.861539000	3.157021000	16.152701000
6	7.723382000	4.100850000	17.006879000
8	7.175525000	4.500130000	18.085395000
8	8.836519000	4.462845000	16.548605000
1	7.065135000	5.595228000	15.069351000
1	5.023560000	3.312719000	14.976454000
1	5.114516000	4.390113000	16.369374000
1	7.479851000	2.699290000	15.373149000
1	6.456270000	2.356081000	16.782269000
1	4.219470000	5.843695000	14.289724000
6	2.859494000	13.989621000	14.592507000
6	3.475352000	12.734455000	13.952464000
6	4.994167000	12.688373000	14.183008000
8	5.520221000	11.640536000	14.696908000
8	5.634022000	13.724687000	13.875956000
7	5.158606000	17.369523000	15.726879000
6	5.715729000	18.183065000	16.811760000
6	6.414810000	19.390054000	16.207881000
8	7.320737000	20.012166000	16.722499000
6	6.582174000	17.454856000	17.860107000
6	5.850214000	16.336735000	18.661239000
8	4.698295000	16.553213000	19.088407000
8	6.514829000	15.250025000	18.827964000
1	4.847284000	18.595975000	17.351848000
1	6.949298000	18.199141000	18.579074000
1	7.470244000	17.032582000	17.375231000
1	5.881420000	16.789538000	15.301234000
6	1.147661000	-0.595785000	18.682526000
6	0.823430000	-0.639549000	17.181950000
6	0.237404000	-2.012343000	16.792885000
6	2.066825000	-0.276591000	16.353426000

6	-0.360965000	-2.084638000	15.383010000
1	1.912498000	-1.340683000	18.938822000
1	0.047538000	0.120352000	16.988575000
1	-0.542345000	-2.279830000	17.519689000
1	1.023970000	-2.774318000	16.897113000
1	2.860517000	-1.020639000	16.502073000
1	1.858276000	-0.222521000	15.279305000
1	2.469789000	0.696292000	16.660144000
1	-1.184960000	-1.366588000	15.269539000
1	0.381668000	-1.870628000	14.606432000
1	-0.770807000	-3.080663000	15.179015000
7	0.135314000	1.236887000	13.534108000
6	0.435704000	2.622901000	13.939422000
6	-0.851998000	3.246476000	14.473279000
8	-1.526210000	4.056842000	13.865022000
6	1.020207000	3.407226000	12.757265000
6	1.654337000	4.735723000	13.176878000
7	0.872000000	5.832004000	13.088658000
8	2.833020000	4.762875000	13.550306000
6	-3.298975000	5.133380000	17.705540000
6	-1.892971000	5.708097000	17.519509000
8	-0.938751000	5.027597000	17.147044000
7	-1.804522000	7.048043000	17.794194000
6	-0.520542000	7.696713000	18.015224000
6	-0.085323000	7.467475000	19.483730000
8	-0.906665000	7.149344000	20.347119000
6	-0.558653000	9.205151000	17.649148000
6	-0.719243000	9.394771000	16.134580000
6	-1.632672000	9.980958000	18.428735000
1	0.201392000	7.203965000	17.359048000
1	0.421459000	9.604872000	17.931548000
1	-0.702498000	10.459839000	15.875555000
1	0.095205000	8.897900000	15.593035000
1	-2.551084000	7.409452000	18.375627000
1	-1.665561000	8.971116000	15.778359000
1	-2.645347000	9.697755000	18.111529000
1	-1.556413000	9.810600000	19.508467000
1	-1.527608000	11.056179000	18.246837000
7	1.236903000	7.639395000	19.738938000
6	1.749828000	7.534759000	21.099854000
6	2.079621000	6.088734000	21.554413000
6	3.294995000	5.485476000	20.916754000
6	4.623397000	5.577652000	21.263350000
7	3.288328000	4.751097000	19.734021000
6	4.585523000	4.462199000	19.436871000
7	5.421872000	4.955569000	20.329869000
1	0.992564000	7.940540000	21.778434000
1	1.188349000	5.470532000	21.397180000
1	2.246441000	6.121942000	22.638646000
1	5.059785000	6.066897000	22.121863000
1	4.882363000	3.912416000	18.558221000
1	1.852224000	7.989441000	19.002086000
6	12.408525000	-1.504755000	18.970109000
6	12.020355000	-0.030863000	19.130832000
6	10.503010000	0.181864000	19.218994000
6	10.106974000	1.652660000	19.404716000
7	10.615427000	2.452249000	18.298386000
6	11.318452000	3.596647000	18.397461000
7	11.348438000	4.281952000	19.543833000
7	12.067350000	3.969482000	17.341124000
1	12.054247000	-2.106036000	19.817126000
1	12.421957000	0.548307000	18.291229000
1	12.493429000	0.377901000	20.034889000

1	10.089269000	-0.394550000	20.058265000
1	10.023155000	-0.214218000	18.310858000
1	10.536750000	2.047226000	20.328571000
1	9.019712000	1.763074000	19.491656000
1	10.488346000	2.098793000	17.354462000
1	11.903376000	5.151380000	19.571813000
1	10.491139000	4.287799000	20.103243000
1	11.863898000	3.439816000	16.496089000
1	12.157516000	4.980242000	17.142782000
26	7.705603000	5.762487000	19.690451000
6	8.441354000	5.189170000	24.561353000
6	8.961763000	4.419261000	23.347942000
8	8.711806000	4.247881000	20.956875000
6	8.442035000	4.924276000	22.008089000
8	7.774483000	5.991512000	21.935032000
1	7.349801000	5.128357000	24.624821000
1	8.711534000	3.352902000	23.415221000
1	10.059011000	4.462755000	23.302091000
1	8.706929000	6.248248000	24.494067000
7	9.313409000	7.293900000	16.540371000
6	10.376240000	8.354570000	16.500537000
6	11.710329000	7.599321000	16.812595000
8	11.787748000	6.468304000	16.265146000
6	10.048395000	9.475099000	17.480952000
6	8.882370000	10.382287000	17.064074000
6	9.260059000	11.451444000	16.025865000
7	8.182321000	12.394289000	15.749126000
6	7.889181000	13.434474000	16.548206000
7	8.589997000	13.713780000	17.677005000
7	6.919850000	14.273364000	16.200851000
8	12.549193000	8.162870000	17.556466000
1	9.140056000	7.027889000	17.529052000
1	10.431633000	8.717348000	15.466094000
1	10.958017000	10.064407000	17.628635000
1	9.829533000	9.014443000	18.449494000
1	9.699664000	6.435327000	16.122063000
1	8.494951000	10.879499000	17.961966000
1	8.041386000	9.799300000	16.670573000
1	9.534386000	10.987443000	15.072672000
1	10.138108000	12.018038000	16.359526000
1	7.400676000	12.097889000	15.142555000
1	8.122869000	14.416617000	18.270290000
1	9.039996000	12.948920000	18.158896000
1	6.433925000	14.136468000	15.292924000
1	6.520585000	14.807669000	16.975139000
8	10.285480000	2.550119000	15.373192000
1	9.717323000	3.317516000	15.668759000
1	10.554293000	2.755601000	14.465977000
8	9.086332000	6.591223000	19.056777000
7	12.490906000	9.795034000	20.181679000
6	12.697794000	8.777300000	21.225980000
6	12.066325000	7.443471000	20.796029000
8	12.853913000	6.713123000	19.849221000
1	12.690516000	9.402295000	19.260375000
1	12.133653000	9.104760000	22.114546000
1	11.052333000	7.608794000	20.410287000
1	11.991182000	6.794553000	21.677451000
1	12.838598000	7.197085000	18.985548000
1	1.532103000	0.387244000	18.978601000
1	0.259678000	-0.806675000	19.290529000
1	8.862045000	4.783982000	25.489348000
1	6.425762000	4.764728000	13.651842000
1	2.649249000	8.153914000	21.168732000

6	-3.519578000	3.819915000	16.957717000
1	-3.374419000	3.955023000	15.880969000
1	-2.811601000	3.060305000	17.302680000
1	-4.535209000	3.445767000	17.128102000
1	-4.037612000	5.883777000	17.396008000
1	-3.444379000	4.986821000	18.785809000
1	-0.124575000	5.720666000	12.956075000
1	1.235723000	6.749208000	13.348076000
1	1.813908000	2.802968000	12.312544000
1	0.237372000	3.569648000	12.008322000
1	-1.205253000	2.832850000	15.437276000
1	-0.626207000	1.229160000	12.855209000
1	-0.170246000	0.683011000	14.332662000
1	2.999435000	11.826032000	14.340764000
1	3.304140000	12.756198000	12.867110000
1	3.380269000	14.879063000	14.226977000
1	1.795881000	14.078390000	14.339403000
1	2.929206000	13.955012000	15.683732000
1	4.466259000	16.730111000	16.112063000
1	5.999289000	19.684614000	15.217119000
1	5.451687000	10.390724000	20.935127000
7	3.566021000	13.652305000	20.668279000
6	2.296203000	13.101586000	20.197588000
6	2.444861000	12.454786000	18.809078000
8	2.079611000	13.029475000	17.792938000
1	3.873359000	14.551512000	20.282242000
1	1.948521000	12.375900000	20.938465000
1	1.571982000	13.912865000	20.108008000
6	6.762071000	12.762870000	22.457483000
1	6.354118000	12.234645000	23.325084000
1	7.144395000	12.009764000	21.759900000
1	7.613187000	13.366494000	22.789517000
1	11.973213000	-1.932076000	18.057791000
1	13.496214000	-1.626358000	18.907476000
6	14.167795000	8.592011000	21.638663000
1	14.264281000	7.885716000	22.474391000
1	14.753788000	8.202306000	20.800555000
1	14.603860000	9.547990000	21.957822000
1	13.142806000	10.566069000	20.321599000
1	8.428886000	7.544856000	16.078567000
1	1.173564000	2.571987000	14.748886000
6	2.130890000	4.294230000	18.978876000
1	2.485995000	3.752369000	18.099443000
1	1.514626000	3.618725000	19.582734000
1	1.513481000	5.129989000	18.646687000

WT VioC Fe(IV) complex

6	5.699545000	13.666189000	21.817158000
6	4.452424000	12.908361000	21.379231000
8	4.280517000	11.687117000	21.589482000
1	6.131916000	14.133166000	20.918638000
1	5.376963000	14.500649000	22.453163000
7	3.156039000	11.291744000	18.820455000
6	4.003475000	10.972977000	17.684514000
6	3.698102000	9.619081000	17.004928000
8	2.993417000	8.737426000	17.510375000
6	5.507464000	11.052875000	18.079173000
6	5.991752000	9.891321000	18.890401000
6	6.493057000	8.683150000	18.472287000
7	5.975679000	9.817294000	20.274990000
6	6.454164000	8.609945000	20.644901000
7	6.770395000	7.899329000	19.569688000
1	3.824770000	11.759808000	16.948142000

1	5.670874000	12.005712000	18.596687000
1	6.086999000	11.101630000	17.154851000
1	3.480864000	10.999139000	19.738161000
1	6.633980000	8.338996000	17.459239000
1	6.582732000	8.284878000	21.664236000
7	4.324132000	9.502693000	15.807759000
6	4.232545000	8.341075000	14.945427000
6	5.549380000	7.528102000	14.945509000
8	6.654408000	8.027485000	15.216208000
6	3.810529000	8.834517000	13.519823000
6	3.741033000	7.758146000	12.441525000
8	2.501999000	9.382201000	13.595197000
1	3.427962000	7.704864000	15.326395000
1	4.534550000	9.605305000	13.217846000
1	3.367055000	8.216070000	11.521289000
1	4.721164000	7.317042000	12.239637000
1	3.036410000	6.972661000	12.739309000
1	2.550278000	10.204655000	14.107050000
1	4.856096000	10.316131000	15.431264000
7	5.372584000	6.233683000	14.603244000
6	6.431180000	5.237056000	14.536845000
6	6.049461000	3.957250000	15.317917000
6	7.227904000	3.324192000	16.081064000
6	7.920627000	4.379160000	16.946573000
8	7.185032000	4.842210000	17.904009000
8	9.064361000	4.764203000	16.646230000
1	7.328380000	5.701292000	14.947717000
1	5.619183000	3.213442000	14.637435000
1	5.264359000	4.214640000	16.033412000
1	7.965425000	2.897667000	15.395578000
1	6.848616000	2.518977000	16.720504000
1	4.416743000	5.892210000	14.499341000
6	2.859472000	13.989591000	14.592505000
6	3.487470000	12.659779000	14.160646000
6	5.024775000	12.698140000	14.217241000
8	5.652675000	11.692539000	14.709468000
8	5.580747000	13.738353000	13.797309000
7	5.435459000	17.407597000	15.602527000
6	5.715740000	18.183079000	16.811768000
6	6.371592000	19.494315000	16.394621000
8	7.159109000	20.130606000	17.061280000
6	6.496438000	17.488177000	17.954851000
6	5.802624000	16.278638000	18.651117000
8	4.673427000	16.430518000	19.156520000
8	6.480703000	15.182481000	18.674019000
1	4.735006000	18.475972000	17.222352000
1	6.689207000	18.245123000	18.725320000
1	7.476591000	17.173426000	17.577244000
1	6.269377000	16.919130000	15.280510000
6	1.147661000	-0.595645000	18.682515000
6	1.136402000	0.848666000	19.207248000
6	1.204094000	0.859519000	20.747613000
6	-0.081047000	1.612255000	18.663205000
6	1.351235000	2.247904000	21.381220000
1	0.263779000	-1.146074000	19.031680000
1	2.039625000	1.353849000	18.830162000
1	2.051668000	0.234735000	21.063725000
1	0.302297000	0.369877000	21.145395000
1	-1.014292000	1.187901000	19.059484000
1	-0.048850000	2.673012000	18.932278000
1	-0.120549000	1.555304000	17.569121000
1	2.235389000	2.768468000	20.991806000
1	0.478413000	2.880830000	21.185259000

1	1.462820000	2.171870000	22.469090000
7	-0.259199000	1.537268000	14.650420000
6	0.435712000	2.622882000	13.939413000
6	0.205961000	2.402836000	12.450049000
8	1.060115000	2.106265000	11.634235000
6	1.912659000	2.644733000	14.364216000
6	2.664051000	3.874142000	13.876615000
7	3.132708000	3.833937000	12.611020000
8	2.852376000	4.851159000	14.620977000
6	-3.125009000	4.999446000	17.287452000
6	-1.806742000	5.758453000	17.140116000
8	-0.881748000	5.322462000	16.440059000
7	-1.745671000	6.929765000	17.823588000
6	-0.520509000	7.696650000	18.015254000
6	-0.078448000	7.531812000	19.481905000
8	-0.873186000	7.160511000	20.348690000
6	-0.671106000	9.190176000	17.608820000
6	-0.819434000	9.331329000	16.087519000
6	-1.817571000	9.885818000	18.358119000
1	0.240608000	7.245240000	17.373112000
1	0.271057000	9.673006000	17.897381000
1	-0.935551000	10.386017000	15.813192000
1	0.060496000	8.944180000	15.560335000
1	-2.430835000	7.060346000	18.559762000
1	-1.702391000	8.791892000	15.724056000
1	-2.792407000	9.487604000	18.049281000
1	-1.731958000	9.765456000	19.444099000
1	-1.818703000	10.958336000	18.136197000
7	1.223509000	7.819305000	19.735432000
6	1.751088000	7.716851000	21.087853000
6	2.218051000	6.286378000	21.460283000
6	3.424167000	5.828000000	20.702450000
6	4.755911000	5.855931000	21.040759000
7	3.393891000	5.360486000	19.397692000
6	4.658146000	5.141892000	19.001035000
7	5.523225000	5.436377000	19.968565000
1	0.961917000	8.012835000	21.784768000
1	1.375127000	5.601287000	21.316087000
1	2.457981000	6.274308000	22.530162000
1	5.214019000	6.150913000	21.971410000
1	4.932913000	4.789072000	18.023716000
1	1.829596000	8.169329000	18.990757000
6	12.376451000	-1.515263000	18.996008000
6	12.020366000	-0.030851000	19.130839000
6	10.511221000	0.230078000	19.046255000
6	10.145055000	1.709655000	19.206882000
7	10.768546000	2.505579000	18.153936000
6	11.430540000	3.663579000	18.321966000
7	11.317246000	4.356543000	19.460377000
7	12.277624000	4.072546000	17.358690000
1	11.906731000	-2.111779000	19.787749000
1	12.536424000	0.542383000	18.351170000
1	12.397445000	0.352491000	20.089466000
1	9.986101000	-0.337120000	19.826603000
1	10.122115000	-0.141561000	18.086206000
1	10.512115000	2.084108000	20.166445000
1	9.057004000	1.850490000	19.201898000
1	10.723455000	2.159786000	17.199460000
1	11.885422000	5.215752000	19.558171000
1	10.413385000	4.343604000	19.929083000
1	12.221961000	3.528035000	16.503075000
1	12.318678000	5.086904000	17.163633000
26	7.506990000	5.960791000	19.521939000

6	8.349782000	5.059233000	24.308882000
6	8.789394000	4.369163000	23.017333000
8	8.416102000	4.412096000	20.631448000
6	8.272647000	5.023381000	21.751510000
8	7.721710000	6.156337000	21.761198000
1	7.259556000	5.050966000	24.408905000
1	8.475645000	3.318568000	22.994997000
1	9.885695000	4.353049000	22.939265000
1	8.678476000	6.102137000	24.328778000
7	9.398559000	7.565185000	16.371793000
6	10.521629000	8.572431000	16.381341000
6	11.777130000	7.730288000	16.799215000
8	11.783298000	6.569587000	16.308327000
6	10.219402000	9.741595000	17.307517000
6	9.046597000	10.637195000	16.879373000
6	9.417838000	11.703941000	15.833889000
7	8.327044000	12.622931000	15.544478000
6	7.989718000	13.642291000	16.359559000
7	8.671711000	13.939291000	17.492739000
7	6.984989000	14.441068000	16.020697000
8	12.604876000	8.258946000	17.573523000
1	9.124107000	7.324233000	17.344796000
1	10.649012000	8.895799000	15.341630000
1	11.139604000	10.329355000	17.384179000
1	10.051632000	9.352576000	18.317900000
1	9.811603000	6.682625000	16.021297000
1	8.654040000	11.138552000	17.771646000
1	8.208473000	10.049205000	16.486281000
1	9.708115000	11.236374000	14.886921000
1	10.285275000	12.285470000	16.169374000
1	7.541700000	12.284284000	14.966179000
1	8.140857000	14.576925000	18.112546000
1	9.188480000	13.201842000	17.948294000
1	6.474570000	14.260459000	15.136089000
1	6.551198000	14.924078000	16.813763000
8	1.719779000	4.683407000	17.260969000
8	10.534490000	2.858319000	15.273220000
1	10.044607000	3.643954000	15.621012000
1	10.812189000	3.090221000	14.374666000
1	2.174111000	4.858035000	16.411889000
1	0.784236000	4.911531000	17.069893000
8	8.948561000	6.561894000	19.047469000
7	12.325013000	9.786468000	20.224664000
6	12.699514000	8.774738000	21.226964000
6	12.097486000	7.417249000	20.844131000
8	12.823776000	6.716785000	19.831277000
1	12.638859000	9.493714000	19.297962000
1	12.201681000	9.066333000	22.164680000
1	11.048660000	7.562842000	20.547823000
1	12.119070000	6.759430000	21.721231000
1	12.839509000	7.253652000	18.999815000
1	1.139874000	-0.619306000	17.585933000
1	2.034891000	-1.141628000	19.026375000
1	8.774886000	4.546359000	25.177847000
1	6.654314000	4.993296000	13.488795000
1	2.584514000	8.419579000	21.184668000
1	2.589593000	5.211262000	18.763467000
6	-3.291529000	3.883108000	16.257070000
1	-3.308222000	4.287609000	15.239415000
1	-2.462186000	3.172746000	16.326718000
1	-4.229238000	3.344027000	16.427171000
1	-3.958780000	5.710428000	17.232972000
1	-3.147844000	4.580144000	18.303572000
1	2.780556000	3.114556000	11.985725000
1	3.510878000	4.683126000	12.209791000
1	1.933137000	2.659966000	15.455447000
1	2.410506000	1.736110000	14.006247000
1	-0.863701000	2.428347000	12.150931000
1	-0.017619000	0.622982000	14.271381000
1	-1.269792000	1.644151000	14.609693000
1	3.114184000	11.853968000	14.804386000
1	3.192822000	12.421553000	13.128794000
1	3.276453000	14.805281000	13.996147000
1	1.772041000	13.972214000	14.458736000
1	3.050367000	14.188364000	15.651572000
1	4.730442000	16.698045000	15.786767000
1	6.046489000	19.841130000	15.388242000
1	5.564363000	10.518860000	20.907423000
7	3.554270000	13.661767000	20.701848000
6	2.290421000	13.118461000	20.204837000
6	2.472006000	12.490073000	18.813782000
8	2.100316000	13.049114000	17.793348000
1	3.858611000	14.572878000	20.332527000
1	1.926856000	12.382674000	20.928131000
1	1.569800000	13.932180000	20.114792000
6	6.724989000	12.804975000	22.553427000
1	6.281424000	12.306923000	23.421417000
1	7.148262000	12.031156000	21.903334000
1	7.554674000	13.428226000	22.902071000
1	12.038690000	-1.918899000	18.033364000
1	13.459028000	-1.670759000	19.059921000
6	14.209754000	8.670066000	21.497423000
1	14.426865000	7.948651000	22.295837000
1	14.742658000	8.344429000	20.598640000
1	14.612155000	9.642683000	21.806282000
1	12.808798000	10.659407000	20.433513000
1	8.546926000	7.816000000	15.841618000
1	-0.046773000	3.558630000	14.250817000

VioC WT Fe(III) complex

6	5.716307000	13.629552000	21.827321000
6	4.452426000	12.908362000	21.379236000
8	4.239184000	11.697061000	21.585774000
1	6.102575000	14.202564000	20.969403000
1	5.418303000	14.379787000	22.573051000
7	3.135077000	11.370158000	18.800629000
6	3.976297000	11.043923000	17.660039000
6	3.589842000	9.747331000	16.922423000
8	2.859630000	8.871491000	17.398594000
6	5.472184000	10.985478000	18.085069000
6	5.870744000	9.777141000	18.874812000
6	6.359408000	8.566876000	18.446204000
7	5.826206000	9.672559000	20.258537000
6	6.283147000	8.439180000	20.596813000
7	6.610272000	7.746890000	19.519816000
1	3.874806000	11.869272000	16.950913000
1	5.695021000	11.910802000	18.629911000
1	6.069293000	11.015391000	17.171297000
1	3.449991000	11.043751000	19.710054000
1	6.538752000	8.250717000	17.430202000
1	6.392186000	8.091127000	21.612274000
7	4.170112000	9.667363000	15.696453000
6	4.087419000	8.499662000	14.837673000
6	5.433396000	7.730406000	14.804404000
8	6.515399000	8.258059000	15.085543000
6	3.596083000	8.988602000	13.441315000

6	3.442861000	7.911414000	12.374276000
8	2.304542000	9.569845000	13.594559000
1	3.317216000	7.842953000	15.253481000
1	4.313378000	9.747813000	13.092941000
1	3.004121000	8.366253000	11.480620000
1	4.408743000	7.473200000	12.106960000
1	2.766420000	7.124055000	12.725862000
1	2.369691000	10.208585000	14.324260000
1	4.750705000	10.458015000	15.355225000
7	5.301642000	6.433516000	14.431412000
6	6.400791000	5.475328000	14.446921000
6	5.987141000	4.162643000	15.137017000
6	7.175207000	3.382846000	15.726242000
6	7.965762000	4.288552000	16.686601000
8	7.332463000	4.660625000	17.725388000
8	9.122872000	4.638493000	16.340573000
1	7.227491000	5.949205000	14.977080000
1	5.437216000	3.526047000	14.431944000
1	5.298409000	4.411109000	15.949046000
1	7.843854000	3.018958000	14.939375000
1	6.790787000	2.514580000	16.274722000
1	4.360371000	6.054339000	14.350169000
6	2.859477000	13.989573000	14.592505000
6	3.676718000	12.995814000	13.746847000
6	5.139684000	12.973275000	14.204447000
8	5.628963000	11.910179000	14.721761000
8	5.768224000	14.056214000	14.075146000
7	5.014621000	17.315468000	15.860799000
6	5.715741000	18.183082000	16.811769000
6	6.434848000	19.277969000	16.044026000
8	7.419395000	19.877548000	16.424223000
6	6.615472000	17.497435000	17.861789000
6	5.898521000	16.439802000	18.754975000
8	4.731339000	16.656313000	19.138441000
8	6.591814000	15.392275000	19.027345000
1	4.920128000	18.704577000	17.370384000
1	7.032071000	18.270420000	18.521469000
1	7.473860000	17.029927000	17.366132000
1	5.657182000	16.677715000	15.390896000
6	1.147663000	-0.595646000	18.682516000
6	1.158732000	0.828802000	19.259573000
6	1.194046000	0.782758000	20.800369000
6	-0.028310000	1.640315000	18.717771000
6	1.346158000	2.144905000	21.487667000
1	0.244221000	-1.137736000	18.994398000
1	2.080910000	1.324776000	18.920444000
1	2.027654000	0.135574000	21.108761000
1	0.277786000	0.289493000	21.161048000
1	-0.980543000	1.219678000	19.072239000
1	0.018995000	2.687400000	19.033465000
1	-0.038715000	1.630847000	17.621615000
1	2.238568000	2.673567000	21.131584000
1	0.481397000	2.792890000	21.305661000
1	1.442441000	2.025775000	22.573343000
7	-0.173710000	1.509171000	14.686876000
6	0.435713000	2.622890000	13.939414000
6	0.143678000	2.375791000	12.466239000
8	0.963494000	2.086618000	11.613369000
6	1.926739000	2.722831000	14.292904000
6	2.593019000	3.980860000	13.750413000
7	3.026168000	3.928950000	12.470773000
8	2.746492000	4.987129000	14.457942000
6	-3.133275000	4.989390000	17.355088000

6	-1.846070000	5.786640000	17.131961000
8	-0.985033000	5.413550000	16.323903000
7	-1.748841000	6.919804000	17.873289000
6	-0.520517000	7.696652000	18.015251000
6	-0.024142000	7.509061000	19.463558000
8	-0.807518000	7.162184000	20.355123000
6	-0.699828000	9.194316000	17.642065000
6	-0.917493000	9.358531000	16.131507000
6	-1.817296000	9.868208000	18.452288000
1	0.217371000	7.262104000	17.335949000
1	0.251799000	9.677582000	17.895797000
1	-1.050536000	10.416765000	15.878227000
1	-0.060566000	8.983748000	15.559859000
1	-2.346427000	6.978661000	18.692267000
1	-1.813261000	8.818997000	15.799742000
1	-2.800538000	9.461340000	18.183052000
1	-1.680193000	9.732303000	19.530902000
1	-1.838946000	10.943871000	18.245628000
7	1.291478000	7.744320000	19.668012000
6	1.877117000	7.609674000	20.996942000
6	2.263123000	6.154557000	21.363265000
6	3.447405000	5.609445000	20.626420000
6	4.768387000	5.509473000	21.001871000
7	3.417248000	5.153603000	19.317151000
6	4.685261000	4.823227000	18.971824000
7	5.537882000	5.032434000	19.961563000
1	1.145212000	7.969441000	21.726790000
1	1.376509000	5.523873000	21.225300000
1	2.496137000	6.135654000	22.435235000
1	5.222274000	5.767521000	21.947760000
1	4.963540000	4.447873000	18.001749000
1	1.872500000	8.095640000	18.904474000
6	12.364505000	-1.520337000	19.023993000
6	12.020364000	-0.030846000	19.130837000
6	10.525892000	0.254624000	18.931581000
6	10.169731000	1.742608000	19.042016000
7	10.865215000	2.510450000	18.017193000
6	11.522984000	3.672369000	18.195455000
7	11.450489000	4.338770000	19.348374000
7	12.346146000	4.085635000	17.207521000
1	11.826015000	-2.109729000	19.777074000
1	12.599212000	0.535471000	18.391512000
1	12.329851000	0.348819000	20.114809000
1	9.935343000	-0.294107000	19.678537000
1	10.205754000	-0.126687000	17.949700000
1	10.468643000	2.131379000	20.017615000
1	9.086537000	1.895801000	18.965955000
1	10.780545000	2.205951000	17.051100000
1	11.998903000	5.208305000	19.438253000
1	10.562828000	4.316142000	19.858631000
1	12.211671000	3.573243000	16.339681000
1	12.401051000	5.103623000	17.032594000
26	7.779156000	5.827837000	19.455152000
6	8.508964000	5.024990000	24.298313000
6	9.026102000	4.306834000	23.052234000
8	8.785768000	4.245989000	20.654948000
6	8.508040000	4.871014000	21.735265000
8	7.834097000	5.936287000	21.709890000
1	7.417450000	4.963363000	24.361329000
1	8.771835000	3.239443000	23.073617000
1	10.123492000	4.348099000	23.007688000
1	8.776234000	6.085546000	24.276176000
7	9.518677000	7.477790000	16.437225000

6	10.601678000	8.518245000	16.457966000
6	11.916362000	7.727450000	16.765644000
8	11.982078000	6.611258000	16.187209000
6	10.275988000	9.602142000	17.479461000
6	9.081269000	10.498226000	17.125940000
6	9.403375000	11.613158000	16.118707000
7	8.291351000	12.529325000	15.903719000
6	7.977469000	13.526956000	16.749497000
7	8.689607000	13.787074000	17.878292000
7	6.972957000	14.334431000	16.453067000
8	12.752706000	8.253405000	17.539586000
1	9.305482000	7.188018000	17.412268000
1	10.682037000	8.922163000	15.440521000
1	11.176629000	10.205880000	17.622696000
1	10.090688000	9.109605000	18.438405000
1	9.898006000	6.620165000	16.007833000
1	8.708487000	10.952402000	18.052457000
1	8.238937000	9.914422000	16.737326000
1	9.668701000	11.192053000	15.143187000
1	10.271495000	12.194058000	16.454283000
1	7.515569000	12.255027000	15.280219000
1	8.234434000	14.483965000	18.483545000
1	9.154914000	13.020305000	18.342021000
1	6.514861000	14.268046000	15.517649000
1	6.600234000	14.869425000	17.240034000
8	1.652310000	4.693988000	17.095399000
8	10.604017000	2.784814000	15.090583000
1	10.041107000	3.535001000	15.436228000
1	10.982271000	3.110160000	14.260413000
1	2.123300000	4.939260000	16.274095000
1	0.723931000	4.943245000	16.908630000
8	9.172951000	6.693362000	18.901974000
7	12.549362000	9.817339000	20.198183000
6	12.699513000	8.774739000	21.226963000
6	12.098380000	7.449952000	20.733642000
8	12.936724000	6.747755000	19.808759000
1	12.808217000	9.450098000	19.281065000
1	12.085240000	9.080130000	22.089718000
1	11.102352000	7.617280000	20.304529000
1	11.985737000	6.778787000	21.594089000
1	12.966447000	7.255527000	18.959444000
1	1.161949000	-0.578575000	17.585783000
1	2.015717000	-1.173676000	19.023089000
1	8.930410000	4.579521000	25.207309000
1	6.752970000	5.277616000	13.423878000
1	2.761110000	8.252495000	21.041400000
1	2.619792000	5.091305000	18.672689000
6	-3.295627000	3.830380000	16.372807000
1	-3.340712000	4.195105000	15.341371000
1	-2.449160000	3.140965000	16.449413000
1	-4.216883000	3.276540000	16.583731000
1	-3.989282000	5.673899000	17.297007000
1	-3.116137000	4.611701000	18.387227000
1	2.701202000	3.177041000	11.872226000
1	3.368288000	4.781471000	12.045631000
1	1.994670000	2.762190000	15.381699000
1	2.453042000	1.833375000	13.927640000
1	-0.940619000	2.369614000	12.218736000
1	0.109976000	0.606012000	14.310447000
1	-1.189665000	1.557112000	14.675627000
1	3.235442000	11.995215000	13.779767000
1	3.663826000	13.320846000	12.698627000
1	3.321725000	14.980157000	14.542991000

1	1.833776000	14.072483000	14.211680000
1	2.793148000	13.684483000	15.640706000
1	4.358620000	16.734918000	16.380118000
1	5.955332000	19.513741000	15.066445000
1	5.454656000	10.377281000	20.901076000
7	3.561379000	13.693420000	20.721603000
6	2.289247000	13.171468000	20.225237000
6	2.455946000	12.564409000	18.822099000
8	2.072448000	13.151087000	17.819752000
1	3.875447000	14.603355000	20.370571000
1	1.926460000	12.427298000	20.940274000
1	1.574316000	13.992189000	20.152011000
6	6.780138000	12.700202000	22.410767000
1	6.386567000	12.114954000	23.248018000
1	7.156618000	11.995934000	21.660949000
1	7.633035000	13.285404000	22.770093000
1	12.094363000	-1.919931000	18.038192000
1	13.437076000	-1.693861000	19.170106000
6	14.144732000	8.582886000	21.717808000
1	14.196275000	7.857656000	22.541177000
1	14.778492000	8.213916000	20.905408000
1	14.558994000	9.532068000	22.082936000
1	13.185435000	10.588410000	20.398021000
1	8.658077000	7.766705000	15.956778000
1	-0.077824000	3.536376000	14.266843000

Cartesian coordinates for model B

Wildtype

5Re - VioC reactant state

C	11.345235000000	4.335273000000	24.789841000000
C	11.446038000000	5.752310000000	24.244728000000
O	11.347152000000	6.006428000000	23.016545000000
C	12.690579000000	3.586758000000	24.998346000000
O	13.411623000000	3.427613000000	23.747993000000
C	12.452244000000	2.191733000000	25.575817000000
H	10.807580000000	4.344301000000	25.747771000000
H	13.318107000000	4.169163000000	25.688695000000
H	14.077316000000	4.170034000000	23.647992000000
H	13.409653000000	1.682597000000	25.718463000000
H	11.929651000000	2.238809000000	26.538280000000
H	11.850192000000	1.596359000000	24.879970000000
N	11.645243000000	6.758249000000	25.156083000000
C	11.560765000000	8.181930000000	24.775297000000
C	12.718854000000	8.588297000000	23.848621000000
O	12.555308000000	9.447374000000	22.941364000000
C	11.494632000000	9.074178000000	26.038668000000
C	10.328698000000	8.756997000000	27.013646000000
C	10.397138000000	9.727717000000	28.210632000000
C	8.951328000000	8.840235000000	26.325496000000
H	11.613132000000	6.543172000000	26.142912000000
H	10.657348000000	8.325598000000	24.174740000000
H	12.450766000000	8.997244000000	26.577457000000
H	11.392747000000	10.118236000000	25.719809000000
H	10.460135000000	7.729451000000	27.404104000000
H	8.843935000000	8.097711000000	25.523391000000
H	8.791023000000	9.839323000000	25.901434000000
H	8.154264000000	8.650362000000	27.055052000000
H	11.372435000000	9.672407000000	28.713148000000
H	10.235643000000	10.752676000000	27.856767000000
H	9.623218000000	9.482135000000	28.948574000000
N	13.912434000000	7.987294000000	24.082316000000
C	14.991519000000	7.927594000000	23.083785000000
C	15.086613000000	6.443431000000	22.719472000000
O	14.973282000000	5.577170000000	23.657918000000
C	16.333223000000	8.544241000000	23.575643000000
C	16.188932000000	10.071319000000	23.714739000000
C	16.846182000000	7.910514000000	24.882875000000
H	13.928128000000	7.221420000000	24.746601000000
H	14.653253000000	8.498214000000	22.214213000000
H	17.064292000000	8.336004000000	22.779293000000
H	16.937052000000	6.823145000000	24.803812000000
H	17.828653000000	8.324475000000	25.137758000000
H	16.166746000000	8.136656000000	25.714025000000
H	17.140300000000	10.519255000000	24.024005000000
H	15.433951000000	10.320934000000	24.468874000000
H	15.885371000000	10.534203000000	22.769462000000
N	15.212224000000	6.104707000000	21.433808000000
C	15.004927000000	4.713849000000	21.009496000000
C	13.489551000000	4.449260000000	20.915699000000
O	13.197388000000	3.017133000000	20.927090000000
H	15.142660000000	6.780697000000	20.658837000000
H	15.462896000000	4.038828000000	21.736670000000
H	12.961137000000	4.933930000000	21.737943000000
H	13.098811000000	4.846313000000	19.981199000000
H	13.161001000000	2.749594000000	21.878437000000
C	4.183570000000	11.115065000000	15.883872000000
C	3.367128000000	9.861869000000	15.603596000000
O	2.745818000000	9.243474000000	16.514743000000
C	5.703180000000	10.925320000000	15.582403000000
C	6.333414000000	9.823226000000	16.377002000000
N	6.887954000000	10.045689000000	17.633442000000
C	6.438809000000	8.464513000000	16.150213000000
C	7.305930000000	8.862606000000	18.138261000000
N	7.045663000000	7.882698000000	17.259354000000
H	3.801299000000	11.947321000000	15.277823000000
H	5.852046000000	10.731638000000	14.513826000000
H	6.212605000000	11.868936000000	15.808147000000
H	6.135674000000	7.882682000000	15.298251000000
H	7.765257000000	8.723719000000	19.099217000000
H	7.071747000000	10.991126000000	18.064138000000
N	3.387958000000	9.421313000000	14.325768000000
C	2.947083000000	8.092631000000	13.887452000000
C	4.181638000000	7.521671000000	13.160745000000
O	4.865661000000	8.286088000000	12.423837000000
C	1.684010000000	8.182455000000	12.990846000000
O	0.583568000000	8.767410000000	13.729021000000
C	1.281373000000	6.828167000000	12.395075000000
H	3.962121000000	9.892113000000	13.633530000000
H	2.709213000000	7.507089000000	14.780117000000
H	1.896023000000	8.893361000000	12.185976000000
H	0.188091000000	8.115775000000	14.360009000000
H	0.376112000000	6.954167000000	11.793777000000
H	2.071872000000	6.417410000000	11.757658000000
H	1.064762000000	6.110579000000	13.196690000000
N	4.518896000000	6.245064000000	13.429595000000
C	5.828315000000	5.688629000000	13.043996000000
C	5.922981000000	4.198104000000	13.426185000000
C	7.336071000000	3.787855000000	13.867424000000
C	7.681533000000	4.017846000000	15.336836000000
O	8.696164000000	3.424682000000	15.806301000000
O	6.914464000000	4.835266000000	16.038407000000
H	3.911156000000	5.668764000000	14.007840000000
H	6.620579000000	6.254344000000	13.548877000000
H	5.615836000000	3.578009000000	12.573811000000
H	5.224277000000	3.980323000000	14.239812000000
H	7.523042000000	2.727755000000	13.673511000000
H	8.093046000000	4.347028000000	13.299037000000
C	8.221656000000	-2.253926000000	14.619396000000
C	8.443777000000	-0.841243000000	15.192499000000
C	9.257712000000	0.036624000000	14.222412000000
C	7.105470000000	-0.166588000000	15.552790000000
H	7.642936000000	-2.205830000000	13.686664000000
H	9.025470000000	-0.944287000000	16.122764000000
H	10.239240000000	-0.406736000000	14.013455000000
H	8.730215000000	0.140073000000	13.261181000000
H	9.413198000000	1.040910000000	14.633085000000
H	7.274193000000	0.828361000000	15.980512000000
H	6.538832000000	-0.764762000000	16.278288000000
H	6.480746000000	-0.048229000000	14.656170000000
C	-3.587275000000	6.758384000000	20.537238000000
C	-3.364158000000	5.381357000000	21.186344000000
C	-2.472379000000	5.417330000000	22.452351000000
C	-2.585168000000	4.095791000000	23.238728000000
C	-1.000118000000	5.725750000000	22.110791000000
H	-2.643493000000	7.213891000000	20.218905000000
H	-4.340418000000	4.949637000000	21.454998000000
H	-2.907462000000	4.699800000000	20.452652000000
H	-2.848193000000	6.224482000000	23.102468000000
H	-1.972938000000	4.120026000000	24.148783000000
H	-3.621967000000	3.890557000000	23.532718000000
H	-2.240663000000	3.248230000000	22.627901000000
H	-0.884936000000	6.679708000000	21.587102000000
H	-0.388037000000	5.759022000000	23.021593000000
H	-0.594236000000	4.944020000000	21.451879000000
C	10.442551000000	12.536834000000	14.161469000000
C	11.764957000000	13.048644000000	14.730444000000
O	12.334481000000	14.029420000000	14.193981000000
C	9.475922000000	11.775042000000	15.103349000000
C	9.961659000000	10.367860000000	15.421112000000
O	10.498041000000	10.168206000000	16.587095000000
O	9.889341000000	9.472049000000	14.500920000000
H	9.940471000000	13.419964000000	13.754959000000
H	9.306905000000	12.323056000000	16.035029000000
H	8.519569000000	11.681575000000	14.577817000000
N	12.309475000000	12.371933000000	15.792938000000

C	13.573797000000	12.787080000000	16.391012000000
C	14.732842000000	11.792665000000	16.269670000000
O	15.761550000000	11.960153000000	16.970943000000
H	11.773761000000	11.639642000000	16.255432000000
H	13.873163000000	13.716395000000	15.895304000000
N	14.587599000000	10.785301000000	15.372067000000
C	15.639698000000	9.815747000000	15.075336000000
C	15.018981000000	8.460783000000	14.747363000000
O	14.038433000000	8.665528000000	13.699299000000
H	13.761936000000	10.721727000000	14.790245000000
H	16.303564000000	9.751968000000	15.941727000000
H	14.553615000000	8.024626000000	15.640456000000
H	15.808043000000	7.770832000000	14.413775000000
H	13.247267000000	8.080077000000	13.842751000000
C	5.696472000000	15.380475000000	20.013973000000
C	5.404633000000	15.366118000000	21.514360000000
O	5.339485000000	16.440929000000	22.177009000000
C	6.932354000000	14.592326000000	19.514523000000
C	6.765267000000	13.081022000000	19.363137000000
O	7.557992000000	12.481420000000	18.528679000000
O	5.920135000000	12.444091000000	20.097066000000
H	4.803458000000	15.017271000000	19.487416000000
H	7.239747000000	14.982146000000	18.538895000000
H	7.787708000000	14.764233000000	20.184140000000
N	5.184512000000	14.148639000000	22.078210000000
C	4.647313000000	13.996083000000	23.427717000000
C	5.555164000000	14.406739000000	24.593293000000
O	5.050705000000	14.603260000000	25.722630000000
H	5.283265000000	13.339676000000	21.448865000000
H	3.739319000000	14.597072000000	23.546978000000
H	4.373954000000	12.944759000000	23.562513000000
N	6.880946000000	14.537936000000	24.320106000000
C	7.851531000000	15.013985000000	25.303049000000
C	8.024911000000	16.552202000000	25.252720000000
O	8.891806000000	17.106230000000	25.980156000000
C	9.238474000000	14.352025000000	25.126369000000
C	9.310634000000	12.852423000000	25.456173000000
O	9.972226000000	12.493832000000	26.472479000000
O	8.712131000000	12.029111000000	24.625420000000
H	7.218016000000	14.265633000000	23.407468000000
H	7.451558000000	14.777731000000	26.295272000000
H	9.569570000000	14.501995000000	24.087962000000
H	9.928489000000	14.881501000000	25.784038000000
N	7.233726000000	17.238098000000	24.388142000000
C	7.343353000000	18.684591000000	24.210079000000
C	7.610254000000	19.073558000000	22.744309000000
C	8.895611000000	18.541664000000	22.125062000000
C	10.015705000000	18.168939000000	22.888899000000
C	8.982161000000	18.438255000000	20.723779000000
C	11.184875000000	17.714188000000	22.264661000000
C	10.150172000000	17.989347000000	20.098989000000
C	11.260498000000	17.625432000000	20.870687000000
H	6.549996000000	16.757226000000	23.808285000000
H	8.135679000000	19.030370000000	24.878304000000
H	6.761509000000	18.730063000000	22.138875000000
H	7.607384000000	20.174343000000	22.688548000000
H	9.976004000000	18.198068000000	23.972894000000
H	8.120657000000	18.713393000000	20.119498000000
H	12.034137000000	17.420646000000	22.874465000000
H	10.192122000000	17.917437000000	19.015895000000
H	12.167338000000	17.269152000000	20.391718000000
C	2.978436000000	0.450995000000	21.094952000000
C	1.680268000000	0.572137000000	20.329376000000
O	1.421991000000	-0.166013000000	19.336071000000
C	4.213428000000	0.872758000000	20.245832000000
C	5.512793000000	0.482290000000	20.989198000000
C	4.166769000000	2.374989000000	19.908486000000
C	6.793195000000	0.674792000000	20.159182000000
H	2.949555000000	1.043296000000	22.019138000000
H	4.168943000000	0.296656000000	19.309689000000
H	5.437979000000	-0.571992000000	21.294285000000
H	5.582228000000	1.070059000000	21.917651000000
H	7.665705000000	0.307723000000	20.712782000000
H	6.737107000000	0.112612000000	19.217975000000
H	6.975149000000	1.725670000000	19.909974000000
H	3.252194000000	2.640021000000	19.365035000000
H	5.015303000000	2.678515000000	19.289100000000
H	4.199058000000	2.979632000000	20.825227000000
N	0.798381000000	1.533893000000	20.734660000000
C	-0.512504000000	1.708680000000	20.096700000000
C	-0.428647000000	2.449874000000	18.757365000000
O	-0.833148000000	3.643713000000	18.654792000000
H	1.052985000000	2.163938000000	21.481120000000
H	-0.959753000000	0.720313000000	19.947552000000
N	0.099526000000	1.729505000000	17.739000000000
C	0.312314000000	2.272632000000	16.398325000000
C	1.807626000000	2.549199000000	16.120910000000
C	2.056586000000	3.415571000000	14.901075000000
O	2.865156000000	4.376992000000	14.936837000000
N	1.382523000000	3.101879000000	13.760274000000
H	0.486590000000	0.814168000000	17.986676000000
H	-0.250124000000	3.204288000000	16.326273000000
H	2.354425000000	1.602156000000	16.011616000000
H	2.234900000000	3.085333000000	16.968199000000
H	0.737662000000	2.330279000000	13.704409000000
H	1.526972000000	3.668076000000	12.936653000000
C	-3.084513000000	6.836311000000	16.225945000000
C	-1.694785000000	7.438633000000	16.336834000000
O	-0.752000000000	7.038751000000	15.570035000000
C	-3.048078000000	5.313335000000	16.020208000000
H	-3.678695000000	7.092855000000	17.110758000000
H	-2.614038000000	4.806087000000	16.888954000000
H	-4.060482000000	4.925338000000	15.867634000000
H	-2.444279000000	5.070434000000	15.140852000000
N	-1.515184000000	8.400693000000	17.259029000000
C	-0.239070000000	9.084957000000	17.497692000000
C	0.289286000000	8.652526000000	18.872797000000
O	-0.506533000000	8.302618000000	19.790266000000
C	-0.378830000000	10.637348000000	17.387284000000
C	-0.796662000000	11.047889000000	15.962495000000
C	-1.327285000000	11.235124000000	18.444976000000
H	-2.232232000000	8.550880000000	17.957090000000
H	0.459028000000	8.759399000000	16.725448000000
H	0.632989000000	11.022603000000	17.573043000000
H	-0.139716000000	10.611637000000	15.202247000000
H	-0.770837000000	12.139580000000	15.861897000000
H	-1.822748000000	10.721510000000	15.749992000000
H	-1.060767000000	10.930400000000	19.461728000000
H	-1.298480000000	12.329558000000	18.396613000000
H	-2.367344000000	10.932970000000	18.261339000000
N	1.630377000000	8.717098000000	19.020035000000
C	2.290610000000	8.363437000000	20.275239000000
C	2.623992000000	6.851123000000	20.383656000000
C	3.638099000000	6.362594000000	19.394315000000
N	3.305578000000	5.795378000000	18.162966000000
C	5.015420000000	6.380126000000	19.436866000000
C	4.451810000000	5.487616000000	17.502868000000
N	5.511159000000	5.832553000000	18.252325000000
H	2.201388000000	8.964357000000	18.207073000000
H	1.624539000000	8.635023000000	21.099021000000
H	1.689691000000	6.288061000000	20.278814000000
H	2.998572000000	6.667301000000	21.398760000000
H	5.677806000000	6.750094000000	20.198481000000
H	4.491900000000	5.046166000000	16.525421000000
H	2.334918000000	5.664906000000	17.796873000000
C	11.131545000000	0.285469000000	18.541623000000
C	10.554393000000	1.657046000000	18.924339000000
N	10.552727000000	2.613719000000	17.810534000000
C	11.602094000000	3.378545000000	17.456898000000
N	12.786254000000	3.321500000000	18.097444000000
N	11.473677000000	4.233194000000	16.423091000000
H	12.176021000000	0.374642000000	18.224626000000

H	10.563164000000	-0.158816000000	17.717489000000
H	9.513025000000	1.560731000000	19.242414000000
H	11.108552000000	2.079631000000	19.771196000000
H	9.676769000000	2.810050000000	17.323023000000
H	12.896888000000	2.850522000000	18.993931000000
H	13.454275000000	4.058110000000	17.857459000000
H	10.582745000000	4.286800000000	15.945095000000
H	12.169587000000	4.972649000000	16.339909000000
Fe	7.436421000000	5.892693000000	17.635083000000
O	7.904222000000	4.271272000000	19.067379000000
O	9.017389000000	6.071277000000	17.137474000000
C	8.123084000000	5.138299000000	20.004756000000
O	7.942499000000	6.393431000000	19.715039000000
C	8.572908000000	4.723212000000	21.379765000000
C	7.824826000000	5.467077000000	22.500029000000
H	8.449788000000	3.639124000000	21.465363000000
H	9.645461000000	4.944957000000	21.472366000000
H	6.746492000000	5.271297000000	22.460383000000
H	7.982130000000	6.545270000000	22.412055000000
N	11.468022000000	7.763596000000	17.255092000000
C	12.522408000000	7.995387000000	18.306187000000
C	13.646664000000	6.934904000000	18.208940000000
O	13.486988000000	5.965230000000	17.372898000000
C	11.925175000000	7.964408000000	19.732761000000
C	10.635097000000	8.761500000000	19.993717000000
C	10.757390000000	10.292230000000	19.871891000000
N	9.539890000000	10.979955000000	20.340736000000
C	9.253786000000	11.190810000000	21.648941000000
N	10.097703000000	10.850376000000	22.630718000000
N	8.089391000000	11.775635000000	22.008110000000
O	14.621382000000	7.096962000000	19.012724000000
H	12.955896000000	8.977174000000	18.089188000000
H	11.721750000000	6.918322000000	19.998791000000
H	12.709675000000	8.305555000000	20.414012000000
H	10.315120000000	8.512518000000	21.012246000000
H	9.835766000000	8.412870000000	19.332399000000
H	10.889416000000	10.588466000000	18.829168000000
H	11.626723000000	10.649492000000	20.439294000000
H	8.943690000000	11.434714000000	19.648156000000
H	11.012721000000	10.422901000000	22.504516000000
H	9.778235000000	11.097270000000	23.583266000000
H	7.303414000000	11.845087000000	21.369241000000
H	8.002662000000	12.001519000000	23.014382000000
H	11.881516000000	7.467232000000	16.344358000000
H	10.968597000000	8.680131000000	17.033810000000
H	10.773510000000	7.049573000000	17.518917000000
O	0.781664000000	5.437708000000	17.297010000000
H	0.110003000000	4.938739000000	17.819044000000
H	0.367781000000	6.004785000000	16.606608000000
O	11.738503000000	7.535105000000	14.469908000000
H	11.192882000000	8.379612000000	14.441882000000
H	11.097642000000	6.835761000000	14.195500000000
O	9.135690000000	6.718723000000	14.381420000000
H	9.056468000000	6.505530000000	15.341709000000
H	9.088466000000	7.699828000000	14.280765000000
H	11.092459000000	-0.397516000000	19.398546000000
H	3.085182000000	-0.602573000000	21.379864000000
H	-1.147222000000	2.297443000000	20.757651000000
H	-0.101862000000	1.561625000000	15.671907000000
H	7.670753000000	-2.889905000000	15.323923000000
H	9.176369000000	-2.745300000000	14.394600000000
H	-4.244838000000	6.669992000000	19.661402000000
H	-4.070568000000	7.447705000000	21.242224000000
H	3.208126000000	8.953923000000	20.355952000000
H	-3.574216000000	7.311441000000	15.363710000000
H	5.981254000000	5.835141000000	11.968975000000
H	4.044322000000	11.371147000000	16.936920000000
H	5.826360000000	16.434723000000	19.757076000000
H	10.677238000000	11.889303000000	13.304756000000
H	15.490776000000	4.582219000000	20.041634000000
H	10.750604000000	3.761863000000	24.072563000000
H	16.233910000000	10.159956000000	14.217076000000
H	13.450491000000	13.001337000000	17.457839000000
H	6.409851000000	19.165771000000	24.532014000000
H	8.192896000000	5.146912000000	23.480090000000

$^{5}TSH_{C3}$ – VioC WT transition state to HAT

C	11.399845000000	4.691605000000	25.900143000000
C	11.550559000000	5.929575000000	25.029753000000
O	11.363063000000	5.915964000000	23.787192000000
C	12.732018000000	4.013577000000	26.320033000000
O	13.398122000000	3.436836000000	25.171147000000
C	12.476840000000	2.882527000000	27.317644000000
H	10.836024000000	4.951825000000	26.806834000000
H	13.386934000000	4.767226000000	26.784809000000
H	14.111329000000	4.042506000000	24.825061000000
H	13.425628000000	2.413691000000	27.594437000000
H	11.983797000000	3.249247000000	28.225632000000
H	11.841394000000	2.117384000000	26.857510000000
N	11.917751000000	7.079099000000	25.686158000000
C	11.940226000000	8.399526000000	25.032024000000
C	13.027756000000	8.466479000000	23.948928000000
O	12.879141000000	9.158708000000	22.905944000000
C	12.122057000000	9.518009000000	26.089737000000
C	11.072961000000	9.528784000000	27.233747000000
C	11.413330000000	10.651890000000	28.236466000000
C	9.637616000000	9.696788000000	26.697400000000
H	11.951064000000	7.072021000000	26.696073000000
H	10.996041000000	8.547152000000	24.498435000000
H	13.130731000000	9.435819000000	26.520911000000
H	12.065346000000	10.481880000000	25.572052000000
H	11.133116000000	8.568443000000	27.781551000000
H	9.331184000000	8.854156000000	26.063128000000
H	9.570703000000	10.623814000000	26.115104000000
H	8.926128000000	9.757718000000	27.530084000000
H	12.444772000000	10.557952000000	28.603257000000
H	11.290119000000	11.639733000000	27.772995000000
H	10.743641000000	10.599968000000	29.104418000000
N	14.157941000000	7.758509000000	24.187241000000
C	15.093815000000	7.386312000000	23.116790000000
C	15.090959000000	5.856395000000	23.119719000000
O	15.139732000000	5.230613000000	24.232773000000
C	16.511906000000	8.008754000000	23.274661000000
C	16.446550000000	9.529469000000	23.037269000000
C	17.169613000000	7.684387000000	24.629379000000
H	14.183560000000	7.155972000000	25.001399000000
H	14.659757000000	7.743835000000	22.177902000000
H	17.122881000000	7.560513000000	22.475494000000
H	17.213195000000	6.607343000000	24.815845000000
H	18.189588000000	8.085785000000	24.656798000000
H	16.608968000000	8.149549000000	25.449573000000
H	17.448057000000	9.971164000000	23.099915000000
H	15.814779000000	10.012322000000	23.791236000000
H	16.027546000000	9.763386000000	22.052870000000
N	14.982408000000	5.252795000000	21.929898000000
C	14.756845000000	3.813260000000	21.799509000000
C	13.252879000000	3.510169000000	21.903617000000
O	12.968703000000	2.156883000000	21.397930000000
H	14.773102000000	5.820604000000	21.085922000000
H	15.319660000000	3.284156000000	22.574042000000
H	12.888335000000	3.618507000000	22.927064000000
H	12.696524000000	4.186295000000	21.253766000000
H	13.096958000000	1.490952000000	22.099373000000
C	3.741591000000	11.341967000000	16.859789000000
C	3.056635000000	10.107475000000	16.294794000000
O	2.361435000000	9.331228000000	17.011263000000
C	5.293963000000	11.271297000000	16.736440000000
C	5.909357000000	10.115388000000	17.467021000000
N	6.503276000000	10.284371000000	18.713523000000
C	6.017349000000	8.767285000000	17.179808000000
C	6.951864000000	9.082644000000	19.146763000000
N	6.672543000000	8.139015000000	18.235391000000
H	3.381986000000	12.235824000000	16.332980000000

H	5.578454000000	11.231229000000	15.678121000000
H	5.716149000000	12.200019000000	17.136324000000
H	5.685283000000	8.217200000000	16.317786000000
H	7.456795000000	8.911696000000	20.079994000000
H	6.735834000000	11.220548000000	19.139581000000
N	3.285200000000	9.865217000000	14.984324000000
C	2.979195000000	8.611741000000	14.289843000000
C	4.350437000000	8.120553000000	13.790784000000
O	5.173263000000	8.952483000000	13.317557000000
C	1.936162000000	8.838775000000	13.160789000000
O	0.709192000000	9.349577000000	13.721065000000
C	1.682881000000	7.573368000000	12.331566000000
H	3.910658000000	10.470659000000	14.464944000000
H	2.556223000000	7.920077000000	15.023067000000
H	2.314779000000	9.636666000000	12.512716000000
H	0.179588000000	8.626125000000	14.147547000000
H	0.907411000000	7.781475000000	11.588623000000
H	2.589586000000	7.252486000000	11.804232000000
H	1.329486000000	6.758695000000	12.974832000000
N	4.647951000000	6.813468000000	13.975106000000
C	6.031945000000	6.322604000000	13.843902000000
C	6.097024000000	4.799106000000	14.028447000000
C	7.469268000000	4.312308000000	14.530523000000
C	7.645383000000	4.307068000000	16.046860000000
O	8.334352000000	3.390632000000	16.568177000000
O	7.066955000000	5.284740000000	16.725210000000
H	3.945464000000	6.175712000000	14.347525000000
H	6.649442000000	6.811688000000	14.606048000000
H	5.859854000000	4.302510000000	13.078271000000
H	5.328588000000	4.484627000000	14.743372000000
H	7.670037000000	3.292368000000	14.192415000000
H	8.277399000000	4.942645000000	14.132338000000
C	5.672019000000	-1.750749000000	15.872171000000
C	6.349593000000	-0.409148000000	16.211504000000
C	7.738117000000	-0.284598000000	15.555753000000
C	5.448945000000	0.777898000000	15.817311000000
H	5.518674000000	-1.842089000000	14.787673000000
H	6.489378000000	-0.369271000000	17.303828000000
H	8.402471000000	-1.098322000000	15.873481000000
H	7.652568000000	-0.335232000000	14.461047000000
H	8.211449000000	0.669936000000	15.814371000000
H	5.907761000000	1.726833000000	16.117013000000
H	4.464649000000	0.692703000000	16.294612000000
H	5.302623000000	0.797970000000	14.727211000000
C	-3.943841000000	4.990312000000	19.441037000000
C	-3.607487000000	3.498859000000	19.610206000000
C	-2.623172000000	3.201270000000	20.768305000000
C	-2.576677000000	1.688227000000	21.061958000000
C	-1.209746000000	3.741725000000	20.469590000000
H	-3.048004000000	5.596008000000	19.263489000000
H	-4.540248000000	2.942973000000	19.790536000000
H	-3.179804000000	3.099408000000	18.678827000000
H	-2.998977000000	3.707117000000	21.672803000000
H	-1.872819000000	1.459188000000	21.872893000000
H	-3.562316000000	1.305346000000	21.353740000000
H	-2.264553000000	1.135849000000	20.163562000000
H	-1.210779000000	4.825866000000	20.315399000000
H	-0.523116000000	3.519101000000	21.297380000000
H	-0.815225000000	3.272328000000	19.556873000000
C	9.042432000000	11.443890000000	13.555160000000
C	9.655381000000	12.736845000000	14.091659000000
O	9.571097000000	13.793009000000	13.419234000000
C	8.624439000000	10.359648000000	14.581452000000
C	9.801644000000	9.561432000000	15.114818000000
O	10.324785000000	9.906330000000	16.243608000000
O	10.259294000000	8.586207000000	14.386065000000
H	8.167001000000	11.752034000000	12.975962000000
H	8.098033000000	10.816551000000	15.424154000000
H	7.937792000000	9.668684000000	14.082015000000
N	10.321344000000	12.675114000000	15.288618000000
C	10.961134000000	13.851296000000	15.868150000000
C	12.487348000000	13.775814000000	15.953526000000
O	13.120385000000	14.580317000000	16.684947000000
H	10.320029000000	11.811829000000	15.832239000000
H	10.691776000000	14.708464000000	15.242218000000
N	13.083174000000	12.826073000000	15.194524000000
C	14.520469000000	12.584889000000	15.159879000000
C	14.765721000000	11.078627000000	15.114446000000
O	14.021326000000	10.556835000000	13.988248000000
H	12.528489000000	12.212948000000	14.611227000000
H	14.970184000000	13.043299000000	16.044955000000
H	14.429302000000	10.622551000000	16.057527000000
H	15.842073000000	10.879175000000	15.003386000000
H	13.667277000000	9.645035000000	14.174088000000
C	6.232808000000	15.976548000000	20.684587000000
C	6.415109000000	16.280574000000	22.170120000000
O	6.703876000000	17.444069000000	22.571829000000
C	7.221170000000	14.974613000000	20.039421000000
C	6.947365000000	13.491740000000	20.278997000000
O	7.405236000000	12.666060000000	19.387223000000
O	6.355328000000	13.099494000000	21.350903000000
H	5.205547000000	15.622915000000	20.524470000000
H	7.236430000000	15.129645000000	18.956219000000
H	8.246265000000	15.183901000000	20.378077000000
N	6.218060000000	15.246159000000	23.031602000000
C	6.139944000000	15.448381000000	24.475030000000
C	7.446344000000	15.831752000000	25.182893000000
O	7.408306000000	16.349162000000	26.320649000000
H	6.079059000000	14.319579000000	22.606704000000
H	5.434977000000	16.252751000000	24.707919000000
H	5.762009000000	14.525681000000	24.926867000000
N	8.592496000000	15.568795000000	24.495711000000
C	9.929248000000	15.936421000000	24.963419000000
C	10.400691000000	17.291940000000	24.393056000000
O	11.593122000000	17.661616000000	24.564417000000
C	10.948503000000	14.830968000000	24.621101000000
C	10.720633000000	13.555066000000	25.449525000000
O	10.938109000000	13.605055000000	26.689892000000
O	10.305087000000	12.489068000000	24.795503000000
H	8.513256000000	15.114308000000	23.595883000000
H	9.876458000000	16.038019000000	26.053286000000
H	10.907521000000	14.602197000000	23.549055000000
H	11.940657000000	15.230617000000	24.848742000000
N	9.485703000000	18.031662000000	23.713764000000
C	9.811833000000	19.326745000000	23.116884000000
C	9.478286000000	19.386891000000	21.615911000000
C	10.257218000000	18.449632000000	20.701414000000
C	11.466292000000	17.838301000000	21.076421000000
C	9.762128000000	18.202771000000	19.406287000000
C	12.155433000000	17.011982000000	20.177755000000
C	10.455164000000	17.383357000000	18.508995000000
C	11.661813000000	16.779447000000	18.888883000000
H	8.536339000000	17.693604000000	23.583102000000
H	10.871605000000	19.509564000000	23.309732000000
H	8.405055000000	19.187216000000	21.495452000000
H	9.637808000000	20.426641000000	21.286946000000
H	11.868203000000	17.982368000000	22.074210000000
H	8.825334000000	18.665243000000	19.101605000000
H	13.084007000000	16.543734000000	20.491206000000
H	10.055336000000	17.214082000000	17.512561000000
H	12.199946000000	16.137602000000	18.196380000000
C	2.595857000000	-0.252091000000	19.890226000000
C	1.731676000000	-0.240799000000	18.644606000000
O	2.193349000000	-0.504328000000	17.496845000000
C	3.932266000000	0.513658000000	19.722341000000
C	4.831938000000	0.284085000000	20.960642000000
C	3.683435000000	2.009849000000	19.456770000000
C	6.276208000000	0.783626000000	20.786901000000
H	2.039741000000	0.153442000000	20.746675000000
H	4.439386000000	0.083978000000	18.847178000000
H	4.853509000000	-0.792828000000	21.186109000000
H	4.372759000000	0.773471000000	21.833579000000

H	6.873582000000	0.553769000000	21.677152000000
H	6.754679000000	0.294567000000	19.928655000000
H	6.329979000000	1.865141000000	20.622909000000
H	3.068980000000	2.156006000000	18.560610000000
H	4.621258000000	2.551397000000	19.302587000000
H	3.165248000000	2.475614000000	20.306279000000
N	0.408854000000	0.047339000000	18.816875000000
C	-0.562900000000	-0.028938000000	17.717294000000
C	-0.545818000000	1.205961000000	16.808332000000
O	-1.476759000000	2.049960000000	16.822310000000
H	0.074102000000	0.340855000000	19.723396000000
H	-0.346660000000	-0.928205000000	17.131029000000
N	0.546605000000	1.311752000000	16.007666000000
C	0.739956000000	2.485555000000	15.155375000000
C	2.016259000000	2.341496000000	14.309027000000
C	2.498397000000	3.693536000000	13.813611000000
O	2.728397000000	4.635341000000	14.621819000000
N	2.689830000000	3.837561000000	12.479486000000
H	1.298427000000	0.635926000000	16.146555000000
H	0.811263000000	3.386005000000	15.773404000000
H	1.858323000000	1.641667000000	13.481277000000
H	2.823523000000	1.933169000000	14.931678000000
H	2.500633000000	3.094759000000	11.825967000000
H	3.015646000000	4.725030000000	12.122147000000
C	-2.908582000000	6.222046000000	15.371903000000
C	-1.720584000000	7.114373000000	15.703420000000
O	-0.813933000000	7.328694000000	14.846813000000
C	-2.494430000000	4.897463000000	14.709116000000
H	-3.506832000000	6.031448000000	16.270470000000
H	-2.014337000000	4.222162000000	15.425287000000
H	-3.374337000000	4.382270000000	14.308842000000
H	-1.802811000000	5.097399000000	13.884598000000
N	-1.721347000000	7.671375000000	16.939306000000
C	-0.664104000000	8.539173000000	17.461248000000
C	-0.248475000000	7.989021000000	18.832325000000
O	-1.059907000000	7.325723000000	19.541216000000
C	-1.104041000000	10.038525000000	17.568519000000
C	-1.358916000000	10.637933000000	16.173501000000
C	-2.323237000000	10.233520000000	18.489868000000
H	-2.390025000000	7.352197000000	17.629893000000
H	0.183174000000	8.491678000000	16.773414000000
H	-0.243392000000	10.558918000000	18.012242000000
H	-0.491447000000	10.530086000000	15.514541000000
H	-1.595786000000	11.705342000000	16.260873000000
H	-2.214299000000	10.149149000000	15.690586000000
H	-2.165674000000	9.806606000000	19.485679000000
H	-2.538601000000	11.301873000000	18.606222000000
H	-3.216054000000	9.763929000000	18.057637000000
N	1.012112000000	8.287099000000	19.226357000000
C	1.516702000000	7.923772000000	20.551688000000
C	2.005091000000	6.454362000000	20.670979000000
C	3.179749000000	6.103900000000	19.809430000000
N	3.071605000000	5.551817000000	18.524040000000
C	4.533105000000	6.249631000000	20.030558000000
C	4.332778000000	5.388630000000	18.026670000000
N	5.239237000000	5.803350000000	18.915287000000
H	1.631285000000	8.765709000000	18.565876000000
H	0.713076000000	8.075423000000	21.279435000000
H	1.156457000000	5.794008000000	20.463004000000
H	2.280807000000	6.288384000000	21.720062000000
H	5.051187000000	6.650386000000	20.884241000000
H	4.548870000000	5.000046000000	17.048541000000
C	10.453669000000	0.152208000000	19.111174000000
C	10.130230000000	1.608532000000	19.477684000000
N	10.291373000000	2.532281000000	18.349359000000
C	11.410014000000	3.216633000000	18.054781000000
N	12.553386000000	3.077728000000	18.760491000000
N	11.416838000000	4.078680000000	17.022908000000
H	11.498588000000	0.049887000000	18.797489000000
H	9.817622000000	-0.192669000000	18.288751000000
H	9.090077000000	1.704636000000	19.801861000000
H	10.754721000000	1.932406000000	20.316958000000
H	9.452566000000	2.795783000000	17.823387000000
H	12.633360000000	2.495096000000	19.591753000000
H	13.287795000000	3.755225000000	18.564369000000
H	10.582693000000	4.308649000000	16.498566000000
H	12.185899000000	4.746700000000	16.977483000000
Fe	7.330398000000	6.157337000000	18.492928000000
O	7.833854000000	4.497932000000	19.862595000000
O	9.038411000000	6.637351000000	18.194095000000
C	7.857906000000	5.301414000000	20.881120000000
O	7.515028000000	6.541184000000	20.678703000000
C	8.301517000000	4.830922000000	22.240432000000
C	7.517086000000	5.476702000000	23.393682000000
H	8.224918000000	3.738944000000	22.262058000000
H	9.367226000000	5.080624000000	22.356846000000
H	6.454061000000	5.210135000000	23.353927000000
H	7.595770000000	6.566166000000	23.345785000000
N	11.934004000000	7.896575000000	17.157825000000
C	12.295619000000	7.773946000000	18.612040000000
C	13.356553000000	6.662737000000	18.816884000000
O	13.458545000000	5.746081000000	17.923117000000
C	11.050621000000	7.492947000000	19.495861000000
C	10.560793000000	8.580025000000	20.453031000000
C	10.438977000000	10.011884000000	19.874819000000
N	9.529311000000	10.882842000000	20.642673000000
C	9.770865000000	11.357479000000	21.896310000000
N	10.891510000000	11.095634000000	22.575420000000
N	8.855788000000	12.141984000000	22.499875000000
O	14.005951000000	6.752652000000	19.911579000000
H	12.751034000000	8.721943000000	18.911529000000
H	10.069916000000	7.170010000000	18.780920000000
H	11.173760000000	6.554822000000	20.046583000000
H	11.248549000000	8.577384000000	21.311992000000
H	9.586177000000	8.262585000000	20.842610000000
H	10.031287000000	9.979644000000	18.859451000000
H	11.431749000000	10.480483000000	19.807551000000
H	8.803318000000	11.379804000000	20.122925000000
H	11.613251000000	10.431732000000	22.317535000000
H	10.949488000000	11.545314000000	23.507342000000
H	7.921848000000	12.289375000000	22.122449000000
H	9.110971000000	12.457463000000	23.450344000000
H	12.749439000000	7.952364000000	16.524951000000
H	11.341372000000	8.748233000000	16.934305000000
H	11.391404000000	7.078503000000	16.799534000000
O	12.854679000000	8.190910000000	14.558716000000
H	11.854984000000	8.342757000000	14.425113000000
H	13.156164000000	7.406125000000	14.067915000000
O	10.105058000000	6.235243000000	15.794597000000
H	9.526365000000	6.389467000000	16.597174000000
H	10.003199000000	6.965315000000	15.131778000000
H	10.289936000000	-0.504654000000	19.974291000000
H	2.815113000000	-1.304434000000	20.120748000000
H	-1.562761000000	-0.106138000000	18.143579000000
H	-0.138554000000	2.615392000000	14.513920000000
H	4.692793000000	-1.829187000000	16.359843000000
H	6.288323000000	-2.599955000000	16.193182000000
H	-4.633270000000	5.139843000000	18.599996000000
H	-4.431208000000	5.387586000000	20.341106000000
H	2.338643000000	8.604407000000	20.791614000000
H	-3.543714000000	6.793166000000	14.679844000000
H	6.431956000000	6.623148000000	12.869583000000
H	3.451246000000	11.429359000000	17.909221000000
H	6.337417000000	16.939586000000	20.178862000000
H	9.748625000000	11.006535000000	12.835300000000
H	15.124069000000	3.494341000000	20.819522000000
H	10.817502000000	3.961667000000	25.331878000000
H	14.964083000000	13.051488000000	14.270060000000
H	10.589835000000	14.038703000000	16.880782000000
H	9.244470000000	20.115710000000	23.629603000000
H	7.916378000000	5.144150000000	24.357452000000
C	1.844300000000	5.176733000000	17.819677000000

H	2.109636000000	4.899023000000	16.798788000000
H	1.354187000000	4.335154000000	18.319297000000
H	1.156537000000	6.020068000000	17.786089000000

$^{5}I_{C3}$ – VioC WT – Intermediate

C	11.219647000000	4.373719000000	25.283144000000
C	11.393528000000	5.763818000000	24.690218000000
O	11.275342000000	5.984152000000	23.458040000000
C	12.523736000000	3.550499000000	25.463836000000
O	13.187246000000	3.317442000000	24.192927000000
C	12.226156000000	2.188343000000	26.090859000000
H	10.720076000000	4.443425000000	26.258985000000
H	13.212633000000	4.111844000000	26.111456000000
H	13.918624000000	3.993915000000	24.058718000000
H	13.156330000000	1.625747000000	26.211661000000
H	11.746472000000	2.293919000000	27.070905000000
H	11.561651000000	1.611927000000	25.437160000000
N	11.689945000000	6.783280000000	25.559335000000
C	11.703365000000	8.191437000000	25.115756000000
C	12.908192000000	8.485416000000	24.206439000000
O	12.840979000000	9.345169000000	23.289043000000
C	11.652840000000	9.152493000000	26.328557000000
C	10.468716000000	8.924870000000	27.306167000000
C	10.534001000000	9.964995000000	28.445232000000
C	9.103792000000	8.992924000000	26.592529000000
H	11.653246000000	6.611680000000	26.554580000000
H	10.827264000000	8.358483000000	24.481991000000
H	12.601515000000	9.084017000000	26.881459000000
H	11.576149000000	10.172077000000	25.935011000000
H	10.577602000000	7.922756000000	27.764183000000
H	8.982039000000	8.189914000000	25.853161000000
H	9.000635000000	9.961908000000	26.088061000000
H	8.290767000000	8.893570000000	27.322388000000
H	11.519766000000	9.964295000000	28.930294000000
H	10.327198000000	10.973282000000	28.062833000000
H	9.783330000000	9.735726000000	29.212182000000
N	14.043566000000	7.784625000000	24.455750000000
C	15.108695000000	7.638114000000	23.449767000000
C	15.064879000000	6.156361000000	23.070539000000
O	14.920764000000	5.286278000000	24.003493000000
C	16.499974000000	8.133418000000	23.935964000000
C	16.483964000000	9.665205000000	24.096535000000
C	16.972904000000	7.442477000000	25.229376000000
H	13.993868000000	7.025948000000	25.125616000000
H	14.809195000000	8.237407000000	22.584640000000
H	17.203899000000	7.877047000000	23.129044000000
H	16.968966000000	6.352567000000	25.135368000000
H	17.989645000000	7.768169000000	25.479262000000
H	16.323322000000	7.713931000000	26.070950000000
H	17.473795000000	10.031664000000	24.393209000000
H	15.765237000000	9.963613000000	24.868189000000
H	16.198450000000	10.162474000000	23.163226000000
N	15.093659000000	5.844818000000	21.773775000000
C	14.744457000000	4.492091000000	21.328326000000
C	13.207413000000	4.321691000000	21.325605000000
O	12.831483000000	2.916786000000	21.494257000000
H	15.061696000000	6.587567000000	21.037423000000
H	15.195037000000	3.758050000000	22.001931000000
H	12.750068000000	4.912078000000	22.121033000000
H	12.798596000000	4.655906000000	20.371972000000
H	12.810291000000	2.742197000000	22.472071000000
C	3.885817000000	11.179602000000	16.314965000000
C	3.245347000000	9.885956000000	15.837923000000
O	2.542506000000	9.166045000000	16.604804000000
C	5.441999000000	11.158767000000	16.242109000000
C	6.068204000000	10.016355000000	16.986854000000
N	6.722383000000	10.190157000000	18.202023000000
C	6.131066000000	8.659882000000	16.723439000000
C	7.157558000000	8.983233000000	18.639804000000
N	6.815397000000	8.029807000000	17.758314000000
H	3.516732000000	12.018770000000	15.710860000000
H	5.764276000000	11.154907000000	15.194750000000
H	5.815935000000	12.100561000000	16.657072000000
H	5.743199000000	8.103399000000	15.889598000000
H	7.681318000000	8.813352000000	19.563640000000
H	6.970604000000	11.111007000000	18.646338000000
N	3.507464000000	9.519551000000	14.563291000000
C	3.191841000000	8.203306000000	14.000886000000
C	4.531975000000	7.702887000000	13.429508000000
O	5.327757000000	8.527635000000	12.899491000000
C	2.046259000000	8.289014000000	12.952405000000
O	0.854061000000	8.837152000000	13.562948000000
C	1.758480000000	6.939656000000	12.284598000000
H	4.142044000000	10.068716000000	13.992814000000
H	2.860859000000	7.564804000000	14.825594000000
H	2.339987000000	9.025275000000	12.197022000000
H	0.374039000000	8.162410000000	14.104450000000
H	0.906353000000	7.048998000000	11.607113000000
H	2.618311000000	6.583718000000	11.707106000000
H	1.509094000000	6.177158000000	13.033100000000
N	4.846136000000	6.400012000000	13.609850000000
C	6.225212000000	5.927379000000	13.395308000000
C	6.341102000000	4.410681000000	13.615142000000
C	7.717450000000	3.982606000000	14.150185000000
C	7.890254000000	4.094104000000	15.666465000000
O	8.769770000000	3.389342000000	16.223085000000
O	7.093971000000	4.941515000000	16.301988000000
H	4.172018000000	5.747454000000	14.005388000000
H	6.877179000000	6.451073000000	14.103007000000
H	6.140639000000	3.884929000000	12.670815000000
H	5.581864000000	4.089542000000	14.333568000000
H	7.935186000000	2.942783000000	13.887706000000
H	8.518637000000	4.583414000000	13.693716000000
C	6.613989000000	-1.910829000000	15.202923000000
C	7.205872000000	-0.606927000000	15.771834000000
C	8.338791000000	-0.068351000000	14.876563000000
C	6.112349000000	0.459856000000	15.977154000000
H	6.170371000000	-1.734109000000	14.212810000000
H	7.637119000000	-0.834471000000	16.759629000000
H	9.147482000000	-0.802746000000	14.774030000000
H	7.959592000000	0.148592000000	13.867336000000
H	8.761473000000	0.856833000000	15.285060000000
H	6.532555000000	1.367004000000	16.425260000000
H	5.313509000000	0.088352000000	16.632403000000
H	5.659092000000	0.736091000000	15.013386000000
C	-3.936362000000	6.167212000000	19.880179000000
C	-3.654610000000	4.735083000000	20.366442000000
C	-2.822866000000	4.663571000000	21.671324000000
C	-2.881960000000	3.248652000000	22.281503000000
C	-1.361322000000	5.100835000000	21.444516000000
H	-3.011225000000	6.717659000000	19.677979000000
H	-4.612223000000	4.217422000000	20.529685000000
H	-3.122885000000	4.177844000000	19.580124000000
H	-3.278238000000	5.357134000000	22.397270000000
H	-2.307069000000	3.193259000000	23.214412000000
H	-3.914085000000	2.948211000000	22.500840000000
H	-2.463627000000	2.506271000000	21.585861000000
H	-1.289511000000	6.125543000000	21.066728000000
H	-0.787682000000	5.039738000000	22.378822000000
H	-0.882799000000	4.446471000000	20.701059000000
C	8.502774000000	10.384933000000	13.303885000000
C	8.349293000000	11.677090000000	14.108644000000
O	7.582242000000	12.582290000000	13.686561000000
C	8.747815000000	9.047370000000	14.052212000000
C	10.148353000000	8.831895000000	14.600428000000
O	10.493402000000	9.445298000000	15.685862000000
O	10.941647000000	8.018347000000	13.972504000000
H	7.575418000000	10.279097000000	12.734369000000
H	8.037161000000	8.960362000000	14.880142000000
H	8.545137000000	8.240026000000	13.344907000000
N	9.110553000000	11.816059000000	15.233019000000
C	9.211218000000	13.078385000000	15.960934000000
C	10.633696000000	13.644081000000	16.018438000000

O	10.883925000000	14.659454000000	16.717878000000
H	9.614547000000	10.998914000000	15.594809000000
H	8.565455000000	13.803280000000	15.455865000000
N	11.573332000000	12.986798000000	15.294875000000
C	12.990276000000	13.327968000000	15.279393000000
C	13.806873000000	12.038172000000	15.245634000000
O	13.355010000000	11.279651000000	14.098965000000
H	11.318337000000	12.191665000000	14.723708000000
H	13.215444000000	13.925079000000	16.167483000000
H	13.647954000000	11.479208000000	16.180234000000
H	14.878301000000	12.278269000000	15.168498000000
H	13.482421000000	10.300632000000	14.223673000000
C	6.361373000000	15.783202000000	20.378447000000
C	6.317688000000	16.001914000000	21.889670000000
O	6.568492000000	17.131008000000	22.399385000000
C	7.407999000000	14.784398000000	19.828976000000
C	7.077204000000	13.301749000000	19.950788000000
O	7.674725000000	12.502199000000	19.112414000000
O	6.304702000000	12.869820000000	20.881069000000
H	5.361058000000	15.476794000000	20.044177000000
H	7.580553000000	14.992380000000	18.768472000000
H	8.381483000000	14.951289000000	20.311466000000
N	5.953881000000	14.930860000000	22.647338000000
C	5.619997000000	15.062202000000	24.062354000000
C	6.778071000000	15.388915000000	25.013336000000
O	6.536434000000	15.859062000000	26.146850000000
H	5.858381000000	14.035559000000	22.151376000000
H	4.888098000000	15.863344000000	24.207756000000
H	5.162376000000	14.123266000000	24.390377000000
N	8.027949000000	15.126357000000	24.541039000000
C	9.256836000000	15.433120000000	25.273500000000
C	9.864453000000	16.791515000000	24.862019000000
O	11.017569000000	17.108023000000	25.259791000000
C	10.293840000000	14.307318000000	25.091265000000
C	9.876794000000	13.010917000000	25.807738000000
O	9.809402000000	13.020024000000	27.066158000000
O	9.620923000000	11.967321000000	25.043111000000
H	8.113795000000	14.720521000000	23.618522000000
H	8.994045000000	15.496133000000	26.335628000000
H	10.458227000000	14.118673000000	24.023449000000
H	11.232822000000	14.665913000000	25.522880000000
N	9.106900000000	17.596079000000	24.070820000000
C	9.588349000000	18.891065000000	23.591964000000
C	9.519330000000	19.014359000000	22.058773000000
C	10.323116000000	17.995571000000	21.262299000000
C	11.467708000000	17.357344000000	21.771297000000
C	9.912968000000	17.681368000000	19.952128000000
C	12.174136000000	16.432449000000	20.989159000000
C	10.613594000000	16.757299000000	19.169923000000
C	11.750970000000	16.127149000000	19.691423000000
H	8.189175000000	17.297984000000	23.751097000000
H	10.607515000000	19.007971000000	23.967763000000
H	8.467470000000	18.939590000000	21.754190000000
H	9.848582000000	20.032934000000	21.795765000000
H	11.795993000000	17.546918000000	22.788305000000
H	9.025496000000	18.164990000000	19.549062000000
H	13.048421000000	15.941162000000	21.407309000000
H	10.281749000000	16.494617000000	18.171428000000
H	12.277232000000	15.400488000000	19.081870000000
C	2.762406000000	0.341269000000	20.729747000000
C	1.654546000000	0.422118000000	19.702730000000
O	1.715176000000	-0.206509000000	18.607071000000
C	4.123195000000	0.848545000000	20.174270000000
C	5.252916000000	0.529524000000	21.181658000000
C	4.056311000000	2.348197000000	19.830580000000
C	6.668496000000	0.785677000000	20.637985000000
H	2.500387000000	0.902197000000	21.636797000000
H	4.318050000000	0.282897000000	19.250977000000
H	5.169809000000	-0.526011000000	21.481238000000
H	5.094977000000	1.124256000000	22.094717000000
H	7.423781000000	0.495742000000	21.378085000000
H	6.849691000000	0.196493000000	19.729651000000
H	6.834085000000	1.839129000000	20.389146000000
H	3.264356000000	2.562142000000	19.102895000000
H	4.995929000000	2.709982000000	19.404074000000
H	3.853915000000	2.945877000000	20.729719000000
N	0.584134000000	1.217621000000	19.996048000000
C	-0.568948000000	1.338330000000	19.094525000000
C	-0.296724000000	2.270452000000	17.908506000000
O	-0.851096000000	3.406884000000	17.840228000000
H	0.589789000000	1.773938000000	20.838511000000
H	-0.829824000000	0.338433000000	18.732083000000
N	0.565128000000	1.787315000000	16.989291000000
C	0.999763000000	2.552009000000	15.814578000000
C	2.321414000000	1.987584000000	15.274361000000
C	2.947020000000	2.941705000000	14.271505000000
O	3.064930000000	4.169826000000	14.522509000000
N	3.389522000000	2.398623000000	13.107497000000
H	1.000313000000	0.886645000000	17.204805000000
H	1.141225000000	3.599620000000	16.091066000000
H	2.178528000000	0.991752000000	14.838524000000
H	3.032748000000	1.879490000000	16.104344000000
H	3.309008000000	1.414098000000	12.909877000000
H	3.855843000000	2.989127000000	12.434092000000
C	-3.084549000000	6.658575000000	15.588058000000
C	-1.747023000000	7.321484000000	15.867031000000
O	-0.730628000000	7.061325000000	15.136660000000
C	-2.934559000000	5.176806000000	15.206345000000
H	-3.748450000000	6.771412000000	16.453526000000
H	-2.509800000000	4.594332000000	16.031189000000
H	-3.909900000000	4.749714000000	14.950580000000
H	-2.271790000000	5.078568000000	14.341876000000
N	-1.691338000000	8.177416000000	16.904042000000
C	-0.482435000000	8.904737000000	17.306309000000
C	-0.083543000000	8.445973000000	18.715719000000
O	-0.956128000000	8.051481000000	19.540812000000
C	-0.682282000000	10.453059000000	17.234857000000
C	-0.918654000000	10.906781000000	15.781993000000
C	-1.797130000000	10.958869000000	18.170995000000
H	-2.461400000000	8.208503000000	17.560269000000
H	0.309231000000	8.640039000000	16.604681000000
H	0.273081000000	10.876257000000	17.574670000000
H	-0.140504000000	10.536243000000	15.105942000000
H	-0.935179000000	12.001949000000	15.726209000000
H	-1.886574000000	10.544084000000	15.412774000000
H	-1.655094000000	10.622589000000	19.202805000000
H	-1.822133000000	12.054526000000	18.166375000000
H	-2.783125000000	10.611885000000	17.833682000000
N	1.234206000000	8.552535000000	18.996178000000
C	1.793219000000	8.202362000000	20.299945000000
C	2.212751000000	6.712290000000	20.414713000000
C	3.306216000000	6.285663000000	19.482601000000
N	3.065171000000	5.796769000000	18.198615000000
C	4.677813000000	6.273489000000	19.633019000000
C	4.258822000000	5.506236000000	17.618000000000
N	5.264252000000	5.787217000000	18.463551000000
H	1.868392000000	8.827856000000	18.240940000000
H	1.036849000000	8.412624000000	21.061619000000
H	1.320883000000	6.095332000000	20.255952000000
H	2.541731000000	6.541802000000	21.447727000000
H	5.282840000000	6.590500000000	20.464340000000
H	4.369058000000	5.115938000000	16.622404000000
H	2.126839000000	5.718897000000	17.742612000000
C	10.512712000000	0.317441000000	18.575334000000
C	10.290545000000	1.764750000000	19.036556000000
N	10.790374000000	2.763328000000	18.077511000000
C	12.071446000000	3.142406000000	17.964302000000
N	13.020952000000	2.624663000000	18.789203000000
N	12.456511000000	4.033506000000	17.034162000000
H	11.579454000000	0.103175000000	18.452103000000
H	10.013261000000	0.137560000000	17.617359000000
H	9.225767000000	1.981888000000	19.148904000000

H	10.752309000000	1.937130000000	20.015712000000
H	10.083665000000	3.193097000000	17.471591000000
H	12.821341000000	2.363879000000	19.761289000000
H	13.972048000000	2.907001000000	18.605431000000
H	11.782500000000	4.478159000000	16.393293000000
H	13.232102000000	4.655961000000	17.279547000000
Fe	7.347833000000	5.973205000000	17.969350000000
O	7.870338000000	4.368483000000	19.389131000000
O	9.122238000000	6.466423000000	17.479969000000
C	8.018012000000	5.223639000000	20.355651000000
O	7.752686000000	6.470152000000	20.090562000000
C	8.486503000000	4.804320000000	21.720441000000
C	7.707787000000	5.491316000000	22.855946000000
H	8.415322000000	3.714041000000	21.785292000000
H	9.549282000000	5.068570000000	21.818866000000
H	6.644122000000	5.225172000000	22.830621000000
H	7.790569000000	6.578108000000	22.772100000000
N	12.479921000000	8.023856000000	16.913809000000
C	12.752550000000	8.210449000000	18.390118000000
C	13.885484000000	7.229749000000	18.842364000000
O	13.981222000000	6.111933000000	18.226182000000
C	11.500057000000	7.926904000000	19.170450000000
C	10.966690000000	8.773013000000	20.288824000000
C	10.811658000000	10.290183000000	19.953716000000
N	9.674962000000	10.934772000000	20.629634000000
C	9.640085000000	11.242248000000	21.953408000000
N	10.647809000000	10.978857000000	22.787444000000
N	8.554335000000	11.854430000000	22.474141000000
O	14.562263000000	7.636368000000	19.848928000000
H	13.108545000000	9.232077000000	18.538477000000
H	9.421237000000	7.341862000000	17.796493000000
H	11.115293000000	6.912670000000	19.078638000000
H	11.607715000000	8.661112000000	21.179442000000
H	9.984114000000	8.377598000000	20.572338000000
H	10.621548000000	10.409350000000	18.883060000000
H	11.741425000000	10.828536000000	20.181755000000
H	8.974665000000	11.392265000000	20.038563000000
H	11.479575000000	10.436066000000	22.582194000000
H	10.502227000000	11.302980000000	23.764895000000
H	7.703227000000	12.016042000000	21.940531000000
H	8.599782000000	12.040093000000	23.486113000000
H	13.301180000000	8.165068000000	16.302276000000
H	11.745358000000	8.692169000000	16.552463000000
H	12.110688000000	7.069012000000	16.696417000000
O	0.661136000000	5.572267000000	17.060571000000
H	0.003191000000	4.898043000000	17.358773000000
H	0.336302000000	6.086254000000	16.286211000000
O	13.466527000000	8.564703000000	14.384929000000
H	12.495274000000	8.325054000000	14.163259000000
H	14.086018000000	8.115348000000	13.783654000000
O	10.980637000000	5.901192000000	15.790499000000
H	10.167894000000	6.039698000000	16.390147000000
H	10.912139000000	6.414534000000	14.951625000000
H	10.105704000000	-0.382636000000	19.315195000000
H	2.864257000000	-0.713940000000	21.013912000000
H	-1.407956000000	1.750665000000	19.653257000000
H	0.224338000000	2.516897000000	15.037060000000
H	5.828985000000	-2.311773000000	15.856250000000
H	7.386242000000	-2.681364000000	15.088360000000
H	-4.539635000000	6.151878000000	18.962129000000
H	-4.501055000000	6.731300000000	20.634273000000
H	2.660888000000	8.844465000000	20.481261000000
H	-3.550136000000	7.206525000000	14.756065000000
H	6.553079000000	6.210935000000	12.389123000000
H	3.559736000000	11.336326000000	17.346002000000
H	6.568522000000	16.767153000000	19.950799000000
H	9.308109000000	10.548297000000	12.572642000000
H	15.154657000000	4.358092000000	20.324655000000
H	10.563694000000	3.819881000000	24.604914000000
H	13.230990000000	13.931594000000	14.393801000000
H	8.855372000000	12.975154000000	16.993948000000
H	8.982925000000	19.694684000000	24.033779000000
H	8.111847000000	5.191579000000	23.828074000000

$^{5}TS_{reb,C3}$ – VioC WT transition state for radical rebound

C	11.411642000000	4.295749000000	25.232809000000
C	11.534572000000	5.683609000000	24.623899000000
O	11.408698000000	5.886585000000	23.388793000000
C	12.744326000000	3.521414000000	25.423038000000
O	13.407774000000	3.282935000000	24.152833000000
C	12.496267000000	2.163036000000	26.078827000000
H	10.910340000000	4.360209000000	26.208029000000
H	13.416994000000	4.118928000000	26.055353000000
H	14.105429000000	3.986662000000	23.992041000000
H	13.445445000000	1.634217000000	26.204061000000
H	12.019655000000	2.272086000000	27.059910000000
H	11.847164000000	1.552027000000	25.441310000000
N	11.792909000000	6.724270000000	25.477339000000
C	11.750632000000	8.124782000000	25.011495000000
C	12.893080000000	8.418835000000	24.025790000000
O	12.735882000000	9.201849000000	23.052566000000
C	11.759093000000	9.096824000000	26.216148000000
C	10.616545000000	8.880898000000	27.244275000000
C	10.755636000000	9.906257000000	28.389626000000
C	9.223040000000	8.983969000000	26.592161000000
H	11.769635000000	6.567082000000	26.475318000000
H	10.834375000000	8.268728000000	24.431144000000
H	12.730927000000	9.025663000000	26.726751000000
H	11.673585000000	10.115851000000	25.822668000000
H	10.724027000000	7.871021000000	27.685509000000
H	9.049756000000	8.187378000000	25.856099000000
H	9.114170000000	9.956108000000	26.094610000000
H	8.441484000000	8.899052000000	27.357160000000
H	11.756417000000	9.866246000000	28.841365000000
H	10.573036000000	10.922854000000	28.017088000000
H	10.022930000000	9.695606000000	29.178924000000
N	14.079549000000	7.807586000000	24.274353000000
C	15.117375000000	7.667649000000	23.240320000000
C	15.131244000000	6.173335000000	22.913578000000
O	15.061584000000	5.329650000000	23.876847000000
C	16.500006000000	8.237631000000	23.668521000000
C	16.427284000000	9.773044000000	23.765592000000
C	17.030544000000	7.621213000000	24.977195000000
H	14.102005000000	7.092863000000	24.991956000000
H	14.765086000000	8.223262000000	22.360074000000
H	17.194227000000	7.973987000000	22.855515000000
H	17.067200000000	6.528979000000	24.929000000000
H	18.039180000000	7.996451000000	25.186454000000
H	16.391663000000	7.904232000000	25.823076000000
H	17.406638000000	10.188644000000	24.030326000000
H	15.709823000000	10.076652000000	24.536296000000
H	16.109646000000	10.220170000000	22.817306000000
N	15.136741000000	5.817561000000	21.625934000000
C	14.839897000000	4.433151000000	21.242437000000
C	13.312218000000	4.209652000000	21.275096000000
O	12.990428000000	2.793079000000	21.449800000000
H	15.007385000000	6.525264000000	20.873304000000
H	15.331495000000	3.750071000000	21.939847000000
H	12.853746000000	4.784945000000	22.080099000000
H	12.871741000000	4.529398000000	20.331291000000
H	13.004329000000	2.619533000000	22.426491000000
C	3.756735000000	11.248339000000	16.143962000000
C	3.155693000000	9.918045000000	15.714512000000
O	2.462526000000	9.213592000000	16.502234000000
C	5.314416000000	11.257427000000	16.099098000000
C	5.950711000000	10.178133000000	16.923349000000
N	6.538371000000	10.423654000000	18.159851000000
C	6.082042000000	8.817124000000	16.714015000000
C	7.000367000000	9.249116000000	18.658409000000
N	6.746391000000	8.253006000000	17.798117000000
H	3.377590000000	12.050277000000	15.496729000000

H	5.651169000000	11.174196000000	15.058465000000
H	5.664262000000	12.234004000000	16.450947000000
H	5.760103000000	8.216950000000	15.882249000000
H	7.493347000000	9.137468000000	19.607078000000
H	6.778334000000	11.354616000000	18.579186000000
N	3.442667000000	9.510732000000	14.457602000000
C	3.185425000000	8.163160000000	13.941666000000
C	4.558425000000	7.689318000000	13.425406000000
O	5.346185000000	8.530163000000	12.905578000000
C	2.067821000000	8.172492000000	12.859436000000
O	0.846420000000	8.716872000000	13.411865000000
C	1.833381000000	6.789066000000	12.242887000000
H	4.076755000000	10.050024000000	13.877740000000
H	2.854072000000	7.545829000000	14.782040000000
H	2.365559000000	8.882726000000	12.080721000000
H	0.367474000000	8.053447000000	13.968372000000
H	0.998631000000	6.847507000000	11.537964000000
H	2.718157000000	6.432199000000	11.705134000000
H	1.582898000000	6.051476000000	13.015199000000
N	4.904834000000	6.401263000000	13.639486000000
C	6.305204000000	5.964707000000	13.484523000000
C	6.450258000000	4.454262000000	13.726242000000
C	7.821498000000	4.055267000000	14.305350000000
C	7.925143000000	4.166238000000	15.828253000000
O	8.581628000000	3.277920000000	16.445238000000
O	7.328787000000	5.191061000000	16.390687000000
H	4.234655000000	5.734446000000	14.018340000000
H	6.913616000000	6.508441000000	14.214568000000
H	6.286577000000	3.914859000000	12.782037000000
H	5.674326000000	4.122371000000	14.422797000000
H	8.067029000000	3.022481000000	14.044614000000
H	8.619574000000	4.684663000000	13.886462000000
C	6.724066000000	-1.965330000000	15.380035000000
C	7.290681000000	-0.640679000000	15.926137000000
C	8.434062000000	-0.108989000000	15.040008000000
C	6.183965000000	0.420141000000	16.085819000000
H	6.296571000000	-1.818343000000	14.378009000000
H	7.705798000000	-0.839169000000	16.927396000000
H	9.257738000000	-0.831339000000	14.976984000000
H	8.073363000000	0.071566000000	14.016870000000
H	8.825892000000	0.836305000000	15.431792000000
H	6.594991000000	1.346431000000	16.501491000000
H	5.382449000000	0.062455000000	16.745721000000
H	5.737512000000	0.657831000000	15.108571000000
C	-3.960290000000	6.054673000000	19.782571000000
C	-3.645614000000	4.639519000000	20.297695000000
C	-2.821677000000	4.613877000000	21.609356000000
C	-2.856469000000	3.212648000000	22.252281000000
C	-1.367719000000	5.075718000000	21.382775000000
H	-3.047597000000	6.623603000000	19.575730000000
H	-4.590512000000	4.099937000000	20.463782000000
H	-3.094048000000	4.083763000000	19.524071000000
H	-3.296163000000	5.314463000000	22.316031000000
H	-2.289001000000	3.191514000000	23.191163000000
H	-3.883846000000	2.895380000000	22.470104000000
H	-2.416213000000	2.462409000000	21.578789000000
H	-1.313359000000	6.091456000000	20.978925000000
H	-0.800004000000	5.050058000000	22.322234000000
H	-0.869245000000	4.413583000000	20.659252000000
C	8.726990000000	10.817978000000	13.180586000000
C	8.745353000000	12.118403000000	13.984364000000
O	8.406961000000	13.193855000000	13.432106000000
C	8.535720000000	9.478566000000	13.939736000000
C	9.786645000000	8.974533000000	14.634244000000
O	10.010785000000	9.350173000000	15.858792000000
O	10.588418000000	8.194080000000	13.982499000000
H	7.912916000000	10.928330000000	12.458434000000
H	7.736261000000	9.577987000000	14.677692000000
H	8.224723000000	8.726893000000	13.210824000000
N	9.190974000000	12.059920000000	15.278550000000
C	9.391085000000	13.266398000000	16.078735000000
C	10.858840000000	13.673131000000	16.250962000000
O	11.166726000000	14.638692000000	16.995374000000
H	9.407325000000	11.155738000000	15.700659000000
H	8.873687000000	14.088346000000	15.575181000000
N	11.767723000000	12.936436000000	15.567507000000
C	13.207365000000	13.156326000000	15.604888000000
C	13.908147000000	11.805988000000	15.477427000000
O	13.390710000000	11.173550000000	14.281612000000
H	11.464774000000	12.201979000000	14.941408000000
H	13.464522000000	13.657480000000	16.542315000000
H	13.700053000000	11.197210000000	16.370653000000
H	14.996219000000	11.953834000000	15.410861000000
H	13.417816000000	10.181433000000	14.338560000000
C	6.389505000000	15.935076000000	20.495949000000
C	6.381229000000	16.105765000000	22.014069000000
O	6.662175000000	17.214004000000	22.553144000000
C	7.407219000000	14.937754000000	19.891468000000
C	7.050845000000	13.457799000000	19.972245000000
O	7.612984000000	12.672232000000	19.099223000000
O	6.282576000000	13.014742000000	20.902122000000
H	5.377571000000	15.654580000000	20.174264000000
H	7.557291000000	15.176799000000	18.833870000000
H	8.394441000000	15.072389000000	20.355774000000
N	6.015399000000	15.017001000000	22.744752000000
C	5.712287000000	15.105843000000	24.170061000000
C	6.894429000000	15.377832000000	25.108702000000
O	6.683058000000	15.798106000000	26.267848000000
H	5.891532000000	14.141022000000	22.220740000000
H	4.998624000000	15.914798000000	24.357477000000
H	5.243146000000	14.164933000000	24.475118000000
N	8.130277000000	15.127052000000	24.596345000000
C	9.379517000000	15.396134000000	25.307590000000
C	9.977589000000	16.775165000000	24.950958000000
O	11.131937000000	17.078299000000	25.354772000000
C	10.412561000000	14.283666000000	25.031881000000
C	10.035404000000	12.951947000000	25.702844000000
O	10.159608000000	12.864624000000	26.954990000000
O	9.608740000000	11.986411000000	24.915129000000
H	8.189903000000	14.754906000000	23.657992000000
H	9.149754000000	15.407861000000	26.379008000000
H	10.529119000000	14.146852000000	23.949560000000
H	11.366012000000	14.626931000000	25.441854000000
N	9.211888000000	17.608427000000	24.198704000000
C	9.690394000000	18.918723000000	23.760686000000
C	9.642421000000	19.080083000000	22.229491000000
C	10.448010000000	18.070515000000	21.423476000000
C	11.603093000000	17.443371000000	21.922752000000
C	10.029063000000	17.750904000000	20.117615000000
C	12.310863000000	16.523799000000	21.135948000000
C	10.731817000000	16.831853000000	19.331301000000
C	11.879323000000	16.212086000000	19.842489000000
H	8.292280000000	17.321472000000	23.873767000000
H	10.703799000000	19.032219000000	24.152665000000
H	8.594083000000	19.021629000000	21.909974000000
H	9.984089000000	20.101638000000	21.995279000000
H	11.936521000000	17.634975000000	22.937607000000
H	9.133555000000	18.225590000000	19.721787000000
H	13.192901000000	16.040812000000	21.547471000000
H	10.394494000000	16.564840000000	18.335913000000
H	12.403548000000	15.488504000000	19.228167000000
C	2.665771000000	0.432536000000	20.817118000000
C	1.587376000000	0.468065000000	19.756767000000
O	1.685933000000	-0.194709000000	18.684511000000
C	4.034318000000	0.939232000000	20.280137000000
C	5.140359000000	0.668110000000	21.326665000000
C	3.958585000000	2.425904000000	19.885250000000
C	6.565513000000	0.947474000000	20.821177000000
H	2.372766000000	1.019654000000	21.697662000000
H	4.259340000000	0.346185000000	19.381243000000
H	5.073264000000	-0.383020000000	21.644930000000
H	4.941365000000	1.276806000000	22.222203000000

H	7.304834000000	0.680287000000	21.585438000000
H	6.784072000000	0.355041000000	19.923345000000
H	6.716323000000	2.002333000000	20.568507000000
H	3.168684000000	2.610507000000	19.147603000000
H	4.897126000000	2.777705000000	19.448070000000
H	3.749813000000	3.053769000000	20.761809000000
N	0.503461000000	1.264855000000	19.990732000000
C	-0.610945000000	1.357768000000	19.039096000000
C	-0.289469000000	2.267840000000	17.848352000000
O	-0.827223000000	3.409559000000	17.746833000000
H	0.477289000000	1.849658000000	20.813406000000
H	-0.848959000000	0.348633000000	18.687308000000
N	0.597549000000	1.761112000000	16.965901000000
C	1.080359000000	2.498745000000	15.792336000000
C	2.453486000000	1.965792000000	15.358935000000
C	3.094884000000	2.887301000000	14.335741000000
O	3.146738000000	4.131905000000	14.516324000000
N	3.624837000000	2.296296000000	13.232720000000
H	1.016459000000	0.860865000000	17.211306000000
H	1.168092000000	3.559344000000	16.038817000000
H	2.377804000000	0.941304000000	14.975721000000
H	3.119797000000	1.937610000000	16.231488000000
H	3.607143000000	1.298055000000	13.098422000000
H	4.110914000000	2.865285000000	12.554791000000
C	-3.054293000000	6.485582000000	15.443846000000
C	-1.738631000000	7.186653000000	15.733401000000
O	-0.709123000000	6.956006000000	15.012212000000
C	-2.859125000000	5.010591000000	15.057172000000
H	-3.725821000000	6.575064000000	16.306255000000
H	-2.420880000000	4.439546000000	15.882746000000
H	-3.820528000000	4.556237000000	14.795351000000
H	-2.189904000000	4.934782000000	14.195424000000
N	-1.717229000000	8.045975000000	16.769261000000
C	-0.535197000000	8.812800000000	17.177794000000
C	-0.142083000000	8.382140000000	18.597514000000
O	-1.015771000000	7.972881000000	19.414736000000
C	-0.779130000000	10.353373000000	17.082807000000
C	-1.009445000000	10.779428000000	15.620593000000
C	-1.921388000000	10.838409000000	17.996608000000
H	-2.491739000000	8.054017000000	17.420995000000
H	0.273132000000	8.562840000000	16.490341000000
H	0.158600000000	10.809132000000	17.428929000000
H	-0.210528000000	10.424740000000	14.960445000000
H	-1.060443000000	11.872637000000	15.548542000000
H	-1.959813000000	10.380249000000	15.243630000000
H	-1.784277000000	10.518549000000	19.034292000000
H	-1.977754000000	11.932825000000	17.978192000000
H	-2.892077000000	10.458922000000	17.650007000000
N	1.167806000000	8.526036000000	18.895544000000
C	1.717588000000	8.187921000000	20.207090000000
C	2.173778000000	6.708823000000	20.325312000000
C	3.315940000000	6.321035000000	19.435365000000
N	3.145278000000	5.832779000000	18.139765000000
C	4.680022000000	6.354701000000	19.642928000000
C	4.376514000000	5.590556000000	17.610162000000
N	5.334969000000	5.900193000000	18.496849000000
H	1.804032000000	8.815422000000	18.148892000000
H	0.944088000000	8.377957000000	20.956782000000
H	1.305431000000	6.069543000000	20.128702000000
H	2.467551000000	6.539723000000	21.369260000000
H	5.240151000000	6.686692000000	20.499669000000
H	4.545627000000	5.216675000000	16.616944000000
H	2.228460000000	5.722347000000	17.655385000000
C	10.555236000000	0.070188000000	18.863576000000
C	10.228061000000	1.505743000000	19.300338000000
N	10.507464000000	2.507006000000	18.261722000000
C	11.719374000000	3.013220000000	18.002771000000
N	12.808307000000	2.626721000000	18.716689000000
N	11.901452000000	3.917122000000	17.022513000000
H	11.618624000000	-0.034256000000	18.624355000000
H	9.976044000000	-0.204509000000	17.975517000000

H	9.164140000000	1.605996000000	19.528675000000
H	10.771203000000	1.765310000000	20.217051000000
H	9.707390000000	2.890119000000	17.738915000000
H	12.747517000000	2.314287000000	19.690451000000
H	13.685582000000	3.052453000000	18.454741000000
H	11.145429000000	4.241199000000	16.418866000000
H	12.654331000000	4.596946000000	17.153106000000
Fe	7.458843000000	6.246702000000	18.114733000000
O	8.169539000000	4.735173000000	19.485281000000
O	9.224904000000	7.036760000000	17.627891000000
C	8.066337000000	5.511680000000	20.524676000000
O	7.638373000000	6.722693000000	20.336743000000
C	8.461465000000	5.019938000000	21.892083000000
C	7.689413000000	5.694597000000	23.034734000000
H	8.333732000000	3.931699000000	21.905633000000
H	9.537022000000	5.209945000000	22.026097000000
H	6.614998000000	5.484617000000	22.971050000000
H	7.820068000000	6.779470000000	22.998452000000
N	12.075083000000	7.868410000000	16.885144000000
C	12.336511000000	7.982288000000	18.363897000000
C	13.528578000000	7.035135000000	18.744695000000
O	13.591749000000	5.912262000000	18.131305000000
C	11.116640000000	7.603033000000	19.168714000000
C	10.499556000000	8.503035000000	20.193069000000
C	10.515995000000	10.023203000000	19.865753000000
N	9.469951000000	10.795487000000	20.555415000000
C	9.491731000000	11.122094000000	21.878357000000
N	10.487695000000	10.774854000000	22.695597000000
N	8.478981000000	11.836774000000	22.406602000000
O	14.286752000000	7.477193000000	19.670126000000
H	12.644778000000	9.009612000000	18.562081000000
H	9.161557000000	7.968546000000	17.322529000000
H	10.918271000000	6.542014000000	19.258256000000
H	11.018972000000	8.328957000000	21.154132000000
H	9.468981000000	8.168132000000	20.358452000000
H	10.330199000000	10.169202000000	18.797135000000
H	11.504015000000	10.450280000000	20.085303000000
H	8.825757000000	11.340529000000	19.975671000000
H	11.306641000000	10.222212000000	22.461594000000
H	10.396340000000	11.127337000000	23.667535000000
H	7.628466000000	12.053435000000	21.890134000000
H	8.582386000000	12.071259000000	23.406304000000
H	12.895743000000	8.071713000000	16.288193000000
H	11.311899000000	8.529588000000	16.568404000000
H	11.742793000000	6.918041000000	16.617231000000
O	0.734690000000	5.552585000000	16.975703000000
H	0.073066000000	4.888007000000	17.286918000000
H	0.405942000000	6.043567000000	16.188565000000
O	13.153464000000	8.469440000000	14.425279000000
H	12.169233000000	8.345863000000	14.183200000000
H	13.730394000000	7.942933000000	13.844919000000
O	10.530186000000	5.877623000000	15.668002000000
H	9.899409000000	6.231633000000	16.380592000000
H	10.488778000000	6.444978000000	14.864039000000
H	10.314179000000	-0.635959000000	19.667365000000
H	2.772693000000	-0.611468000000	21.138147000000
H	-1.475900000000	1.775971000000	19.551960000000
H	0.359098000000	2.406255000000	14.968689000000
H	5.931590000000	-2.359160000000	16.028860000000
H	7.506748000000	-2.729663000000	15.298453000000
H	-4.556338000000	6.006714000000	18.860998000000
H	-4.544173000000	6.618325000000	20.522263000000
H	2.565843000000	8.851496000000	20.401947000000
H	-3.531426000000	7.023828000000	14.611957000000
H	6.663569000000	6.244772000000	12.487940000000
H	3.410244000000	11.443058000000	17.161601000000
H	6.602731000000	16.929313000000	20.095782000000
H	9.656820000000	10.778465000000	12.594879000000
H	15.242266000000	4.265962000000	20.240391000000
H	10.775320000000	3.710792000000	24.562196000000
H	13.518943000000	13.806991000000	14.776369000000

H 8.953641000000 13.145720000000 17.076917000000
H 9.073013000000 19.706165000000 24.214464000000
H 8.049148000000 5.335435000000 24.005269000000

[5]P_{C3} – VioC WT product bound state

C 11.472712000000 4.312122000000 25.449259000000
C 11.554598000000 5.688606000000 24.810690000000
O 11.419730000000 5.860027000000 23.572714000000
C 12.813845000000 3.533808000000 25.550104000000
O 13.392709000000 3.289181000000 24.239849000000
C 12.604191000000 2.177749000000 26.223842000000
H 11.045237000000 4.389818000000 26.457816000000
H 13.527033000000 4.130953000000 26.136712000000
H 14.079464000000 3.991398000000 24.040832000000
H 13.558617000000 1.648048000000 26.293647000000
H 12.186939000000 2.289939000000 27.231330000000
H 11.917650000000 1.566059000000 25.627502000000
N 11.789613000000 6.758836000000 25.634690000000
C 11.702745000000 8.142029000000 25.124275000000
C 12.834096000000 8.445040000000 24.129327000000
O 12.662717000000 9.233392000000 23.164556000000
C 11.668897000000 9.152355000000 26.295492000000
C 10.515422000000 8.937458000000 27.311556000000
C 10.615068000000 9.991744000000 28.434261000000
C 9.131381000000 8.997084000000 26.634115000000
H 11.771815000000 6.630941000000 26.636916000000
H 10.785455000000 8.235187000000 24.534901000000
H 12.634206000000 9.123517000000 26.822654000000
H 11.562029000000 10.155043000000 25.867011000000
H 10.635108000000 7.940273000000 27.777331000000
H 8.983756000000 8.175888000000 25.920047000000
H 9.015054000000 9.951279000000 26.104864000000
H 8.338388000000 8.922146000000 27.388311000000
H 11.609412000000 9.983717000000 28.901782000000
H 10.416659000000 10.994446000000 28.033751000000
H 9.874690000000 9.784273000000 29.217327000000
N 14.027026000000 7.835062000000 24.358453000000
C 15.044257000000 7.702724000000 23.303383000000
C 15.043865000000 6.214319000000 22.953137000000
O 15.040727000000 5.355027000000 23.904340000000
C 16.438034000000 8.260005000000 23.712352000000
C 16.376399000000 9.795262000000 23.821412000000
C 16.986724000000 7.632179000000 25.008013000000
H 14.061887000000 7.119857000000 25.074605000000
H 14.677138000000 8.275879000000 22.446986000000
H 17.117549000000 7.997758000000 22.886374000000
H 17.013803000000 6.540309000000 24.951818000000
H 18.001303000000 7.999636000000 25.202130000000
H 16.363946000000 7.914983000000 25.865888000000
H 17.361424000000 10.202900000000 24.077389000000
H 15.669790000000 10.097196000000 24.602665000000
H 16.050122000000 10.252136000000 22.880610000000
N 14.969314000000 5.876873000000 21.661672000000
C 14.671323000000 4.493417000000 21.277055000000
C 13.152086000000 4.258534000000 21.386957000000
O 12.849227000000 2.828516000000 21.463401000000
H 14.758823000000 6.575842000000 20.923715000000
H 15.204101000000 3.809047000000 21.941988000000
H 12.744652000000 4.761486000000 22.262555000000
H 12.643746000000 4.650562000000 20.506867000000
H 12.898814000000 2.583556000000 22.419931000000
C 3.825764000000 11.240320000000 16.194354000000
C 3.215897000000 9.913247000000 15.766892000000
O 2.532939000000 9.205270000000 16.559883000000
C 5.380390000000 11.261505000000 16.090408000000
C 6.049117000000 10.178259000000 16.882197000000
N 6.599277000000 10.405090000000 18.140152000000
C 6.229706000000 8.830034000000 16.635448000000
C 7.081633000000 9.230418000000 18.616878000000
N 6.882618000000 8.251134000000 17.720177000000
H 3.415110000000 12.049443000000 15.575679000000
H 5.679362000000 11.188031000000 15.037579000000
H 5.736647000000 12.236763000000 16.440168000000
H 5.952793000000 8.251818000000 15.772733000000
H 7.549556000000 9.102029000000 19.576304000000
H 6.808567000000 11.340121000000 18.567818000000
N 3.478347000000 9.516965000000 14.500824000000
C 3.200469000000 8.178597000000 13.974248000000
C 4.561420000000 7.691534000000 13.438198000000
O 5.367814000000 8.529816000000 12.943116000000
C 2.069322000000 8.214638000000 12.905621000000
O 0.864398000000 8.773561000000 13.478457000000
C 1.804782000000 6.841619000000 12.278030000000
H 4.109874000000 10.056174000000 13.918689000000
H 2.871708000000 7.555856000000 14.811696000000
H 2.368954000000 8.927637000000 12.129962000000
H 0.375133000000 8.110751000000 14.026820000000
H 0.962057000000 6.921134000000 11.584740000000
H 2.676392000000 6.476198000000 11.724727000000
H 1.551965000000 6.099500000000 13.045215000000
N 4.882437000000 6.390328000000 13.609441000000
C 6.273187000000 5.933379000000 13.429616000000
C 6.400491000000 4.427233000000 13.703171000000
C 7.800763000000 4.006620000000 14.203385000000
C 7.992021000000 4.176149000000 15.712590000000
O 8.329808000000 3.163533000000 16.394839000000
O 7.839291000000 5.391441000000 16.199371000000
H 4.197670000000 5.720627000000 13.955998000000
H 6.907476000000 6.485356000000 14.130957000000
H 6.166330000000 3.866598000000 12.787404000000
H 5.659713000000 4.125830000000 14.451424000000
H 7.980705000000 2.953540000000 13.974772000000
H 8.579940000000 4.592882000000 13.698154000000
C 6.686351000000 -2.136340000000 15.557936000000
C 7.188586000000 -0.746277000000 15.992492000000
C 8.358788000000 -0.268126000000 15.111132000000
C 6.046846000000 0.289391000000 15.993067000000
H 6.305284000000 -2.104485000000 14.527409000000
H 7.559912000000 -0.829229000000 17.026750000000
H 9.201646000000 -0.969630000000 15.152980000000
H 8.042335000000 -0.192826000000 14.060577000000
H 8.708038000000 0.719124000000 15.432566000000
H 6.415588000000 1.266663000000 16.323151000000
H 5.227723000000 -0.022902000000 16.654316000000
H 5.636601000000 0.406617000000 14.978462000000
C -3.877285000000 6.042776000000 19.897527000000
C -3.576187000000 4.615027000000 20.385172000000
C -2.732178000000 4.555632000000 21.682895000000
C -2.777483000000 3.144935000000 22.304253000000
C -1.275327000000 5.000450000000 21.441588000000
H -2.959433000000 6.600516000000 19.683195000000
H -4.526368000000 4.086903000000 20.557761000000
H -3.045815000000 4.064158000000 19.593336000000
H -3.185313000000 5.251330000000 22.408102000000
H -2.197159000000 3.101343000000 23.234427000000
H -3.805986000000 2.838465000000 22.531951000000
H -2.357542000000 2.398991000000 21.613157000000
H -1.212293000000 6.020298000000 21.049654000000
H -0.694811000000 4.954717000000 22.372365000000
H -0.795610000000 4.341440000000 20.702520000000
C 8.815023000000 11.132600000000 13.104866000000
C 8.954854000000 12.412456000000 13.929980000000
O 8.723915000000 13.524625000000 13.396789000000
C 8.528726000000 9.805368000000 13.855369000000
C 9.758280000000 9.193552000000 14.498638000000
O 10.037825000000 9.504390000000 15.729068000000
O 10.497810000000 8.397365000000 13.791845000000
H 8.001481000000 11.323772000000 12.398860000000
H 7.772043000000 9.964626000000 14.626758000000
H 8.123333000000 9.088612000000 13.136999000000
N 9.379914000000 12.285565000000 15.225743000000
C 9.656481000000 13.447051000000 16.067383000000
C 11.146946000000 13.713185000000 16.304296000000

O	11.511081000000	14.612241000000	17.104129000000
H	9.521284000000	11.358301000000	15.626013000000
H	9.232242000000	14.325064000000	15.571287000000
N	12.011457000000	12.932598000000	15.611765000000
C	13.462844000000	13.024916000000	15.702663000000
C	14.051243000000	11.626413000000	15.534606000000
O	13.522225000000	11.093868000000	14.296114000000
H	11.667381000000	12.257128000000	14.942068000000
H	13.729418000000	13.461241000000	16.669370000000
H	13.763070000000	10.999408000000	16.392403000000
H	15.149524000000	11.683596000000	15.507614000000
H	13.485217000000	10.100680000000	14.296533000000
C	6.369168000000	15.985379000000	20.390722000000
C	6.333665000000	16.148996000000	21.909456000000
O	6.613726000000	17.252633000000	22.458229000000
C	7.394216000000	14.987909000000	19.799020000000
C	7.032649000000	13.508268000000	19.868886000000
O	7.604020000000	12.723978000000	19.000263000000
O	6.250291000000	13.064141000000	20.786093000000
H	5.362404000000	15.709921000000	20.049173000000
H	7.559545000000	15.230466000000	18.744396000000
H	8.373996000000	15.118620000000	20.280124000000
N	5.943627000000	15.060718000000	22.628015000000
C	5.612376000000	15.146733000000	24.047255000000
C	6.778312000000	15.397760000000	25.012013000000
O	6.548164000000	15.813542000000	26.169119000000
H	5.828583000000	14.186407000000	22.099423000000
H	4.906546000000	15.964988000000	24.223371000000
H	5.123494000000	14.211245000000	24.337593000000
N	8.020924000000	15.135519000000	24.522512000000
C	9.260149000000	15.386523000000	25.258194000000
C	9.870945000000	16.767440000000	24.934747000000
O	11.015041000000	17.061550000000	25.373074000000
C	10.288588000000	14.271058000000	24.979006000000
C	9.897053000000	12.938198000000	25.640788000000
O	9.962214000000	12.865872000000	26.898664000000
O	9.521211000000	11.960572000000	24.843684000000
H	8.094378000000	14.768901000000	23.582893000000
H	9.015784000000	15.379888000000	26.326332000000
H	10.413463000000	14.141556000000	23.896817000000
H	11.241565000000	14.604251000000	25.399004000000
N	9.128966000000	17.612285000000	24.171498000000
C	9.625163000000	18.923358000000	23.756891000000
C	9.640321000000	19.088438000000	22.225085000000
C	10.475291000000	18.077434000000	21.451403000000
C	11.609140000000	17.449630000000	21.996469000000
C	10.107885000000	17.756771000000	20.130402000000
C	12.346459000000	16.528291000000	21.239576000000
C	10.841621000000	16.836874000000	19.373931000000
C	11.967351000000	16.215926000000	19.930033000000
H	8.216670000000	17.333402000000	23.820539000000
H	10.621912000000	19.035424000000	24.190009000000
H	8.605635000000	19.034372000000	21.862964000000
H	9.994504000000	20.109416000000	22.007273000000
H	11.901082000000	17.640647000000	23.024062000000
H	9.229215000000	18.231570000000	19.698474000000
H	13.210410000000	16.044489000000	21.686893000000
H	10.546340000000	16.572647000000	18.364569000000
H	12.514344000000	15.492041000000	19.336601000000
C	2.594152000000	0.314331000000	20.791321000000
C	1.528441000000	0.402908000000	19.721330000000
O	1.617734000000	-0.243248000000	18.638121000000
C	3.983857000000	0.789691000000	20.281111000000
C	5.068603000000	0.445011000000	21.328721000000
C	3.961665000000	2.289664000000	19.931845000000
C	6.507598000000	0.710085000000	20.856298000000
H	2.309058000000	0.891086000000	21.681173000000
H	4.197012000000	0.217343000000	19.366026000000
H	4.970452000000	-0.617249000000	21.597935000000
H	4.872779000000	1.017709000000	22.248330000000
H	7.229592000000	0.378416000000	21.611410000000
H	6.720708000000	0.166461000000	19.926780000000
H	6.688717000000	1.774059000000	20.670956000000
H	3.180948000000	2.523576000000	19.198288000000
H	4.912835000000	2.621487000000	19.506230000000
H	3.772172000000	2.898105000000	20.826160000000
N	0.465279000000	1.225274000000	19.961150000000
C	-0.643003000000	1.359276000000	19.007643000000
C	-0.298945000000	2.273956000000	17.826666000000
O	-0.815755000000	3.425697000000	17.733957000000
H	0.447599000000	1.794937000000	20.794706000000
H	-0.906720000000	0.360955000000	18.643152000000
N	0.578345000000	1.755491000000	16.940690000000
C	1.065296000000	2.481523000000	15.762656000000
C	2.467165000000	1.986146000000	15.373738000000
C	3.067865000000	2.871440000000	14.296430000000
O	3.130991000000	4.120695000000	14.437002000000
N	3.537606000000	2.246686000000	13.184661000000
H	0.977319000000	0.844329000000	17.179896000000
H	1.112296000000	3.549396000000	15.986889000000
H	2.441186000000	0.937046000000	15.056495000000
H	3.124817000000	2.045788000000	16.250302000000
H	3.514585000000	1.244716000000	13.082984000000
H	3.977519000000	2.794914000000	12.459870000000
C	-3.051942000000	6.569723000000	15.532682000000
C	-1.719341000000	7.242928000000	15.809922000000
O	-0.710732000000	7.016759000000	15.057928000000
C	-2.888628000000	5.098643000000	15.116672000000
H	-3.705899000000	6.655014000000	16.408860000000
H	-2.445983000000	4.505553000000	15.924191000000
H	-3.861983000000	4.665168000000	14.863821000000
H	-2.235819000000	5.027245000000	14.242054000000
N	-1.659529000000	8.070783000000	16.869215000000
C	-0.455921000000	8.805660000000	17.273937000000
C	-0.046581000000	8.330225000000	18.674656000000
O	-0.913602000000	7.908976000000	19.492960000000
C	-0.672649000000	10.352179000000	17.223175000000
C	-0.920231000000	10.820630000000	15.776832000000
C	-1.789290000000	10.833345000000	18.169943000000
H	-2.417987000000	8.071359000000	17.539734000000
H	0.335304000000	8.558785000000	16.565825000000
H	0.279437000000	10.781264000000	17.563992000000
H	-0.141266000000	10.465804000000	15.093254000000
H	-0.948086000000	11.916166000000	15.733590000000
H	-1.885874000000	10.452429000000	15.407164000000
H	-1.637153000000	10.487553000000	19.197188000000
H	-1.828547000000	11.928621000000	18.177896000000
H	-2.772349000000	10.476849000000	17.834123000000
N	1.269358000000	8.447551000000	18.956204000000
C	1.834722000000	8.063840000000	20.249003000000
C	2.270407000000	6.575382000000	20.317168000000
C	3.390932000000	6.199472000000	19.395385000000
N	3.191982000000	5.772717000000	18.082408000000
C	4.758555000000	6.201786000000	19.582489000000
C	4.411625000000	5.539414000000	17.522694000000
N	5.391739000000	5.793335000000	18.404660000000
H	1.897419000000	8.753914000000	18.209845000000
H	1.076378000000	8.244223000000	21.016318000000
H	1.388792000000	5.954640000000	20.119421000000
H	2.581604000000	6.372869000000	21.349914000000
H	5.340763000000	6.484031000000	20.443185000000
H	4.545941000000	5.208877000000	16.508596000000
H	2.267293000000	5.691569000000	17.607893000000
C	10.770714000000	0.072156000000	18.871798000000
C	10.229211000000	1.466832000000	19.219057000000
N	10.400524000000	2.438096000000	18.129826000000
C	11.522582000000	3.140902000000	17.918070000000
N	12.624515000000	2.980124000000	18.684559000000
N	11.599695000000	4.031361000000	16.910650000000
H	11.845579000000	0.108317000000	18.664784000000
H	10.266003000000	-0.328869000000	17.986103000000
H	9.154831000000	1.420546000000	19.416045000000

H	10.700260000000	1.846527000000	20.134076000000
H	9.561595000000	2.733699000000	17.614853000000
H	12.599364000000	2.552998000000	19.611947000000
H	13.375291000000	3.641943000000	18.518626000000
H	10.839107000000	4.202543000000	16.269857000000
H	12.313504000000	4.759983000000	16.987736000000
Fe	7.510367000000	6.261954000000	18.099125000000
O	8.528844000000	4.936263000000	19.385436000000
O	9.656926000000	7.584867000000	17.978070000000
C	8.265598000000	5.561999000000	20.504240000000
O	7.653365000000	6.695364000000	20.456971000000
C	8.699343000000	4.949826000000	21.814582000000
C	7.822992000000	5.365554000000	23.003620000000
H	8.715989000000	3.861426000000	21.686240000000
H	9.736932000000	5.253168000000	22.014787000000
H	6.787321000000	5.027784000000	22.875454000000
H	7.807362000000	6.454266000000	23.103770000000
N	11.933983000000	7.785457000000	16.677125000000
C	12.144188000000	7.921532000000	18.152545000000
C	13.258928000000	6.967884000000	18.645180000000
O	13.410652000000	5.855797000000	18.014758000000
C	10.795127000000	7.635506000000	18.913391000000
C	10.547520000000	8.582776000000	20.103870000000
C	10.502408000000	10.101145000000	19.758669000000
N	9.445431000000	10.856364000000	20.454448000000
C	9.450754000000	11.149286000000	21.785050000000
N	10.427878000000	10.769683000000	22.610196000000
N	8.438604000000	11.865307000000	22.314407000000
O	13.892724000000	7.376072000000	19.674279000000
H	12.477034000000	8.945455000000	18.336101000000
H	9.478539000000	8.433901000000	17.514835000000
H	10.816274000000	6.608794000000	19.283610000000
H	11.334054000000	8.389359000000	20.843000000000
H	9.596428000000	8.282875000000	20.554190000000
H	10.296496000000	10.248738000000	18.692698000000
H	11.480156000000	10.562087000000	19.954945000000
H	8.817008000000	11.424670000000	19.879841000000
H	11.238874000000	10.206755000000	22.382388000000
H	10.328344000000	11.097646000000	23.588110000000
H	7.595085000000	12.094278000000	21.792699000000
H	8.527979000000	12.071534000000	23.322256000000
H	12.801181000000	7.842723000000	16.122217000000
H	11.302852000000	8.553281000000	16.319231000000
H	11.435946000000	6.909403000000	16.394986000000
O	0.762924000000	5.543922000000	16.939254000000
H	0.097769000000	4.889068000000	17.264003000000
H	0.414814000000	6.060828000000	16.177280000000
O	13.093376000000	8.405717000000	14.238167000000
H	12.106759000000	8.401873000000	13.991345000000
H	13.620992000000	7.905928000000	13.591079000000
O	10.352152000000	6.041019000000	15.252770000000
H	9.450668000000	6.040068000000	15.663700000000
H	10.385414000000	6.698121000000	14.513894000000
H	10.607964000000	-0.618720000000	19.707686000000
H	2.665362000000	-0.739106000000	21.090891000000
H	-1.498110000000	1.793438000000	19.523677000000
H	0.368836000000	2.344255000000	14.923716000000
H	5.873741000000	-2.488684000000	16.205698000000
H	7.492877000000	-2.879224000000	15.591326000000
H	-4.489718000000	6.018312000000	18.985826000000
H	-4.439567000000	6.603636000000	20.655816000000
H	2.696067000000	8.708735000000	20.448607000000
H	-3.534724000000	7.130374000000	14.719145000000
H	6.614528000000	6.181470000000	12.418141000000
H	3.518352000000	11.416616000000	17.227787000000
H	6.592817000000	16.981057000000	19.999702000000
H	9.726789000000	11.023999000000	12.500368000000
H	15.027754000000	4.340034000000	20.255506000000
H	10.787324000000	3.720003000000	24.835187000000
H	13.856295000000	13.681355000000	14.914559000000
H	9.170610000000	13.336181000000	17.044083000000
H	8.989602000000	19.709546000000	24.186898000000
H	8.209339000000	4.936269000000	23.934940000000

VioC MeHis316

$^{5}Re_{Me}$ – VioC MeHis Reactant state

C	11.236822000000	5.181683000000	25.920412000000
C	11.492137000000	6.534536000000	25.277240000000
O	11.297952000000	6.740478000000	24.054515000000
C	12.430705000000	4.187780000000	25.909505000000
O	12.883186000000	3.901690000000	24.559054000000
C	12.037086000000	2.860550000000	26.556587000000
H	10.910347000000	5.313086000000	26.960731000000
H	13.268670000000	4.634332000000	26.464649000000
H	13.632027000000	4.524372000000	24.322405000000
H	12.892906000000	2.179447000000	26.555093000000
H	11.699429000000	3.002838000000	27.589688000000
H	11.227250000000	2.391931000000	25.986019000000
N	11.947828000000	7.547942000000	26.090906000000
C	12.000596000000	8.936123000000	25.598123000000
C	13.179233000000	9.171638000000	24.637417000000
O	13.157666000000	10.133740000000	23.821867000000
C	11.988599000000	9.954289000000	26.761161000000
C	10.818327000000	9.800420000000	27.769161000000
C	10.865564000000	10.966061000000	28.779163000000
C	9.433225000000	9.729237000000	27.095946000000
H	11.983434000000	7.401904000000	27.090028000000
H	11.113203000000	9.098780000000	24.976731000000
H	12.944038000000	9.899859000000	27.304626000000
H	11.937172000000	10.952244000000	26.310571000000
H	10.976954000000	8.865935000000	28.338792000000
H	9.347384000000	8.865726000000	26.424113000000
H	9.214444000000	10.643618000000	26.526612000000
H	8.654954000000	9.623670000000	27.862132000000
H	11.850725000000	11.050904000000	29.256263000000
H	10.640887000000	11.912132000000	28.273488000000
H	10.116329000000	10.826616000000	29.566722000000
N	14.211968000000	8.297923000000	24.702312000000
C	15.125294000000	8.055840000000	23.570953000000
C	14.867754000000	6.591523000000	23.203522000000
O	14.732793000000	5.747264000000	24.159736000000
C	16.615178000000	8.377568000000	23.883255000000
C	16.801350000000	9.898294000000	24.043853000000
C	17.152767000000	7.617868000000	25.110381000000
H	14.114750000000	7.504118000000	25.325803000000
H	14.798659000000	8.704482000000	22.753039000000
H	17.181518000000	8.050761000000	22.997234000000
H	17.020953000000	6.536545000000	25.012549000000
H	18.220225000000	7.829343000000	25.243603000000
H	16.633619000000	7.942622000000	26.020452000000
H	17.856634000000	10.138580000000	24.217976000000
H	16.221727000000	10.270455000000	24.896141000000
H	16.467823000000	10.440712000000	23.152549000000
N	14.720434000000	6.257664000000	21.919972000000
C	14.200143000000	4.933973000000	21.558348000000
C	12.660231000000	4.919650000000	21.690474000000
O	12.173972000000	3.547205000000	21.859815000000
H	14.715842000000	6.935299000000	21.137435000000
H	14.634075000000	4.181127000000	22.221465000000
H	12.334897000000	5.525779000000	22.537464000000
H	12.194537000000	5.313423000000	20.787302000000
H	12.256718000000	3.337451000000	22.825497000000
C	3.662112000000	11.039466000000	17.518431000000
C	2.961333000000	9.892579000000	16.812742000000
O	2.277079000000	9.029203000000	17.436723000000
C	5.212559000000	11.008150000000	17.368022000000
C	5.862947000000	9.776567000000	17.932930000000
N	6.726393000000	9.844249000000	19.024796000000
C	5.770255000000	8.433826000000	17.615250000000
C	7.120429000000	8.591090000000	19.351991000000
N	6.541106000000	7.709261000000	18.521710000000
H	3.296011000000	11.994905000000	17.120301000000
H	5.481674000000	11.120878000000	16.309451000000
H	5.598520000000	11.889901000000	17.893085000000
H	5.200472000000	7.941869000000	16.847518000000
H	7.787476000000	8.331883000000	20.154398000000
H	7.125195000000	10.740910000000	19.417441000000
N	3.160399000000	9.815500000000	15.476657000000
C	2.833882000000	8.654681000000	14.640869000000
C	4.194965000000	8.222168000000	14.071184000000
O	4.957399000000	9.088996000000	13.563575000000
C	1.788190000000	9.013829000000	13.551922000000
O	0.562660000000	9.458258000000	14.169581000000
C	1.532874000000	7.849923000000	12.586014000000
H	3.761144000000	10.492688000000	15.020890000000
H	2.412309000000	7.885757000000	15.293687000000
H	2.166463000000	9.878572000000	12.996787000000
H	0.040407000000	8.691422000000	14.522464000000
H	0.762392000000	8.142243000000	11.866685000000
H	2.440396000000	7.585309000000	12.029545000000
H	1.169831000000	6.971226000000	13.133118000000
N	4.544284000000	6.923746000000	14.235956000000
C	5.934298000000	6.469054000000	14.056810000000
C	6.010413000000	4.935129000000	13.986188000000
C	7.340665000000	4.377816000000	14.511753000000
C	7.440555000000	4.220519000000	16.029507000000
O	8.340976000000	3.464999000000	16.484026000000
O	6.583307000000	4.901585000000	16.774436000000
H	3.883220000000	6.273175000000	14.659028000000
H	6.531789000000	6.831006000000	14.902590000000
H	5.857284000000	4.604685000000	12.950610000000
H	5.199724000000	4.504589000000	14.582526000000
H	7.553037000000	3.393719000000	14.081352000000
H	8.181732000000	5.025504000000	14.221554000000
C	6.237856000000	-1.975038000000	15.069163000000
C	6.534284000000	-0.571144000000	15.631505000000
C	7.846682000000	-0.002285000000	15.058723000000
C	5.352562000000	0.385501000000	15.373952000000
H	6.111549000000	-1.935283000000	13.978336000000
H	6.655957000000	-0.663383000000	16.722899000000
H	8.690896000000	-0.672656000000	15.263678000000
H	7.772377000000	0.111861000000	13.967833000000
H	8.080735000000	0.978315000000	15.489357000000
H	5.551836000000	1.373865000000	15.806642000000
H	4.424557000000	-0.004759000000	15.810839000000
H	5.195534000000	0.513684000000	14.293052000000
C	-4.079581000000	4.587411000000	19.625016000000
C	-3.735110000000	3.091891000000	19.731103000000
C	-2.803286000000	2.742156000000	20.917758000000
C	-2.748776000000	1.216751000000	21.135512000000
C	-1.384907000000	3.313888000000	20.718885000000
H	-3.183385000000	5.207080000000	19.508510000000
H	-4.668859000000	2.518894000000	19.837898000000
H	-3.261055000000	2.750002000000	18.799215000000
H	-3.229458000000	3.194732000000	21.828178000000
H	-2.097857000000	0.955639000000	21.979823000000
H	-3.745023000000	0.804498000000	21.336989000000
H	-2.362217000000	0.713944000000	20.236466000000
H	-1.393019000000	4.404385000000	20.620756000000
H	-0.736429000000	3.054514000000	21.566483000000
H	-0.939218000000	2.899708000000	19.802862000000
C	8.586310000000	11.607403000000	13.391310000000
C	9.294625000000	12.898297000000	13.804560000000
O	9.184277000000	13.926828000000	13.093555000000
C	8.221127000000	10.581790000000	14.498033000000
C	9.407808000000	9.758288000000	14.971239000000
O	10.115520000000	10.205877000000	15.952452000000
O	9.685080000000	8.655297000000	14.338942000000
H	7.671567000000	11.924433000000	12.881916000000
H	7.791340000000	11.102806000000	15.359912000000
H	7.465937000000	9.901281000000	14.093875000000
N	10.072245000000	12.858468000000	14.932042000000
C	10.828184000000	14.018770000000	15.386215000000
C	12.345068000000	13.817249000000	15.405077000000
O	13.075071000000	14.552130000000	16.119347000000

H	10.107180000000	12.006316000000	15.497718000000
H	10.587942000000	14.848096000000	14.711969000000
N	12.824287000000	12.838954000000	14.600109000000
C	14.232185000000	12.475710000000	14.495815000000
C	14.353993000000	10.953414000000	14.491223000000
O	13.507679000000	10.457324000000	13.428201000000
H	12.190740000000	12.268221000000	14.055098000000
H	14.767088000000	12.922994000000	15.338072000000
H	14.037294000000	10.558274000000	15.468524000000
H	15.403730000000	10.664196000000	14.331160000000
H	13.116796000000	9.570448000000	13.658515000000
C	7.997737000000	15.783767000000	19.172758000000
C	7.444543000000	16.419392000000	20.440562000000
O	7.854866000000	17.536826000000	20.857828000000
C	8.582148000000	14.369378000000	19.364091000000
C	7.545900000000	13.250048000000	19.468890000000
O	8.012357000000	12.057640000000	19.752901000000
O	6.309606000000	13.492167000000	19.283042000000
H	7.201109000000	15.735036000000	18.420079000000
H	9.223191000000	14.126886000000	18.505227000000
H	9.242161000000	14.327357000000	20.239129000000
N	6.492641000000	15.708057000000	21.114055000000
C	5.812894000000	16.254707000000	22.288486000000
C	6.695051000000	16.549639000000	23.511832000000
O	6.289143000000	17.365047000000	24.368736000000
H	6.122147000000	14.880543000000	20.646490000000
H	5.326705000000	17.206638000000	22.050561000000
H	5.036694000000	15.543182000000	22.587758000000
N	7.883406000000	15.888453000000	23.589713000000
C	8.861574000000	16.114114000000	24.659757000000
C	9.860994000000	17.242159000000	24.329891000000
O	10.834174000000	17.456032000000	25.104277000000
C	9.638426000000	14.824848000000	24.969511000000
C	8.790783000000	13.747950000000	25.673323000000
O	7.951833000000	14.094214000000	26.540805000000
O	9.047360000000	12.502781000000	25.304911000000
H	8.109808000000	15.212645000000	22.872315000000
H	8.302639000000	16.401119000000	25.556679000000
H	10.084491000000	14.418075000000	24.053509000000
H	10.467342000000	15.104295000000	25.630522000000
N	9.665086000000	17.940882000000	23.183042000000
C	10.589164000000	18.982451000000	22.736405000000
C	11.037959000000	18.781039000000	21.278296000000
C	11.814332000000	17.507840000000	20.966673000000
C	12.446233000000	16.732594000000	21.954077000000
C	11.927947000000	17.096972000000	19.623659000000
C	13.169756000000	15.584569000000	21.601210000000
C	12.653893000000	15.956014000000	19.264023000000
C	13.278935000000	15.195269000000	20.262097000000
H	8.875480000000	17.736863000000	22.576216000000
H	11.432326000000	18.980922000000	23.431789000000
H	10.147813000000	18.810307000000	20.636686000000
H	11.650124000000	19.653925000000	20.999497000000
H	12.364704000000	17.003595000000	23.001849000000
H	11.442651000000	17.687507000000	18.849237000000
H	13.643842000000	14.994371000000	22.380528000000
H	12.736263000000	15.660317000000	18.221075000000
H	13.844433000000	14.307991000000	19.992141000000
C	2.584781000000	-0.593077000000	19.532108000000
C	1.655382000000	-0.470893000000	18.340165000000
O	2.039472000000	-0.692133000000	17.156295000000
C	3.966610000000	0.070856000000	19.311290000000
C	4.900334000000	-0.254366000000	20.501509000000
C	3.823679000000	1.586522000000	19.082852000000
C	6.360564000000	0.180904000000	20.293509000000
H	2.111382000000	-0.175646000000	20.431620000000
H	4.395846000000	-0.376615000000	18.404028000000
H	4.873672000000	-1.339393000000	20.683157000000
H	4.499870000000	0.221312000000	21.410374000000
H	6.979656000000	-0.131003000000	21.143112000000
H	6.779843000000	-0.280378000000	19.389912000000
H	6.461067000000	1.266784000000	20.189911000000
H	3.183501000000	1.794108000000	18.217317000000
H	4.792594000000	2.057710000000	18.895882000000
H	3.382030000000	2.074805000000	19.962535000000
N	0.359661000000	-0.131154000000	18.604707000000
C	-0.677944000000	-0.110985000000	17.564348000000
C	-0.654707000000	1.160088000000	16.707605000000
O	-1.553875000000	2.033489000000	16.796728000000
H	0.089169000000	0.114839000000	19.546054000000
H	-0.543968000000	-0.992057000000	16.927539000000
N	0.406337000000	1.265103000000	15.865851000000
C	0.605780000000	2.467106000000	15.055290000000
C	1.996451000000	2.450914000000	14.392195000000
C	2.442233000000	3.855909000000	14.027851000000
O	2.654707000000	4.718045000000	14.926113000000
N	2.607063000000	4.140690000000	12.713700000000
H	1.126833000000	0.544666000000	15.922795000000
H	0.507879000000	3.349021000000	15.695328000000
H	2.005749000000	1.785572000000	13.521987000000
H	2.733982000000	2.066779000000	15.107396000000
H	2.440964000000	3.459441000000	11.990545000000
H	2.902113000000	5.069620000000	12.444611000000
C	-3.018180000000	6.149448000000	15.530124000000
C	-1.830866000000	7.010650000000	15.938170000000
O	-0.934453000000	7.315099000000	15.098356000000
C	-2.601476000000	4.887102000000	14.756549000000
H	-3.617401000000	5.880735000000	16.407948000000
H	-2.126586000000	4.153415000000	15.416518000000
H	-3.479424000000	4.409822000000	14.307751000000
H	-1.905129000000	5.157089000000	13.956395000000
N	-1.820466000000	7.439724000000	17.223814000000
C	-0.768025000000	8.266203000000	17.816288000000
C	-0.355780000000	7.613592000000	19.140812000000
O	-1.161028000000	6.877016000000	19.783804000000
C	-1.216450000000	9.750552000000	18.047225000000
C	-1.490425000000	10.461325000000	16.709663000000
C	-2.427201000000	9.860704000000	18.993834000000
H	-2.480877000000	7.050071000000	17.885450000000
H	0.083017000000	8.275841000000	17.131337000000
H	-0.355240000000	10.239783000000	18.524121000000
H	-0.626355000000	10.425832000000	16.038832000000
H	-1.743157000000	11.513240000000	16.890013000000
H	-2.341829000000	10.002081000000	16.192095000000
H	-2.255188000000	9.359008000000	19.951731000000
H	-2.650259000000	10.914586000000	19.195572000000
H	-3.320766000000	9.417705000000	18.535977000000
N	0.891251000000	7.903755000000	19.577151000000
C	1.373942000000	7.479521000000	20.892082000000
C	1.849227000000	6.001998000000	20.961725000000
C	2.974451000000	5.669915000000	20.031820000000
N	2.797956000000	5.143701000000	18.741610000000
C	4.332989000000	5.846643000000	20.173327000000
C	4.022761000000	5.022294000000	18.157224000000
N	4.970893000000	5.440257000000	19.003396000000
H	1.520623000000	8.417385000000	18.951676000000
H	0.565721000000	7.611208000000	21.618925000000
H	0.986978000000	5.352359000000	20.782681000000
H	2.175450000000	5.812859000000	21.992032000000
H	4.898987000000	6.237455000000	21.000381000000
H	4.189337000000	4.664342000000	17.157828000000
C	10.802023000000	0.345506000000	19.168226000000
C	10.126072000000	1.676473000000	19.530193000000
N	10.196263000000	2.665303000000	18.448240000000
C	11.242241000000	3.476070000000	18.213885000000
N	12.339312000000	3.482438000000	19.003527000000
N	11.222917000000	4.308210000000	17.159387000000
H	11.867057000000	0.491845000000	18.958095000000
H	10.337372000000	-0.096238000000	18.279858000000
H	9.062464000000	1.528676000000	19.732325000000
H	10.563113000000	2.092739000000	20.445552000000
H	9.350130000000	2.859263000000	17.907090000000

H	12.305974000000	3.113853000000	19.952575000000
H	12.999837000000	4.237694000000	18.819290000000
H	10.407646000000	4.370946000000	16.566024000000
H	11.861806000000	5.101615000000	17.169998000000
Fe	6.926344000000	5.700191000000	18.582179000000
O	7.525440000000	3.985626000000	19.751103000000
O	8.517512000000	6.008052000000	18.239933000000
C	7.635158000000	4.684540000000	20.844848000000
O	7.270947000000	5.924056000000	20.805408000000
C	8.201274000000	4.055702000000	22.092714000000
C	7.974859000000	4.877114000000	23.367231000000
H	7.771063000000	3.049881000000	22.179860000000
H	9.279619000000	3.912413000000	21.925791000000
H	6.914177000000	5.109505000000	23.510264000000
H	8.526587000000	5.820085000000	23.326725000000
N	11.841042000000	8.337814000000	16.969493000000
C	12.442504000000	8.532142000000	18.330982000000
C	13.338677000000	7.334798000000	18.725185000000
O	13.080508000000	6.190583000000	18.194646000000
C	11.305320000000	8.732696000000	19.371646000000
C	11.815925000000	9.325341000000	20.708545000000
C	11.717713000000	10.864568000000	20.788799000000
N	10.375029000000	11.353551000000	21.124361000000
C	9.963044000000	11.649657000000	22.380120000000
N	10.746206000000	11.568743000000	23.460273000000
N	8.691002000000	12.074824000000	22.565796000000
O	14.226846000000	7.590983000000	19.609447000000
H	13.068575000000	9.428463000000	18.285491000000
H	10.542836000000	9.393978000000	18.934584000000
H	10.817758000000	7.762780000000	19.533941000000
H	12.861047000000	9.030484000000	20.845473000000
H	11.259973000000	8.896509000000	21.549757000000
H	12.001292000000	11.317634000000	19.830968000000
H	12.427180000000	11.238407000000	21.531965000000
H	9.690691000000	11.543757000000	20.385099000000
H	11.678967000000	11.159462000000	23.463319000000
H	10.306695000000	11.928863000000	24.338571000000
H	8.062417000000	12.102521000000	21.773978000000
H	8.408514000000	12.250436000000	23.536592000000
H	12.527157000000	8.230074000000	16.206598000000
H	11.225513000000	9.147916000000	16.674774000000
H	11.233151000000	7.487289000000	16.884112000000
O	12.289294000000	8.181155000000	14.209861000000
H	11.286286000000	8.351400000000	14.204370000000
H	12.492701000000	7.324030000000	13.796122000000
O	9.956180000000	6.548797000000	16.029898000000
H	9.312520000000	6.408579000000	16.769761000000
H	9.660549000000	7.229693000000	15.370926000000
H	10.712507000000	-0.366821000000	19.997457000000
H	2.730118000000	-1.667038000000	19.718670000000
H	-1.653610000000	-0.159857000000	18.047290000000
H	-0.181713000000	2.543885000000	14.295542000000
H	5.317716000000	-2.390079000000	15.498711000000
H	7.058820000000	-2.670077000000	15.284571000000
H	-4.739803000000	4.772958000000	18.767938000000
H	-4.602471000000	4.935423000000	20.525597000000
H	2.200372000000	8.141564000000	21.166254000000
H	-3.652488000000	6.778310000000	14.889476000000
H	6.343166000000	6.936447000000	13.156414000000
H	3.390160000000	10.995108000000	18.575288000000
H	8.777278000000	16.457535000000	18.811404000000
H	9.205391000000	11.113224000000	12.628899000000
H	14.516881000000	4.725302000000	20.534551000000
H	10.414301000000	4.724072000000	25.363067000000
H	14.659347000000	12.875238000000	13.566380000000
H	10.536218000000	14.311833000000	16.400347000000
H	10.104583000000	19.965728000000	22.816493000000
H	8.321108000000	4.315389000000	24.242435000000
C	1.539579000000	4.716941000000	18.121168000000
H	1.648268000000	4.798976000000	17.039430000000
H	1.313029000000	3.680296000000	18.391150000000
H	0.721264000000	5.351936000000	18.453081000000

$^{5}TSH_{Me}$ – VioC MeHis Transition state for HAT

C	11.399845000000	4.691605000000	25.900143000000
C	11.550559000000	5.929575000000	25.029753000000
O	11.363063000000	5.915964000000	23.787192000000
C	12.732018000000	4.013577000000	26.320033000000
O	13.398122000000	3.436836000000	25.171147000000
C	12.476840000000	2.882527000000	27.317644000000
H	10.836024000000	4.951825000000	26.806834000000
H	13.386934000000	4.767226000000	26.784809000000
H	14.111329000000	4.042506000000	24.825061000000
H	13.425628000000	2.413691000000	27.594437000000
H	11.983797000000	3.249247000000	28.225632000000
H	11.841394000000	2.117384000000	26.857510000000
N	11.917751000000	7.079099000000	25.686158000000
C	11.940226000000	8.399526000000	25.032024000000
C	13.027756000000	8.466479000000	23.948928000000
O	12.879141000000	9.158708000000	22.905944000000
C	12.122057000000	9.518009000000	26.089737000000
C	11.072961000000	9.528784000000	27.233747000000
C	11.413330000000	10.651890000000	28.236466000000
C	9.637616000000	9.696788000000	26.697400000000
H	11.951064000000	7.072021000000	26.696073000000
H	10.996041000000	8.547152000000	24.498435000000
H	13.130731000000	9.435819000000	26.520911000000
H	12.065346000000	10.481880000000	25.572052000000
H	11.133116000000	8.568443000000	27.781551000000
H	9.331184000000	8.854156000000	26.063128000000
H	9.570703000000	10.623814000000	26.115104000000
H	8.926128000000	9.757718000000	27.530084000000
H	12.444772000000	10.557952000000	28.603257000000
H	11.290119000000	11.639733000000	27.772995000000
H	10.743641000000	10.599968000000	29.104418000000
N	14.157941000000	7.758509000000	24.187241000000
C	15.093815000000	7.386312000000	23.116790000000
C	15.090959000000	5.856395000000	23.119719000000
O	15.139732000000	5.230613000000	24.232773000000
C	16.511906000000	8.008754000000	23.274661000000
C	16.446550000000	9.529469000000	23.037269000000
C	17.169613000000	7.684387000000	24.629379000000
H	14.183560000000	7.155972000000	25.001399000000
H	14.659757000000	7.743835000000	22.177902000000
H	17.122881000000	7.560513000000	22.475494000000
H	17.213195000000	6.607343000000	24.815845000000
H	18.189588000000	8.085785000000	24.656798000000
H	16.608968000000	8.149549000000	25.449573000000
H	17.448057000000	9.971164000000	23.099915000000
H	15.814779000000	10.012322000000	23.791236000000
H	16.027546000000	9.763386000000	22.052870000000
N	14.982408000000	5.252795000000	21.929898000000
C	14.756845000000	3.813260000000	21.799509000000
C	13.252879000000	3.510169000000	21.903617000000
O	12.968703000000	2.156883000000	21.397930000000
H	14.773102000000	5.820604000000	21.085922000000
H	15.319660000000	3.284156000000	22.574042000000
H	12.888335000000	3.618507000000	22.927064000000
H	12.696524000000	4.186295000000	21.253766000000
H	13.096958000000	1.490952000000	22.099373000000
C	3.741591000000	11.341967000000	16.859789000000
C	3.056635000000	10.107475000000	16.294794000000
O	2.361435000000	9.331228000000	17.011263000000
C	5.293963000000	11.271297000000	16.736440000000
C	5.909357000000	10.115388000000	17.467021000000
N	6.503276000000	10.284371000000	18.713523000000
C	6.017349000000	8.767285000000	17.179808000000
C	6.951864000000	9.082644000000	19.146763000000
N	6.672543000000	8.139015000000	18.235391000000
H	3.381986000000	12.235824000000	16.332980000000
H	5.578454000000	11.231229000000	15.678121000000
H	5.716149000000	12.200019000000	17.136324000000
H	5.685283000000	8.217200000000	16.317786000000

H	7.456795000000	8.911696000000	20.079994000000
H	6.735834000000	11.220548000000	19.139581000000
N	3.285200000000	9.865217000000	14.984324000000
C	2.979195000000	8.611741000000	14.289843000000
C	4.350437000000	8.120553000000	13.790784000000
O	5.173263000000	8.952483000000	13.317557000000
C	1.936162000000	8.838775000000	13.160789000000
O	0.709192000000	9.349577000000	13.721065000000
C	1.682881000000	7.573368000000	12.331566000000
H	3.910658000000	10.470659000000	14.464944000000
H	2.556223000000	7.920077000000	15.023067000000
H	2.314779000000	9.636666000000	12.512716000000
H	0.179588000000	8.626125000000	14.147547000000
H	0.907411000000	7.781475000000	11.588623000000
H	2.589586000000	7.252486000000	11.804232000000
H	1.329486000000	6.758695000000	12.974832000000
N	4.647951000000	6.813468000000	13.975106000000
C	6.031945000000	6.322604000000	13.843902000000
C	6.097024000000	4.799106000000	14.028447000000
C	7.469268000000	4.312308000000	14.530523000000
C	7.645383000000	4.307068000000	16.046860000000
O	8.334352000000	3.390632000000	16.568177000000
O	7.066955000000	5.284740000000	16.725210000000
H	3.945464000000	6.175712000000	14.347525000000
H	6.649442000000	6.811688000000	14.606048000000
H	5.859854000000	4.302510000000	13.078271000000
H	5.328588000000	4.484627000000	14.743372000000
H	7.670037000000	3.292368000000	14.192415000000
H	8.277399000000	4.942645000000	14.132338000000
C	5.672019000000	-1.750749000000	15.872171000000
C	6.349593000000	-0.409148000000	16.211504000000
C	7.738117000000	-0.284598000000	15.555753000000
C	5.448945000000	0.777898000000	15.817311000000
H	5.518674000000	-1.842089000000	14.787673000000
H	6.489378000000	-0.369271000000	17.303828000000
H	8.402471000000	-1.098322000000	15.873481000000
H	7.652568000000	-0.335232000000	14.461047000000
H	8.211449000000	0.669936000000	15.814371000000
H	5.907761000000	1.726833000000	16.117013000000
H	4.464649000000	0.692703000000	16.294612000000
H	5.302623000000	0.797970000000	14.727211000000
C	-3.943841000000	4.990312000000	19.441037000000
C	-3.607487000000	3.498859000000	19.610206000000
C	-2.623172000000	3.201270000000	20.768305000000
C	-2.576677000000	1.688227000000	21.061958000000
C	-1.209746000000	3.741725000000	20.469590000000
H	-3.048004000000	5.596008000000	19.263489000000
H	-4.540248000000	2.942973000000	19.790536000000
H	-3.179804000000	3.099408000000	18.678827000000
H	-2.998977000000	3.707117000000	21.672803000000
H	-1.872819000000	1.459188000000	21.872893000000
H	-3.562316000000	1.305346000000	21.353740000000
H	-2.264553000000	1.135849000000	20.163562000000
H	-1.210779000000	4.825866000000	20.315399000000
H	-0.523116000000	3.519101000000	21.297380000000
H	-0.815225000000	3.272328000000	19.556873000000
C	9.042432000000	11.443890000000	13.555160000000
C	9.655381000000	12.736845000000	14.091659000000
O	9.571097000000	13.793009000000	13.419234000000
C	8.624439000000	10.359648000000	14.581452000000
C	9.801644000000	9.561432000000	15.114818000000
O	10.324785000000	9.906330000000	16.243608000000
O	10.259294000000	8.586207000000	14.386065000000
H	8.167001000000	11.752034000000	12.975962000000
H	8.098033000000	10.816551000000	15.424154000000
H	7.937792000000	9.668684000000	14.082015000000
N	10.321344000000	12.675114000000	15.288618000000
C	10.961134000000	13.851296000000	15.868150000000
C	12.487348000000	13.775814000000	15.953526000000
O	13.120385000000	14.580317000000	16.684947000000
H	10.320029000000	11.811829000000	15.832239000000
H	10.691776000000	14.708464000000	15.242218000000
N	13.083174000000	12.826073000000	15.194524000000
C	14.520469000000	12.584889000000	15.159879000000
C	14.765721000000	11.078627000000	15.114446000000
O	14.021326000000	10.556835000000	13.988248000000
H	12.528489000000	12.212948000000	14.611227000000
H	14.970184000000	13.043299000000	16.044955000000
H	14.429302000000	10.622551000000	16.057527000000
H	15.842073000000	10.879175000000	15.003386000000
H	13.667277000000	9.645035000000	14.174088000000
C	6.232808000000	15.976548000000	20.684587000000
C	6.415109000000	16.280574000000	22.170120000000
O	6.703876000000	17.444069000000	22.571829000000
C	7.221170000000	14.974613000000	20.039421000000
C	6.947365000000	13.491740000000	20.278997000000
O	7.405236000000	12.666060000000	19.387223000000
O	6.355328000000	13.099494000000	21.350903000000
H	5.205547000000	15.622915000000	20.524470000000
H	7.236430000000	15.129645000000	18.956219000000
H	8.246265000000	15.183901000000	20.378077000000
N	6.218060000000	15.246159000000	23.031602000000
C	6.139944000000	15.448381000000	24.475030000000
C	7.446344000000	15.831752000000	25.182893000000
O	7.408306000000	16.349162000000	26.320649000000
H	6.079059000000	14.319579000000	22.606704000000
H	5.434977000000	16.252751000000	24.707919000000
H	5.762009000000	14.525681000000	24.926867000000
N	8.592496000000	15.568795000000	24.495711000000
C	9.929248000000	15.936421000000	24.963419000000
C	10.400691000000	17.291940000000	24.393056000000
O	11.593122000000	17.661616000000	24.564417000000
C	10.948503000000	14.830968000000	24.621101000000
C	10.720633000000	13.555066000000	25.449525000000
O	10.938109000000	13.605055000000	26.689892000000
O	10.305087000000	12.489068000000	24.795503000000
H	8.513256000000	15.114308000000	23.595883000000
H	9.876458000000	16.038019000000	26.053286000000
H	10.907521000000	14.602197000000	23.549055000000
H	11.940657000000	15.230617000000	24.848742000000
N	9.485703000000	18.031662000000	23.713764000000
C	9.811833000000	19.326745000000	23.116884000000
C	9.478286000000	19.386891000000	21.615911000000
C	10.257218000000	18.449632000000	20.701414000000
C	11.466292000000	17.838301000000	21.076421000000
C	9.762128000000	18.202771000000	19.406287000000
C	12.155433000000	17.011982000000	20.177755000000
C	10.455164000000	17.383357000000	18.508995000000
C	11.661813000000	16.779447000000	18.888883000000
H	8.536339000000	17.693604000000	23.583102000000
H	10.871605000000	19.509564000000	23.309732000000
H	8.405055000000	19.187216000000	21.495452000000
H	9.637808000000	20.426641000000	21.286946000000
H	11.868203000000	17.982368000000	22.074210000000
H	8.825334000000	18.665243000000	19.101605000000
H	13.084007000000	16.543734000000	20.491206000000
H	10.055336000000	17.214082000000	17.512561000000
H	12.199946000000	16.137602000000	18.196380000000
C	2.595857000000	-0.252091000000	19.890226000000
C	1.731676000000	-0.240799000000	18.644606000000
O	2.193349000000	-0.504328000000	17.496845000000
C	3.932266000000	0.513658000000	19.722341000000
C	4.831938000000	0.284085000000	20.960642000000
C	3.683435000000	2.009849000000	19.456770000000
C	6.276208000000	0.783626000000	20.786901000000
H	2.039741000000	0.153442000000	20.746675000000
H	4.439386000000	0.083978000000	18.847178000000
H	4.853509000000	-0.792828000000	21.186109000000
H	4.372759000000	0.773471000000	21.833579000000
H	6.873582000000	0.553769000000	21.677152000000
H	6.754679000000	0.294567000000	19.928655000000
H	6.329979000000	1.865141000000	20.622909000000

H	3.068980000000	2.156006000000	18.560610000000
H	4.621258000000	2.551397000000	19.302587000000
H	3.165248000000	2.475614000000	20.306279000000
N	0.408854000000	0.047339000000	18.816875000000
C	-0.562900000000	-0.028938000000	17.717294000000
C	-0.545818000000	1.205961000000	16.808332000000
O	-1.476759000000	2.049960000000	16.822310000000
H	0.074102000000	0.340855000000	19.723396000000
H	-0.346660000000	-0.928205000000	17.131029000000
N	0.546605000000	1.311752000000	16.007666000000
C	0.739956000000	2.485555000000	15.155375000000
C	2.016259000000	2.341496000000	14.309027000000
C	2.498397000000	3.693536000000	13.813611000000
O	2.728397000000	4.635341000000	14.621819000000
N	2.689830000000	3.837561000000	12.479486000000
H	1.298427000000	0.635926000000	16.146555000000
H	0.811263000000	3.386005000000	15.773404000000
H	1.858323000000	1.641667000000	13.481277000000
H	2.823523000000	1.933169000000	14.931678000000
H	2.500633000000	3.094759000000	11.825967000000
H	3.015646000000	4.725030000000	12.122147000000
C	-2.908582000000	6.222046000000	15.371903000000
C	-1.720584000000	7.114373000000	15.703420000000
O	-0.813933000000	7.328694000000	14.846813000000
C	-2.494430000000	4.897463000000	14.709116000000
H	-3.506832000000	6.031448000000	16.270470000000
H	-2.014337000000	4.222162000000	15.425287000000
H	-3.374337000000	4.382270000000	14.308842000000
H	-1.802811000000	5.097399000000	13.884598000000
N	-1.721347000000	7.671375000000	16.939306000000
C	-0.664104000000	8.539173000000	17.461248000000
C	-0.248475000000	7.989021000000	18.832325000000
O	-1.059907000000	7.325723000000	19.541216000000
C	-1.104041000000	10.038525000000	17.568519000000
C	-1.358916000000	10.637933000000	16.173501000000
C	-2.323237000000	10.233520000000	18.489868000000
H	-2.390025000000	7.352197000000	17.629893000000
H	0.183174000000	8.491678000000	16.773414000000
H	-0.243392000000	10.558918000000	18.012242000000
H	-0.491447000000	10.530086000000	15.514541000000
H	-1.595786000000	11.705342000000	16.260873000000
H	-2.214299000000	10.149149000000	15.690586000000
H	-2.165674000000	9.806606000000	19.485679000000
H	-2.538601000000	11.301873000000	18.606222000000
H	-3.216054000000	9.763929000000	18.057637000000
N	1.012112000000	8.287099000000	19.226357000000
C	1.516702000000	7.923772000000	20.551688000000
C	2.005091000000	6.454362000000	20.670979000000
C	3.179749000000	6.103900000000	19.809430000000
N	3.071605000000	5.551817000000	18.524040000000
C	4.533105000000	6.249631000000	20.030558000000
C	4.332778000000	5.388630000000	18.026670000000
N	5.239237000000	5.803350000000	18.915287000000
H	1.631285000000	8.765709000000	18.565876000000
H	0.713076000000	8.075423000000	21.279435000000
H	1.156457000000	5.794008000000	20.463004000000
H	2.280807000000	6.288384000000	21.720062000000
H	5.051187000000	6.650386000000	20.884241000000
H	4.548870000000	5.000046000000	17.048541000000
C	10.453669000000	0.152208000000	19.111174000000
C	10.130230000000	1.608532000000	19.477684000000
N	10.291373000000	2.532281000000	18.349359000000
C	11.410014000000	3.216633000000	18.054781000000
N	12.553386000000	3.077728000000	18.760491000000
N	11.416838000000	4.078680000000	17.022908000000
H	11.498588000000	0.049887000000	18.797489000000
H	9.817622000000	-0.192669000000	18.288751000000
H	9.090077000000	1.704636000000	19.801861000000
H	10.754721000000	1.932406000000	20.316958000000
H	9.452566000000	2.795783000000	17.823387000000
H	12.633360000000	2.495096000000	19.591753000000
H	13.287795000000	3.755225000000	18.564369000000
H	10.582693000000	4.308649000000	16.498566000000
H	12.185899000000	4.746700000000	16.977483000000
Fe	7.330398000000	6.157337000000	18.492928000000
O	7.833854000000	4.497932000000	19.862595000000
O	9.038411000000	6.637351000000	18.194095000000
C	7.857906000000	5.301414000000	20.881120000000
O	7.515028000000	6.541184000000	20.678703000000
C	8.301517000000	4.830922000000	22.240432000000
C	7.517086000000	5.476702000000	23.393682000000
H	8.224918000000	3.738944000000	22.262058000000
H	9.367226000000	5.080624000000	22.356846000000
H	6.454061000000	5.210135000000	23.353927000000
H	7.595770000000	6.566166000000	23.345785000000
N	11.934004000000	7.896575000000	17.157825000000
C	12.295619000000	7.773946000000	18.612040000000
C	13.356553000000	6.662737000000	18.816884000000
O	13.458545000000	5.746081000000	17.923117000000
C	11.050621000000	7.492947000000	19.495861000000
C	10.560793000000	8.580025000000	20.453031000000
C	10.438977000000	10.011884000000	19.874819000000
N	9.529311000000	10.882842000000	20.642673000000
C	9.770865000000	11.357479000000	21.896310000000
N	10.891510000000	11.095634000000	22.575420000000
N	8.855788000000	12.141984000000	22.499875000000
O	14.005951000000	6.752652000000	19.911579000000
H	12.751034000000	8.721943000000	18.911529000000
H	10.069916000000	7.170010000000	18.780920000000
H	11.173760000000	6.554822000000	20.046583000000
H	11.248549000000	8.577384000000	21.311992000000
H	9.586177000000	8.262585000000	20.842610000000
H	10.031287000000	9.979644000000	18.859451000000
H	11.431749000000	10.480483000000	19.807551000000
H	8.803318000000	11.379804000000	20.122925000000
H	11.613251000000	10.431732000000	22.317535000000
H	10.949488000000	11.545314000000	23.507342000000
H	7.921848000000	12.289375000000	22.122449000000
H	9.110971000000	12.457463000000	23.450344000000
H	12.749439000000	7.952364000000	16.524951000000
H	11.341372000000	8.748233000000	16.934305000000
H	11.391404000000	7.078503000000	16.799534000000
O	12.854679000000	8.190910000000	14.558716000000
H	11.854984000000	8.342757000000	14.425113000000
H	13.156164000000	7.406125000000	14.067915000000
O	10.105058000000	6.235243000000	15.794597000000
H	9.526365000000	6.389467000000	16.597174000000
H	10.003199000000	6.965315000000	15.131778000000
H	10.289936000000	-0.504654000000	19.974291000000
H	2.815113000000	-1.304434000000	20.120748000000
H	-1.562761000000	-0.106138000000	18.143579000000
H	-0.138554000000	2.615392000000	14.513920000000
H	4.692793000000	-1.829187000000	16.359843000000
H	6.288323000000	-2.599955000000	16.193182000000
H	-4.633270000000	5.139843000000	18.599996000000
H	-4.431208000000	5.387586000000	20.341106000000
H	2.338643000000	8.604407000000	20.791614000000
H	-3.543714000000	6.793166000000	14.679844000000
H	6.431956000000	6.623148000000	12.869583000000
H	3.451246000000	11.429359000000	17.909221000000
H	6.337417000000	16.939586000000	20.178862000000
H	9.748625000000	11.006535000000	12.835300000000
H	15.124069000000	3.494341000000	20.819522000000
H	10.817502000000	3.961667000000	25.331878000000
H	14.964083000000	13.051488000000	14.270060000000
H	10.589835000000	14.038703000000	16.880782000000
H	9.244470000000	20.115710000000	23.629603000000
H	7.916378000000	5.144150000000	24.357452000000
C	1.844300000000	5.176733000000	17.819677000000
H	2.109636000000	4.899023000000	16.798788000000
H	1.354187000000	4.335154000000	18.319297000000
H	1.156537000000	6.020068000000	17.786089000000

$^{5}I_{me}$ – VioC MeHis Intermediate state

C	11.099784000000	4.647261000000	25.503961000000
C	11.262877000000	6.041496000000	24.920300000000
O	11.155528000000	6.266790000000	23.688799000000
C	12.400171000000	3.804108000000	25.608641000000
O	13.012376000000	3.599234000000	24.308421000000
C	12.109334000000	2.428513000000	26.208404000000
H	10.648232000000	4.707177000000	26.503236000000
H	13.119727000000	4.339248000000	26.245095000000
H	13.725623000000	4.291333000000	24.155424000000
H	13.036535000000	1.852736000000	26.279738000000
H	11.666702000000	2.512016000000	27.207846000000
H	11.414800000000	1.877976000000	25.563686000000
N	11.535394000000	7.065093000000	25.794557000000
C	11.507455000000	8.470930000000	25.346063000000
C	12.717830000000	8.812733000000	24.459133000000
O	12.654506000000	9.738990000000	23.608464000000
C	11.375532000000	9.442786000000	26.543486000000
C	10.195781000000	9.152980000000	27.511323000000
C	10.140394000000	10.244814000000	28.601991000000
C	8.839955000000	9.051465000000	26.784213000000
H	11.494103000000	6.892083000000	26.789214000000
H	10.641627000000	8.594874000000	24.687433000000
H	12.317608000000	9.449051000000	27.111676000000
H	11.241620000000	10.447240000000	26.126913000000
H	10.394576000000	8.190563000000	28.020998000000
H	8.816431000000	8.217705000000	26.069921000000
H	8.628245000000	9.986455000000	26.248826000000
H	8.036151000000	8.883286000000	27.511852000000
H	11.118407000000	10.383396000000	29.082014000000
H	9.813951000000	11.203195000000	28.179940000000
H	9.419953000000	9.966749000000	29.381085000000
N	13.841412000000	8.074941000000	24.643911000000
C	14.886080000000	7.954549000000	23.612071000000
C	14.822496000000	6.485889000000	23.184672000000
O	14.702974000000	5.592211000000	24.098903000000
C	16.291932000000	8.419879000000	24.087817000000
C	16.294653000000	9.945998000000	24.297045000000
C	16.780574000000	7.684393000000	25.350196000000
H	13.786118000000	7.283069000000	25.273783000000
H	14.579742000000	8.588371000000	22.775264000000
H	16.978606000000	8.182342000000	23.260478000000
H	16.767159000000	6.598335000000	25.221152000000
H	17.803346000000	7.995694000000	25.593648000000
H	16.145757000000	7.932867000000	26.209836000000
H	17.292521000000	10.292302000000	24.590814000000
H	15.589828000000	10.227258000000	25.087665000000
H	16.001450000000	10.476275000000	23.384482000000
N	14.816915000000	6.204860000000	21.881290000000
C	14.481053000000	4.856107000000	21.410776000000
C	12.947495000000	4.647717000000	21.429524000000
O	12.608581000000	3.234635000000	21.615739000000
H	14.800319000000	6.949366000000	21.147337000000
H	14.958849000000	4.115355000000	22.058047000000
H	12.488172000000	5.233354000000	22.227919000000
H	12.517387000000	4.961390000000	20.477567000000
H	12.606001000000	3.061831000000	22.594383000000
C	3.736458000000	11.361875000000	16.496338000000
C	3.116085000000	10.064947000000	16.002871000000
O	2.411912000000	9.337137000000	16.761317000000
C	5.287256000000	11.400817000000	16.389134000000
C	5.980760000000	10.265122000000	17.085928000000
N	6.810074000000	10.462381000000	18.185809000000
C	5.994055000000	8.901591000000	16.852665000000
C	7.300454000000	9.264058000000	18.583475000000
N	6.822498000000	8.290881000000	17.790556000000
H	3.322061000000	12.202482000000	15.924135000000
H	5.588805000000	11.444368000000	15.335112000000
H	5.630039000000	12.344379000000	16.826656000000
H	5.482070000000	8.327028000000	16.102194000000
H	7.966212000000	9.114298000000	19.414872000000
H	7.126702000000	11.384871000000	18.587923000000
N	3.384698000000	9.710411000000	14.725938000000
C	2.996637000000	8.432236000000	14.119964000000
C	4.301565000000	7.844525000000	13.560319000000
O	5.118191000000	8.592582000000	12.956929000000
C	1.887806000000	8.639615000000	13.048500000000
O	0.713134000000	9.207675000000	13.664808000000
C	1.546862000000	7.348334000000	12.293848000000
H	4.007489000000	10.274094000000	14.157891000000
H	2.592224000000	7.800669000000	14.915419000000
H	2.244380000000	9.398113000000	12.343524000000
H	0.181605000000	8.514420000000	14.136282000000
H	0.725746000000	7.546433000000	11.598736000000
H	2.406099000000	6.982488000000	11.718542000000
H	1.219073000000	6.571452000000	12.994889000000
N	4.547304000000	6.536401000000	13.815622000000
C	5.884586000000	5.955814000000	13.597011000000
C	5.902834000000	4.450821000000	13.909751000000
C	7.296758000000	3.942265000000	14.308314000000
C	7.713363000000	4.208382000000	15.759855000000
O	8.715225000000	3.599135000000	16.212890000000
O	6.977625000000	5.069806000000	16.444318000000
H	3.855422000000	5.973445000000	14.308827000000
H	6.594987000000	6.473753000000	14.250001000000
H	5.542377000000	3.887045000000	13.038876000000
H	5.211654000000	4.242099000000	14.732435000000
H	7.373288000000	2.860345000000	14.154671000000
H	8.072405000000	4.388242000000	13.668117000000
C	6.151624000000	-2.026403000000	15.618056000000
C	6.512448000000	-0.592970000000	16.053959000000
C	7.785135000000	-0.095370000000	15.341829000000
C	5.329146000000	0.367493000000	15.818997000000
H	5.944972000000	-2.061425000000	14.539409000000
H	6.717589000000	-0.610954000000	17.136448000000
H	8.629533000000	-0.772184000000	15.523032000000
H	7.625690000000	-0.051089000000	14.254997000000
H	8.076153000000	0.905204000000	15.683318000000
H	5.575627000000	1.381042000000	16.159335000000
H	4.432946000000	0.030555000000	16.355063000000
H	5.089508000000	0.419482000000	14.746812000000
C	-3.705312000000	5.271250000000	20.029966000000
C	-3.385951000000	3.786071000000	20.271987000000
C	-2.302929000000	3.540326000000	21.351491000000
C	-2.257318000000	2.049553000000	21.742687000000
C	-0.913775000000	4.023282000000	20.886061000000
H	-2.817336000000	5.838119000000	19.729357000000
H	-4.308992000000	3.269454000000	20.576480000000
H	-3.056551000000	3.313875000000	19.334666000000
H	-2.581686000000	4.114284000000	22.250354000000
H	-1.491250000000	1.859387000000	22.505814000000
H	-3.220851000000	1.708731000000	22.140995000000
H	-2.030594000000	1.430782000000	20.861871000000
H	-0.905784000000	5.095599000000	20.664520000000
H	-0.155458000000	3.831726000000	21.657412000000
H	-0.620318000000	3.489691000000	19.970809000000
C	8.275310000000	10.306773000000	13.157119000000
C	8.137888000000	11.596239000000	13.967175000000
O	7.373740000000	12.507797000000	13.555197000000
C	8.472476000000	8.961960000000	13.907063000000
C	9.847509000000	8.751759000000	14.514745000000
O	10.061350000000	9.211495000000	15.711854000000
O	10.744802000000	8.125006000000	13.827160000000
H	7.354381000000	10.226557000000	12.574205000000
H	7.719966000000	8.874399000000	14.695294000000
H	8.303427000000	8.163546000000	13.180402000000
N	8.910890000000	11.735086000000	15.087500000000
C	8.960909000000	12.983658000000	15.846470000000
C	10.349056000000	13.619284000000	15.920835000000
O	10.551007000000	14.593782000000	16.693839000000
H	9.360243000000	10.907711000000	15.487756000000
H	8.275857000000	13.689000000000	15.366155000000

N	11.305669000000	13.069340000000	15.135336000000
C	12.707062000000	13.468543000000	15.120707000000
C	13.575989000000	12.212748000000	15.076612000000
O	13.139846000000	11.435461000000	13.935885000000
H	11.072086000000	12.297702000000	14.523352000000
H	12.910042000000	14.067630000000	16.012415000000
H	13.452255000000	11.648238000000	16.013395000000
H	14.635652000000	12.494508000000	14.984428000000
H	13.270433000000	10.459559000000	14.077010000000
C	7.495235000000	16.287152000000	19.889852000000
C	7.283473000000	16.630569000000	21.362188000000
O	7.788250000000	17.670279000000	21.875624000000
C	8.285872000000	14.995806000000	19.572216000000
C	7.520089000000	13.684965000000	19.687712000000
O	7.998920000000	12.674543000000	19.009949000000
O	6.513080000000	13.560729000000	20.469813000000
H	6.514818000000	16.240694000000	19.397503000000
H	8.702041000000	15.054625000000	18.560735000000
H	9.164860000000	14.923332000000	20.229672000000
N	6.496990000000	15.780674000000	22.078032000000
C	6.005997000000	16.118246000000	23.410900000000
C	7.051671000000	16.185567000000	24.531059000000
O	6.803498000000	16.828342000000	25.574687000000
H	6.219937000000	14.910874000000	21.606085000000
H	5.525499000000	17.101824000000	23.402297000000
H	5.250970000000	15.375948000000	23.689805000000
N	8.211725000000	15.509009000000	24.304156000000
C	9.348247000000	15.511408000000	25.226215000000
C	10.379049000000	16.609845000000	24.890357000000
O	11.484661000000	16.636456000000	25.496003000000
C	10.037703000000	14.135061000000	25.235952000000
C	9.169755000000	13.040260000000	25.885376000000
O	8.762719000000	13.217162000000	27.063807000000
O	8.942943000000	11.963165000000	25.157038000000
H	8.301116000000	14.991378000000	23.439703000000
H	8.956058000000	15.710554000000	26.229543000000
H	10.321386000000	13.849527000000	24.215910000000
H	10.958564000000	14.245029000000	25.818163000000
N	10.043972000000	17.501495000000	23.921294000000
C	10.964330000000	18.542716000000	23.469238000000
C	11.233182000000	18.468342000000	21.953901000000
C	11.843838000000	17.171410000000	21.440301000000
C	12.663082000000	16.348422000000	22.233777000000
C	11.595752000000	16.779220000000	20.111460000000
C	13.220200000000	15.177041000000	21.704124000000
C	12.145363000000	15.607690000000	19.577121000000
C	12.967227000000	14.804457000000	20.378754000000
H	9.145246000000	17.445721000000	23.449136000000
H	11.880013000000	18.432200000000	24.055034000000
H	10.284971000000	18.640623000000	21.428751000000
H	11.891657000000	19.313095000000	21.693258000000
H	12.840190000000	16.592279000000	23.276257000000
H	10.953271000000	17.396769000000	19.488479000000
H	13.842336000000	14.550065000000	22.336557000000
H	11.905173000000	15.318258000000	18.557826000000
H	13.397738000000	13.891280000000	19.976936000000
C	2.785275000000	-0.070488000000	20.196150000000
C	1.805070000000	-0.106481000000	19.039285000000
O	2.144922000000	-0.465256000000	17.875305000000
C	4.145357000000	0.576422000000	19.836127000000
C	5.141440000000	0.389924000000	21.005429000000
C	3.969454000000	2.056911000000	19.451581000000
C	6.583285000000	0.813719000000	20.678764000000
H	2.342121000000	0.447684000000	21.057850000000
H	4.535553000000	0.035466000000	18.962717000000
H	5.139741000000	-0.669677000000	21.302428000000
H	4.780235000000	0.956176000000	21.877900000000
H	7.250467000000	0.596444000000	21.520866000000
H	6.961948000000	0.269239000000	19.804218000000
H	6.661934000000	1.884793000000	20.464199000000
H	3.277592000000	2.164375000000	18.608112000000
H	4.919047000000	2.511624000000	19.154615000000
H	3.573572000000	2.636369000000	20.296954000000
N	0.515259000000	0.243651000000	19.315901000000
C	-0.562314000000	0.125393000000	18.323770000000
C	-0.594760000000	1.290382000000	17.327106000000
O	-1.498237000000	2.163183000000	17.356542000000
H	0.279508000000	0.609054000000	20.227516000000
H	-0.432688000000	-0.820844000000	17.787796000000
N	0.427420000000	1.303178000000	16.432393000000
C	0.584999000000	2.409851000000	15.488411000000
C	1.860733000000	2.226998000000	14.647030000000
C	2.311956000000	3.549199000000	14.052742000000
O	2.597442000000	4.526815000000	14.797792000000
N	2.405780000000	3.629302000000	12.702778000000
H	1.165095000000	0.607850000000	16.550629000000
H	0.638552000000	3.355421000000	16.037337000000
H	1.712364000000	1.465406000000	13.873739000000
H	2.677055000000	1.881694000000	15.294862000000
H	2.172126000000	2.856316000000	12.100746000000
H	2.701849000000	4.499054000000	12.281276000000
C	-2.904611000000	6.186972000000	15.615159000000
C	-1.693119000000	7.083277000000	15.832935000000
O	-0.851220000000	7.265644000000	14.905758000000
C	-2.535982000000	4.821263000000	15.011361000000
H	-3.459130000000	6.055224000000	16.551573000000
H	-2.044183000000	4.178436000000	15.749055000000
H	-3.437157000000	4.301217000000	14.668645000000
H	-1.869591000000	4.964967000000	14.154857000000
N	-1.604198000000	7.678588000000	17.047273000000
C	-0.520644000000	8.572474000000	17.456077000000
C	-0.017041000000	8.102833000000	18.827040000000
O	-0.771934000000	7.460030000000	19.614479000000
C	-0.960332000000	10.075790000000	17.510564000000
C	-1.307598000000	10.600008000000	16.105370000000
C	-2.117363000000	10.318094000000	18.498503000000
H	-2.220865000000	7.384295000000	17.794899000000
H	0.283886000000	8.489478000000	16.722275000000
H	-0.075375000000	10.620360000000	17.868345000000
H	-0.479320000000	10.465907000000	15.402463000000
H	-1.550443000000	11.668647000000	16.152770000000
H	-2.185150000000	10.079945000000	15.701540000000
H	-1.891198000000	9.947727000000	19.503816000000
H	-2.331342000000	11.390657000000	18.570260000000
H	-3.033921000000	9.822523000000	18.153908000000
N	1.253549000000	8.451947000000	19.133296000000
C	1.837592000000	8.211141000000	20.453699000000
C	2.364973000000	6.767323000000	20.675613000000
C	3.448324000000	6.341519000000	19.732542000000
N	3.214070000000	5.611598000000	18.556326000000
C	4.808193000000	6.567827000000	19.756946000000
C	4.411829000000	5.424936000000	17.928089000000
N	5.395714000000	5.993071000000	18.631361000000
H	1.823125000000	8.892037000000	18.404099000000
H	1.075730000000	8.415458000000	21.213073000000
H	1.514608000000	6.079237000000	20.627581000000
H	2.750299000000	6.717756000000	21.701850000000
H	5.404106000000	7.101397000000	20.476825000000
H	4.533225000000	4.906528000000	16.994906000000
C	10.401006000000	0.518368000000	18.651881000000
C	10.170767000000	1.977337000000	19.069997000000
N	10.720742000000	2.946457000000	18.108500000000
C	12.008005000000	3.312525000000	18.046936000000
N	12.910150000000	2.824200000000	18.940697000000
N	12.449188000000	4.158342000000	17.098369000000
H	11.470566000000	0.294130000000	18.582137000000
H	9.943540000000	0.320670000000	17.676742000000
H	9.105017000000	2.206971000000	19.132604000000
H	10.591015000000	2.169283000000	20.063954000000
H	10.040712000000	3.380661000000	17.474929000000
H	12.666399000000	2.622580000000	19.917670000000
H	13.874748000000	3.072173000000	18.780532000000

H	11.807585000000	4.579038000000	16.408352000000
H	13.201098000000	4.797121000000	17.366479000000
Fe	7.444940000000	6.255773000000	17.978673000000
O	8.156410000000	4.731683000000	19.340486000000
O	9.079869000000	6.827682000000	17.204843000000
C	8.227325000000	5.570205000000	20.335016000000
O	7.886552000000	6.799056000000	20.089965000000
C	8.670870000000	5.136890000000	21.701433000000
C	7.776459000000	5.703305000000	22.819024000000
H	8.696822000000	4.043068000000	21.720811000000
H	9.696927000000	5.490826000000	21.868964000000
H	6.743261000000	5.347465000000	22.725300000000
H	7.768120000000	6.795979000000	22.782466000000
N	12.330385000000	8.202699000000	16.874376000000
C	12.726135000000	8.581363000000	18.273574000000
C	13.742185000000	7.526180000000	18.839607000000
O	13.778556000000	6.375706000000	18.284370000000
C	11.513308000000	8.682330000000	19.155092000000
C	11.582316000000	9.440466000000	20.444736000000
C	11.227988000000	10.967946000000	20.350832000000
N	9.849693000000	11.312770000000	20.732426000000
C	9.485594000000	11.463644000000	22.031896000000
N	10.300183000000	11.149235000000	23.040687000000
N	8.271832000000	11.968694000000	22.361276000000
O	14.402771000000	7.934622000000	19.858647000000
H	13.258096000000	9.539058000000	18.228748000000
H	9.185907000000	7.781123000000	16.992515000000
H	10.668216000000	8.036614000000	18.925616000000
H	12.601576000000	9.362835000000	20.843264000000
H	10.920592000000	8.982014000000	21.187325000000
H	11.366919000000	11.327204000000	19.327686000000
H	11.919014000000	11.533672000000	20.988384000000
H	9.233530000000	11.714619000000	20.018214000000
H	11.203376000000	10.689014000000	22.958958000000
H	9.944355000000	11.406720000000	23.993146000000
H	7.582311000000	12.275018000000	21.682529000000
H	8.087209000000	12.054306000000	23.364908000000
H	13.054115000000	8.392674000000	16.156674000000
H	11.473277000000	8.716068000000	16.537258000000
H	12.102848000000	7.191432000000	16.791929000000
O	13.230788000000	8.735581000000	14.312194000000
H	12.266094000000	8.480041000000	14.073436000000
H	13.869085000000	8.258084000000	13.753551000000
O	11.012281000000	5.949185000000	15.724112000000
H	10.192969000000	6.151337000000	16.292359000000
H	10.949036000000	6.409654000000	14.859825000000
H	9.955745000000	-0.162090000000	19.388142000000
H	2.956285000000	-1.111918000000	20.505153000000
H	-1.517731000000	0.115244000000	18.847826000000
H	-0.298265000000	2.472997000000	14.842513000000
H	5.259543000000	-2.388874000000	16.143317000000
H	6.974484000000	-2.722402000000	15.822544000000
H	-4.466790000000	5.381943000000	19.247056000000
H	-4.098034000000	5.742164000000	20.940734000000
H	2.657390000000	8.923546000000	20.583954000000
H	-3.567470000000	6.725170000000	14.923071000000
H	6.197355000000	6.149547000000	12.564703000000
H	3.429636000000	11.482512000000	17.537973000000
H	8.038859000000	17.136159000000	19.468352000000
H	9.100417000000	10.449176000000	12.444008000000
H	14.873364000000	4.758659000000	20.395972000000
H	10.405529000000	4.110822000000	24.849926000000
H	12.919582000000	14.086276000000	14.238035000000
H	8.632574000000	12.836098000000	16.882299000000
H	10.544193000000	19.531199000000	23.699660000000
H	8.157229000000	5.397466000000	23.798331000000
C	1.931801000000	5.084232000000	18.083673000000
H	2.026615000000	4.859753000000	17.020434000000
H	1.663146000000	4.175473000000	18.632010000000
H	1.145154000000	5.823773000000	18.222934000000

$^5TS_{me,reb}$ – VioC MeHis Transition state for radical rebound

C	11.514792000000	4.624485000000	25.492739000000
C	11.684571000000	5.976661000000	24.818189000000
O	11.518730000000	6.134115000000	23.581099000000
C	12.812526000000	3.789999000000	25.667922000000
O	13.413210000000	3.463388000000	24.385949000000
C	12.521085000000	2.475797000000	26.392292000000
H	11.056585000000	4.757603000000	26.482144000000
H	13.539069000000	4.377627000000	26.247656000000
H	14.139595000000	4.122020000000	24.170011000000
H	13.446387000000	1.904246000000	26.508066000000
H	12.087024000000	2.651880000000	27.383345000000
H	11.818862000000	1.872435000000	25.805839000000
N	12.032075000000	7.036467000000	25.614506000000
C	12.042126000000	8.417465000000	25.091998000000
C	13.155814000000	8.609243000000	24.049476000000
O	12.999176000000	9.355149000000	23.047248000000
C	12.155470000000	9.435692000000	26.252644000000
C	11.065179000000	9.310685000000	27.350302000000
C	11.302096000000	10.382939000000	28.434920000000
C	9.642005000000	9.433787000000	26.769753000000
H	12.042410000000	6.921241000000	26.618429000000
H	11.109338000000	8.588097000000	24.546054000000
H	13.150767000000	9.344974000000	26.712339000000
H	12.087280000000	10.440044000000	25.819977000000
H	11.163347000000	8.319766000000	27.834786000000
H	9.402952000000	8.611352000000	26.082046000000
H	9.540238000000	10.385422000000	26.232838000000
H	8.900932000000	9.412120000000	27.578143000000
H	12.326401000000	10.332282000000	28.829027000000
H	11.126010000000	11.386702000000	28.026289000000
H	10.611654000000	10.231648000000	29.274290000000
N	14.319268000000	7.949617000000	24.282205000000
C	15.309033000000	7.710878000000	23.219799000000
C	15.234148000000	6.204974000000	22.960585000000
O	15.151167000000	5.409185000000	23.962756000000
C	16.735053000000	8.223187000000	23.572459000000
C	16.750184000000	9.763128000000	23.596475000000
C	17.278644000000	7.639780000000	24.890694000000
H	14.332275000000	7.265684000000	25.029543000000
H	14.953218000000	8.245096000000	22.333752000000
H	17.383699000000	7.883454000000	22.750092000000
H	17.252216000000	6.546156000000	24.895005000000
H	18.313829000000	7.965364000000	25.046662000000
H	16.688388000000	7.999288000000	25.742741000000
H	17.760294000000	10.134449000000	23.805903000000
H	16.080366000000	10.143799000000	24.375849000000
H	16.422443000000	10.182184000000	22.638835000000
N	15.178635000000	5.794785000000	21.690635000000
C	14.792879000000	4.414741000000	21.377068000000
C	13.257702000000	4.276934000000	21.474254000000
O	12.868164000000	2.888399000000	21.721932000000
H	15.068952000000	6.479660000000	20.913236000000
H	15.273028000000	3.733459000000	22.084318000000
H	12.861855000000	4.908905000000	22.270160000000
H	12.798342000000	4.581256000000	20.534195000000
H	12.909674000000	2.754898000000	22.704183000000
C	3.744821000000	11.406099000000	16.479934000000
C	3.133276000000	10.097544000000	16.001718000000
O	2.416637000000	9.379542000000	16.756511000000
C	5.300493000000	11.422882000000	16.415491000000
C	5.952292000000	10.334390000000	17.216255000000
N	6.614977000000	10.588088000000	18.412344000000
C	6.042451000000	8.966797000000	17.025340000000
C	7.080290000000	9.414118000000	18.906494000000
N	6.756829000000	8.407700000000	18.081337000000
H	3.354711000000	12.233276000000	15.872057000000
H	5.629048000000	11.369007000000	15.370118000000
H	5.648736000000	12.393502000000	16.784741000000

H	5.660571000000	8.357322000000	16.225735000000
H	7.623018000000	9.309561000000	19.828384000000
H	6.900611000000	11.523370000000	18.795671000000
N	3.431834000000	9.729888000000	14.735499000000
C	3.102336000000	8.433731000000	14.134485000000
C	4.453370000000	7.880974000000	13.649174000000
O	5.278403000000	8.658002000000	13.093578000000
C	2.043726000000	8.599976000000	13.007138000000
O	0.830238000000	9.156138000000	13.554195000000
C	1.766662000000	7.289677000000	12.260322000000
H	4.066417000000	10.291174000000	14.178937000000
H	2.678656000000	7.802034000000	14.919677000000
H	2.420619000000	9.353208000000	12.306828000000
H	0.288604000000	8.460989000000	14.011343000000
H	0.975179000000	7.457357000000	11.524002000000
H	2.659905000000	6.935500000000	11.731476000000
H	1.425569000000	6.515085000000	12.957126000000
N	4.730918000000	6.584385000000	13.924646000000
C	6.107299000000	6.061433000000	13.819743000000
C	6.160697000000	4.554389000000	14.113527000000
C	7.539104000000	4.090436000000	14.626054000000
C	7.753429000000	4.252036000000	16.132941000000
O	8.397529000000	3.353322000000	16.744120000000
O	7.251556000000	5.331312000000	16.690672000000
H	4.025791000000	5.991125000000	14.360360000000
H	6.733180000000	6.592045000000	14.544712000000
H	5.899834000000	3.992270000000	13.206684000000
H	5.403805000000	4.299845000000	14.864127000000
H	7.704233000000	3.034954000000	14.394546000000
H	8.344141000000	4.651147000000	14.128742000000
C	5.629899000000	-1.697573000000	16.130423000000
C	6.338750000000	-0.378118000000	16.492406000000
C	7.729678000000	-0.274778000000	15.837459000000
C	5.468109000000	0.837257000000	16.117587000000
H	5.483138000000	-1.769111000000	15.043376000000
H	6.479462000000	-0.359215000000	17.585617000000
H	8.378347000000	-1.103657000000	16.148315000000
H	7.641707000000	-0.316878000000	14.742384000000
H	8.219593000000	0.669888000000	16.101145000000
H	5.950881000000	1.769969000000	16.429386000000
H	4.481738000000	0.770605000000	16.593861000000
H	5.321609000000	0.876616000000	15.027864000000
C	-3.946239000000	5.592278000000	20.076174000000
C	-3.796947000000	4.101405000000	19.726975000000
C	-2.626424000000	3.390224000000	20.449887000000
C	-2.735877000000	1.861616000000	20.274309000000
C	-1.254234000000	3.888788000000	19.951741000000
H	-3.051558000000	6.171580000000	19.823457000000
H	-4.732993000000	3.580644000000	19.979311000000
H	-3.660750000000	3.978638000000	18.641316000000
H	-2.709620000000	3.620586000000	21.524865000000
H	-1.927537000000	1.347422000000	20.815405000000
H	-3.688728000000	1.479348000000	20.661214000000
H	-2.666375000000	1.601881000000	19.209935000000
H	-1.146105000000	4.973276000000	20.056534000000
H	-0.440656000000	3.410735000000	20.515166000000
H	-1.128352000000	3.631230000000	18.891282000000
C	8.512831000000	10.676240000000	13.348110000000
C	8.537236000000	11.998211000000	14.115286000000
O	8.063573000000	13.032775000000	13.583136000000
C	8.439714000000	9.349563000000	14.148973000000
C	9.747695000000	8.912060000000	14.779773000000
O	10.038464000000	9.346977000000	15.971695000000
O	10.524202000000	8.118080000000	14.116406000000
H	7.636728000000	10.727115000000	12.695807000000
H	7.679670000000	9.432175000000	14.929829000000
H	8.122843000000	8.569499000000	13.453830000000
N	9.140056000000	12.008994000000	15.344907000000
C	9.367157000000	13.250117000000	16.082930000000
C	10.838143000000	13.669952000000	16.165889000000
O	11.177212000000	14.657118000000	16.867169000000
H	9.429478000000	11.128294000000	15.773682000000
H	8.815961000000	14.043486000000	15.569235000000
N	11.716476000000	12.920704000000	15.455750000000
C	13.156455000000	13.140535000000	15.428350000000
C	13.855750000000	11.785968000000	15.348159000000
O	13.302657000000	11.092924000000	14.203023000000
H	11.387549000000	12.164173000000	14.870423000000
H	13.445224000000	13.691049000000	16.328042000000
H	13.676713000000	11.223556000000	16.277267000000
H	14.940974000000	11.931113000000	15.240141000000
H	13.341221000000	10.105186000000	14.307741000000
C	6.708302000000	16.180873000000	20.526524000000
C	6.771849000000	16.416030000000	22.034742000000
O	7.099754000000	17.539575000000	22.511921000000
C	7.675540000000	15.134678000000	19.921279000000
C	7.288618000000	13.669170000000	20.079971000000
O	7.784863000000	12.834546000000	19.211774000000
O	6.560018000000	13.282235000000	21.064756000000
H	5.676867000000	15.912281000000	20.261502000000
H	7.785292000000	15.326404000000	18.849291000000
H	8.684914000000	15.263721000000	20.337084000000
N	6.416884000000	15.368126000000	22.828060000000
C	6.188295000000	15.523690000000	24.261445000000
C	7.422160000000	15.809307000000	25.126522000000
O	7.278735000000	16.284986000000	26.274495000000
H	6.249927000000	14.473518000000	22.349951000000
H	5.500804000000	16.354784000000	24.449548000000
H	5.717617000000	14.606599000000	24.630089000000
N	8.625037000000	15.505926000000	24.566023000000
C	9.914056000000	15.771682000000	25.204153000000
C	10.522994000000	17.122122000000	24.766750000000
O	11.702880000000	17.412450000000	25.099795000000
C	10.905560000000	14.624272000000	24.921667000000
C	10.530198000000	13.330985000000	25.665069000000
O	10.678089000000	13.303733000000	26.917136000000
O	10.078409000000	12.332481000000	24.934140000000
H	8.629977000000	15.094825000000	23.642142000000
H	9.738855000000	15.827459000000	26.284475000000
H	10.964704000000	14.440765000000	23.841681000000
H	11.886611000000	14.959150000000	25.269246000000
N	9.736534000000	17.946007000000	24.025500000000
C	10.217743000000	19.230187000000	23.519070000000
C	10.096109000000	19.341656000000	21.987409000000
C	10.833938000000	18.284214000000	21.177881000000
C	12.006269000000	17.655998000000	21.633610000000
C	10.331695000000	17.919862000000	19.914002000000
C	12.649249000000	16.690900000000	20.845837000000
C	10.970012000000	16.955858000000	19.126487000000
C	12.135259000000	16.335134000000	19.594504000000
H	8.797315000000	17.665929000000	23.755607000000
H	11.251579000000	19.334808000000	23.856405000000
H	9.031845000000	19.300978000000	21.722383000000
H	10.451102000000	20.345293000000	21.701253000000
H	12.402877000000	17.882560000000	22.618077000000
H	9.422043000000	18.395256000000	19.553057000000
H	13.545714000000	16.207473000000	21.224333000000
H	10.567997000000	16.654673000000	18.165507000000
H	12.608903000000	15.576024000000	18.981912000000
C	2.368800000000	-0.557250000000	19.967912000000
C	1.589731000000	-0.550351000000	18.668463000000
O	2.146787000000	-0.757574000000	17.551683000000
C	3.593069000000	0.395026000000	19.939297000000
C	4.468710000000	0.167996000000	21.194603000000
C	3.146988000000	1.862426000000	19.794696000000
C	5.808470000000	0.922435000000	21.173186000000
H	1.717344000000	-0.302219000000	20.814743000000
H	4.186386000000	0.123383000000	19.055104000000
H	4.668391000000	-0.909621000000	21.290766000000
H	3.896219000000	0.457624000000	22.089615000000
H	6.412733000000	0.663394000000	22.050133000000
H	6.389194000000	0.665816000000	20.278289000000

H	5.670620000000	2.008746000000	21.178063000000
H	2.523748000000	2.001700000000	18.903418000000
H	4.005970000000	2.533381000000	19.700085000000
H	2.564704000000	2.181878000000	20.670042000000
N	0.248776000000	-0.313537000000	18.748022000000
C	-0.626225000000	-0.359090000000	17.568101000000
C	-0.574313000000	0.929920000000	16.737440000000
O	-1.531033000000	1.745074000000	16.724560000000
H	-0.163045000000	-0.049486000000	19.631440000000
H	-0.325387000000	-1.214173000000	16.954120000000
N	0.581732000000	1.113244000000	16.046657000000
C	0.832598000000	2.344847000000	15.298013000000
C	2.051543000000	2.180481000000	14.376629000000
C	2.562018000000	3.525912000000	13.892248000000
O	2.785979000000	4.468808000000	14.700482000000
N	2.792354000000	3.658876000000	12.562635000000
H	1.337073000000	0.447579000000	16.215137000000
H	1.009203000000	3.179595000000	15.984658000000
H	1.816015000000	1.515456000000	13.538549000000
H	2.874520000000	1.717538000000	14.939004000000
H	2.605823000000	2.916496000000	11.907958000000
H	3.150129000000	4.536425000000	12.211376000000
C	-2.816401000000	6.112438000000	15.386140000000
C	-1.626036000000	7.026704000000	15.645879000000
O	-0.744803000000	7.209621000000	14.756422000000
C	-2.422641000000	4.783538000000	14.721055000000
H	-3.368799000000	5.929544000000	16.315478000000
H	-1.927853000000	4.109475000000	15.428178000000
H	-3.312740000000	4.267197000000	14.346040000000
H	-1.750675000000	4.977041000000	13.879088000000
N	-1.604313000000	7.642602000000	16.853063000000
C	-0.554937000000	8.559061000000	17.303256000000
C	-0.117856000000	8.107730000000	18.703091000000
O	-0.917601000000	7.493192000000	19.467783000000
C	-1.017681000000	10.055828000000	17.316885000000
C	-1.294057000000	10.559306000000	15.888432000000
C	-2.231195000000	10.291943000000	18.235979000000
H	-2.252111000000	7.351082000000	17.575171000000
H	0.286182000000	8.479827000000	16.611116000000
H	-0.160988000000	10.616708000000	17.716524000000
H	-0.427269000000	10.428343000000	15.232949000000
H	-1.554025000000	11.624710000000	15.909601000000
H	-2.140790000000	10.021984000000	15.443474000000
H	-2.055360000000	9.937884000000	19.256877000000
H	-2.465759000000	11.361754000000	18.280289000000
H	-3.118970000000	9.776630000000	17.847626000000
N	1.145459000000	8.435741000000	19.059474000000
C	1.669678000000	8.165704000000	20.400000000000
C	2.180997000000	6.714097000000	20.608434000000
C	3.350926000000	6.326053000000	19.756390000000
N	3.234987000000	5.660477000000	18.526486000000
C	4.703538000000	6.530921000000	19.933657000000
C	4.493145000000	5.493208000000	18.017526000000
N	5.402813000000	6.011902000000	18.845235000000
H	1.750064000000	8.877832000000	18.361388000000
H	0.871775000000	8.354109000000	21.125391000000
H	1.340085000000	6.029343000000	20.453621000000
H	2.469738000000	6.621218000000	21.663107000000
H	5.228417000000	7.016756000000	20.738123000000
H	4.704667000000	5.024922000000	17.073768000000
C	10.163318000000	0.218582000000	19.321240000000
C	9.974648000000	1.698611000000	19.685165000000
N	10.313771000000	2.614252000000	18.587026000000
C	11.552220000000	3.022734000000	18.289174000000
N	12.624276000000	2.596588000000	19.006370000000
N	11.779456000000	3.866329000000	17.264045000000
H	11.207287000000	0.007950000000	19.066435000000
H	9.540392000000	-0.050112000000	18.461625000000
H	8.929758000000	1.907573000000	19.929501000000
H	10.561405000000	1.957765000000	20.574491000000
H	9.533269000000	3.015362000000	18.048638000000

H	12.561498000000	2.336961000000	19.995287000000
H	13.525451000000	2.939184000000	18.707512000000
H	11.033235000000	4.218265000000	16.662909000000
H	12.570814000000	4.506573000000	17.357422000000
Fe	7.502017000000	6.418777000000	18.383794000000
O	8.268978000000	4.965639000000	19.764321000000
O	9.240605000000	7.163023000000	17.807425000000
C	8.141239000000	5.744942000000	20.799871000000
O	7.689273000000	6.945085000000	20.601332000000
C	8.536343000000	5.264709000000	22.170573000000
C	7.772643000000	5.954847000000	23.309094000000
H	8.400725000000	4.177422000000	22.193819000000
H	9.614032000000	5.447106000000	22.298515000000
H	6.696598000000	5.752439000000	23.250013000000
H	7.911498000000	7.038397000000	23.263767000000
N	12.105228000000	7.850313000000	16.982248000000
C	12.412431000000	7.999742000000	18.450072000000
C	13.570923000000	7.010169000000	18.827708000000
O	13.557747000000	5.864012000000	18.257313000000
C	11.201413000000	7.696365000000	19.297772000000
C	10.646580000000	8.647781000000	20.311248000000
C	10.684264000000	10.154830000000	19.928526000000
N	9.688550000000	10.975131000000	20.637222000000
C	9.788192000000	11.359061000000	21.941381000000
N	10.820842000000	11.027356000000	22.718519000000
N	8.819438000000	12.117364000000	22.491062000000
O	14.380527000000	7.447933000000	19.711326000000
H	12.770332000000	9.018187000000	18.606705000000
H	9.214938000000	8.082345000000	17.460312000000
H	10.955892000000	6.648018000000	19.417003000000
H	11.197903000000	8.496591000000	21.258349000000
H	9.614903000000	8.346977000000	20.526249000000
H	10.454135000000	10.267131000000	18.864489000000
H	11.690570000000	10.565032000000	20.089014000000
H	9.023899000000	11.507811000000	20.069542000000
H	11.603113000000	10.427986000000	22.473621000000
H	10.789201000000	11.423818000000	23.677407000000
H	7.946832000000	12.331620000000	22.012060000000
H	8.977621000000	12.384611000000	23.475216000000
H	12.911236000000	8.022492000000	16.356298000000
H	11.348157000000	8.518402000000	16.668192000000
H	11.748725000000	6.898209000000	16.754771000000
O	13.102200000000	8.390195000000	14.474445000000
H	12.109605000000	8.264400000000	14.268509000000
H	13.657863000000	7.856662000000	13.879772000000
O	10.467066000000	5.851276000000	15.887186000000
H	9.867805000000	6.261511000000	16.595201000000
H	10.413854000000	6.373666000000	15.055089000000
H	9.882172000000	-0.420136000000	20.167426000000
H	2.720979000000	-1.585316000000	20.128344000000
H	-1.654213000000	-0.496845000000	17.901820000000
H	-0.062874000000	2.594138000000	14.720594000000
H	4.644933000000	-1.759481000000	16.608552000000
H	6.223791000000	-2.566005000000	16.442465000000
H	-4.799465000000	6.033628000000	19.545178000000
H	-4.127004000000	5.723283000000	21.151196000000
H	2.484633000000	8.871395000000	20.585020000000
H	-3.492040000000	6.667012000000	14.719058000000
H	6.501515000000	6.283808000000	12.822058000000
H	3.410912000000	11.558466000000	17.508926000000
H	6.926027000000	17.151848000000	20.075113000000
H	9.393569000000	10.659665000000	12.689804000000
H	15.147492000000	4.186406000000	20.369013000000
H	10.823206000000	4.045318000000	24.873822000000
H	13.436799000000	13.745872000000	14.555567000000
H	8.981055000000	13.174757000000	17.106763000000
H	9.639779000000	20.045472000000	23.975614000000
H	8.132474000000	5.600863000000	24.281485000000
C	2.003385000000	5.195213000000	17.886822000000
H	2.247016000000	4.850807000000	16.880992000000
H	1.554719000000	4.377959000000	18.460277000000

H 1.285249000000 6.011196000000 17.813557000000

$^5P_{me}$ – VioC MeHis Product bound state

C 11.631838000000 4.681801000000 25.719405000000
C 11.775286000000 6.012046000000 24.997853000000
O 11.568011000000 6.130131000000 23.763484000000
C 12.922140000000 3.821973000000 25.813252000000
O 13.419805000000 3.457598000000 24.497386000000
C 12.656111000000 2.527403000000 26.581563000000
H 11.255141000000 4.847000000000 26.737768000000
H 13.700197000000 4.403754000000 26.328601000000
H 14.137928000000 4.099071000000 24.221559000000
H 13.575677000000 1.938416000000 26.644090000000
H 12.295404000000 2.729890000000 27.596747000000
H 11.903024000000 1.929121000000 26.056191000000
N 12.150555000000 7.098982000000 25.744232000000
C 12.129443000000 8.460571000000 25.172889000000
C 13.196364000000 8.623967000000 24.078388000000
O 13.002509000000 9.357181000000 23.074599000000
C 12.281462000000 9.518921000000 26.291511000000
C 11.232909000000 9.425794000000 27.432594000000
C 11.506542000000 10.531624000000 28.473306000000
C 9.789410000000 9.526811000000 26.899661000000
H 12.194691000000 7.019883000000 26.750655000000
H 11.174503000000 8.605955000000 24.658542000000
H 13.293528000000 9.448151000000 26.716890000000
H 12.191973000000 10.507518000000 25.827445000000
H 11.351402000000 8.450558000000 27.943697000000
H 9.524947000000 8.675170000000 26.258705000000
H 9.665930000000 10.452703000000 26.324203000000
H 9.078256000000 9.542828000000 27.734576000000
H 12.544688000000 10.494790000000 28.831309000000
H 11.312266000000 11.518456000000 28.034179000000
H 10.847166000000 10.407006000000 29.341647000000
N 14.363781000000 7.954108000000 24.267654000000
C 15.291693000000 7.684101000000 23.158534000000
C 15.186949000000 6.175783000000 22.928292000000
O 15.170309000000 5.393766000000 23.943555000000
C 16.740297000000 8.185481000000 23.421014000000
C 16.773408000000 9.725468000000 23.418636000000
C 17.351974000000 7.617953000000 24.716201000000
H 14.402978000000 7.282152000000 25.024856000000
H 14.892316000000 8.209205000000 22.285673000000
H 17.337980000000 7.824635000000 22.569368000000
H 17.313570000000 6.525064000000 24.740279000000
H 18.397752000000 7.934250000000 24.807678000000
H 16.814556000000 7.998063000000 25.593936000000
H 17.798266000000 10.087900000000 23.561597000000
H 16.155399000000 10.125940000000 24.230193000000
H 16.393274000000 10.134015000000 22.475835000000
N 15.038067000000 5.747136000000 21.670518000000
C 14.617223000000 4.366636000000 21.409717000000
C 13.092966000000 4.267681000000 21.608597000000
O 12.677542000000 2.874132000000 21.767724000000
H 14.847309000000 6.407611000000 20.892178000000
H 15.129607000000 3.692875000000 22.101006000000
H 12.779185000000 4.841283000000 22.479169000000
H 12.569439000000 4.664521000000 20.739725000000
H 12.763591000000 2.665628000000 22.729459000000
C 3.774544000000 11.279481000000 16.542365000000
C 3.185591000000 9.970727000000 16.035997000000
O 2.482657000000 9.222311000000 16.775938000000
C 5.326465000000 11.347212000000 16.432845000000
C 6.030527000000 10.267098000000 17.198349000000
N 6.646738000000 10.509628000000 18.422128000000
C 6.199527000000 8.915918000000 16.958318000000
C 7.155852000000 9.341631000000 18.885978000000
N 6.911447000000 8.351060000000 18.013746000000
H 3.338980000000 12.113384000000 15.975590000000
H 5.625395000000 11.313402000000 15.377565000000
H 5.656767000000 12.322419000000 16.807105000000
H 5.879604000000 8.327192000000 16.117328000000
H 7.675683000000 9.227153000000 19.819920000000
H 6.887455000000 11.451280000000 18.819463000000
N 3.479426000000 9.643190000000 14.758840000000
C 3.156657000000 8.365784000000 14.118710000000
C 4.513201000000 7.826294000000 13.630700000000
O 5.375242000000 8.632151000000 13.180751000000
C 2.099095000000 8.565939000000 12.994924000000
O 0.896760000000 9.134361000000 13.553677000000
C 1.800163000000 7.272165000000 12.227455000000
H 4.121444000000 10.216174000000 14.223656000000
H 2.731431000000 7.709521000000 14.882001000000
H 2.486646000000 9.324866000000 12.306242000000
H 0.345765000000 8.443136000000 14.005537000000
H 1.017928000000 7.467992000000 11.488197000000
H 2.689850000000 6.903746000000 11.702659000000
H 1.439508000000 6.494481000000 12.910583000000
N 4.763157000000 6.507466000000 13.799453000000
C 6.136686000000 5.981643000000 13.689817000000
C 6.187647000000 4.480572000000 14.014137000000
C 7.579707000000 4.016292000000 14.505359000000
C 7.819820000000 4.227230000000 16.003268000000
O 8.061985000000 3.213350000000 16.722480000000
O 7.806089000000 5.470966000000 16.446241000000
H 4.023479000000 5.870575000000 14.093283000000
H 6.769202000000 6.528521000000 14.397146000000
H 5.909525000000 3.900217000000 13.124221000000
H 5.439911000000 4.235475000000 14.778482000000
H 7.705938000000 2.948435000000 14.313198000000
H 8.369942000000 4.548919000000 13.959303000000
C 5.410359000000 -1.796992000000 16.369929000000
C 6.130324000000 -0.457033000000 16.614864000000
C 7.552037000000 -0.451678000000 16.021015000000
C 5.304774000000 0.723687000000 16.066433000000
H 5.308533000000 -1.987020000000 15.292001000000
H 6.221471000000 -0.317043000000 17.704403000000
H 8.167465000000 -1.250442000000 16.454671000000
H 7.515198000000 -0.614684000000 14.934408000000
H 8.047167000000 0.508957000000 16.202838000000
H 5.797462000000 1.675906000000 16.291738000000
H 4.299210000000 0.727650000000 16.505871000000
H 5.202394000000 0.640016000000 14.974008000000
C -3.851694000000 5.172602000000 19.509693000000
C -3.577076000000 3.666819000000 19.661297000000
C -2.563032000000 3.320481000000 20.779598000000
C -2.588629000000 1.808172000000 21.079970000000
C -1.135251000000 3.781838000000 20.421084000000
H -2.934805000000 5.738855000000 19.311899000000
H -4.526519000000 3.152653000000 19.875532000000
H -3.204521000000 3.252399000000 18.712708000000
H -2.873353000000 3.849051000000 21.695742000000
H -1.863579000000 1.542686000000 21.860736000000
H -3.580013000000 1.481707000000 21.417146000000
H -2.349609000000 1.236114000000 20.171353000000
H -1.081065000000 4.864419000000 20.265789000000
H -0.428739000000 3.520787000000 21.220652000000
H -0.803330000000 3.291343000000 19.494987000000
C 8.693064000000 11.090677000000 13.306690000000
C 8.889629000000 12.389198000000 14.088427000000
O 8.649551000000 13.491001000000 13.538483000000
C 8.433846000000 9.791635000000 14.112630000000
C 9.689895000000 9.185135000000 14.705367000000
O 10.005492000000 9.477845000000 15.934040000000
O 10.415720000000 8.412567000000 13.962264000000
H 7.844394000000 11.270223000000 12.640151000000
H 7.720819000000 9.982930000000 14.917487000000
H 7.981278000000 9.060457000000 13.438438000000
N 9.369719000000 12.289365000000 15.367967000000
C 9.679333000000 13.469361000000 16.171942000000
C 11.176792000000 13.731757000000 16.359768000000
O 11.567163000000 14.645620000000 17.129666000000
H 9.510755000000 11.372642000000 15.789506000000

H	9.246189000000	14.337048000000	15.665430000000
N	12.017570000000	12.932025000000	15.659874000000
C	13.471443000000	13.016832000000	15.710775000000
C	14.046907000000	11.611683000000	15.554856000000
O	13.483925000000	11.060289000000	14.339651000000
H	11.651370000000	12.247663000000	15.011296000000
H	13.765725000000	13.469962000000	16.661557000000
H	13.775758000000	11.002486000000	16.430777000000
H	15.144478000000	11.660935000000	15.499631000000
H	13.440835000000	10.067851000000	14.358036000000
C	6.549637000000	16.124924000000	20.542106000000
C	6.619007000000	16.335642000000	22.054101000000
O	6.910613000000	17.462652000000	22.546250000000
C	7.543981000000	15.121990000000	19.907893000000
C	7.205766000000	13.641526000000	20.044332000000
O	7.715811000000	12.837613000000	19.156089000000
O	6.500492000000	13.216601000000	21.030731000000
H	5.524936000000	15.827787000000	20.281409000000
H	7.631909000000	15.335730000000	18.837846000000
H	8.553138000000	15.276652000000	20.315781000000
N	6.308243000000	15.262569000000	22.831705000000
C	6.080194000000	15.384166000000	24.268642000000
C	7.305368000000	15.697218000000	25.136673000000
O	7.146685000000	16.139054000000	26.296308000000
H	6.167959000000	14.371423000000	22.338471000000
H	5.364607000000	16.187174000000	24.473640000000
H	5.642739000000	14.444761000000	24.621582000000
N	8.517038000000	15.456969000000	24.565914000000
C	9.796704000000	15.764051000000	25.204384000000
C	10.331946000000	17.160385000000	24.816977000000
O	11.487169000000	17.507785000000	25.179500000000
C	10.843450000000	14.681660000000	24.865391000000
C	10.554940000000	13.346578000000	25.573127000000
O	10.776296000000	13.280882000000	26.813721000000
O	10.094273000000	12.361307000000	24.832900000000
H	8.535888000000	15.064607000000	23.634206000000
H	9.632023000000	15.769563000000	26.287567000000
H	10.890894000000	14.537823000000	23.778755000000
H	11.811116000000	15.057268000000	25.207963000000
N	9.512088000000	17.957462000000	24.082599000000
C	9.932637000000	19.272321000000	23.602837000000
C	9.864351000000	19.384574000000	22.067078000000
C	10.689590000000	18.368709000000	21.289658000000
C	11.897499000000	17.839048000000	21.776879000000
C	10.237271000000	17.940103000000	20.027262000000
C	12.624605000000	16.908934000000	21.021357000000
C	10.960281000000	17.010104000000	19.272490000000
C	12.160938000000	16.488494000000	19.770413000000
H	8.592408000000	17.634036000000	23.794636000000
H	10.944857000000	19.436706000000	23.979408000000
H	8.814775000000	19.287497000000	21.761287000000
H	10.175957000000	20.406764000000	21.796265000000
H	12.256980000000	18.111908000000	22.763702000000
H	9.301128000000	18.337748000000	19.640700000000
H	13.547700000000	16.502313000000	21.425217000000
H	10.600281000000	16.662042000000	18.310723000000
H	12.698838000000	15.754353000000	19.181389000000
C	2.274537000000	-0.272481000000	20.033219000000
C	1.460598000000	-0.296967000000	18.757186000000
O	1.975756000000	-0.590461000000	17.639080000000
C	3.470313000000	0.716345000000	19.964424000000
C	4.367859000000	0.539812000000	21.212424000000
C	2.979614000000	2.166572000000	19.795948000000
C	5.684357000000	1.332296000000	21.158738000000
H	1.640890000000	-0.027281000000	20.896123000000
H	4.060642000000	0.443003000000	19.078486000000
H	4.599396000000	-0.529583000000	21.326907000000
H	3.798076000000	0.830775000000	22.108701000000
H	6.309199000000	1.104737000000	22.029888000000
H	6.257878000000	1.079324000000	20.258040000000
H	5.515569000000	2.414011000000	21.149085000000

H	2.358945000000	2.276179000000	18.898591000000
H	3.817353000000	2.863140000000	19.699916000000
H	2.380077000000	2.479233000000	20.662026000000
N	0.137553000000	0.018030000000	18.857626000000
C	-0.774525000000	-0.037892000000	17.706842000000
C	-0.669581000000	1.189349000000	16.791574000000
O	-1.584123000000	2.048879000000	16.736454000000
H	-0.233894000000	0.340076000000	19.739986000000
H	-0.552909000000	-0.947643000000	17.139065000000
N	0.478903000000	1.260936000000	16.067566000000
C	0.775168000000	2.415885000000	15.220758000000
C	1.929509000000	2.099238000000	14.255187000000
C	2.479128000000	3.366915000000	13.626743000000
O	2.805758000000	4.356930000000	14.336388000000
N	2.620045000000	3.380849000000	12.277615000000
H	1.202623000000	0.570131000000	16.273132000000
H	1.045046000000	3.282429000000	15.833507000000
H	1.608518000000	1.379374000000	13.494408000000
H	2.756561000000	1.638479000000	14.813258000000
H	2.349085000000	2.600371000000	11.701374000000
H	2.984704000000	4.209437000000	11.829138000000
C	-2.833637000000	6.197775000000	15.298544000000
C	-1.611591000000	7.049046000000	15.613327000000
O	-0.695149000000	7.209967000000	14.755011000000
C	-2.478575000000	4.863356000000	14.622997000000
H	-3.422410000000	6.024476000000	16.206867000000
H	-2.019299000000	4.165827000000	15.331162000000
H	-3.381805000000	4.385502000000	14.228369000000
H	-1.787718000000	5.041588000000	13.792875000000
N	-1.595593000000	7.637963000000	16.834283000000
C	-0.513837000000	8.491591000000	17.326400000000
C	-0.071021000000	7.950515000000	18.693423000000
O	-0.866364000000	7.288132000000	19.420195000000
C	-0.925372000000	9.999128000000	17.418137000000
C	-1.191611000000	10.578791000000	16.016689000000
C	-2.124700000000	10.229368000000	18.356865000000
H	-2.268622000000	7.350304000000	17.533990000000
H	0.314473000000	8.422708000000	16.618073000000
H	-0.049101000000	10.511736000000	17.839307000000
H	-0.337620000000	10.436650000000	15.346152000000
H	-1.401470000000	11.653080000000	16.086345000000
H	-2.065935000000	10.100749000000	15.557604000000
H	-1.955278000000	9.814749000000	19.356078000000
H	-2.320204000000	11.302762000000	18.461459000000
H	-3.033143000000	9.768662000000	17.948284000000
N	1.195936000000	8.256444000000	19.059547000000
C	1.748567000000	7.872205000000	20.359787000000
C	2.284853000000	6.416040000000	20.422716000000
C	3.467498000000	6.119873000000	19.550244000000
N	3.379200000000	5.681881000000	18.218970000000
C	4.819818000000	6.199500000000	19.818170000000
C	4.652270000000	5.525029000000	17.745792000000
N	5.547653000000	5.832766000000	18.686134000000
H	1.796409000000	8.733800000000	18.381961000000
H	0.959762000000	7.977994000000	21.111157000000
H	1.452601000000	5.737585000000	20.202098000000
H	2.574997000000	6.224387000000	21.463405000000
H	5.330393000000	6.510439000000	20.714021000000
H	4.877470000000	5.216372000000	16.741108000000
C	10.446150000000	0.089775000000	19.409313000000
C	9.920740000000	1.513048000000	19.647301000000
N	10.096988000000	2.392378000000	18.483676000000
C	11.223121000000	3.073903000000	18.223636000000
N	12.330494000000	2.953876000000	18.985489000000
N	11.294288000000	3.897371000000	17.160025000000
H	11.519964000000	0.098350000000	19.194325000000
H	9.932087000000	-0.374500000000	18.560886000000
H	8.846863000000	1.492721000000	19.853282000000
H	10.400411000000	1.959528000000	20.527301000000
H	9.259296000000	2.686692000000	17.969245000000
H	12.319737000000	2.556327000000	19.925177000000

H	13.078451000000	3.609949000000	18.778796000000
H	10.517330000000	4.029942000000	16.533885000000
H	12.024616000000	4.610615000000	17.165385000000
Fe	7.641270000000	6.385429000000	18.373897000000
O	8.681483000000	5.081091000000	19.675324000000
O	9.669593000000	7.629212000000	18.138879000000
C	8.369204000000	5.695491000000	20.788498000000
O	7.721972000000	6.808937000000	20.726295000000
C	8.795360000000	5.098412000000	22.107755000000
C	7.890453000000	5.495905000000	23.281391000000
H	8.841874000000	4.010468000000	21.984222000000
H	9.822330000000	5.429081000000	22.320517000000
H	6.867656000000	5.124690000000	23.142299000000
H	7.840162000000	6.584339000000	23.372797000000
N	11.927403000000	7.727507000000	16.777806000000
C	12.177775000000	7.855191000000	18.248052000000
C	13.240901000000	6.831575000000	18.713981000000
O	13.231256000000	5.674799000000	18.149523000000
C	10.834355000000	7.654732000000	19.045056000000
C	10.654202000000	8.647242000000	20.211359000000
C	10.643266000000	10.155217000000	19.821888000000
N	9.656425000000	10.969086000000	20.554565000000
C	9.773837000000	11.341502000000	21.860895000000
N	10.806598000000	10.988219000000	22.628670000000
N	8.822264000000	12.113218000000	22.420969000000
O	13.999319000000	7.234767000000	19.656771000000
H	12.579134000000	8.855903000000	18.420054000000
H	9.540316000000	8.450848000000	17.609453000000
H	10.815789000000	6.639378000000	19.444283000000
H	11.454538000000	8.451567000000	20.935785000000
H	9.709082000000	8.392843000000	20.700284000000
H	10.380748000000	10.277864000000	18.765540000000
H	11.645506000000	10.586732000000	19.948415000000
H	8.994576000000	11.518214000000	20.000810000000
H	11.576572000000	10.377725000000	22.378858000000
H	10.791714000000	11.377144000000	23.588388000000
H	7.939927000000	12.319832000000	21.957335000000
H	9.001245000000	12.388031000000	23.401284000000
H	12.778928000000	7.785078000000	16.198626000000
H	11.296721000000	8.504679000000	16.443414000000
H	11.415144000000	6.855399000000	16.502374000000
O	13.024328000000	8.374255000000	14.320750000000
H	12.029842000000	8.392237000000	14.108925000000
H	13.518861000000	7.875254000000	13.647344000000
O	10.283055000000	6.019470000000	15.401518000000
H	9.388806000000	6.023462000000	15.831508000000
H	10.300875000000	6.671455000000	14.659701000000
H	10.281945000000	-0.530798000000	20.298310000000
H	2.661303000000	-1.287903000000	20.190032000000
H	-1.798630000000	-0.082561000000	18.076806000000
H	-0.130415000000	2.687242000000	14.669817000000
H	4.405418000000	-1.788063000000	16.808561000000
H	5.971870000000	-2.634832000000	16.802593000000
H	-4.557988000000	5.358024000000	18.689878000000
H	-4.295576000000	5.584113000000	20.425701000000
H	2.555470000000	8.570921000000	20.598957000000
H	-3.461122000000	6.795072000000	14.621256000000
H	6.531651000000	6.177158000000	12.685723000000
H	3.469202000000	11.387373000000	17.585731000000
H	6.730845000000	17.110942000000	20.107175000000
H	9.569965000000	10.952725000000	12.658497000000
H	14.907508000000	4.106717000000	20.388724000000
H	10.882591000000	4.103685000000	25.170236000000
H	13.848054000000	13.655323000000	14.900055000000
H	9.220289000000	13.386511000000	17.164263000000
H	9.288365000000	20.047166000000	24.040154000000
H	8.276614000000	5.085467000000	24.221334000000
C	2.168565000000	5.482508000000	17.420448000000
H	2.429200000000	4.913165000000	16.527727000000
H	1.419124000000	4.935763000000	17.997798000000
H	1.754881000000	6.444291000000	17.114642000000
